# Environmental DNA metabarcoding of wild flowers reveals diverse communities of terrestrial arthropods

**DOI:** 10.1002/ece3.4809

**Published:** 2019-02-07

**Authors:** Philip Francis Thomsen, Eva E. Sigsgaard

**Affiliations:** ^1^ Department of Bioscience University of Aarhus Aarhus C Denmark

**Keywords:** arthropods, eDNA, environmental DNA metabarcoding, flowers, grassland, insects, pollinators

## Abstract

Terrestrial arthropods comprise the most species‐rich communities on Earth, and grassland flowers provide resources for hundreds of thousands of arthropod species. Diverse grassland ecosystems worldwide are threatened by various types of environmental change, which has led to decline in arthropod diversity. At the same time, monitoring grassland arthropod diversity is time‐consuming and strictly dependent on declining taxonomic expertise. Environmental DNA (eDNA) metabarcoding of complex samples has demonstrated that information on species compositions can be efficiently and non‐invasively obtained. Here, we test the potential of wild flowers as a novel source of arthropod eDNA. We performed eDNA metabarcoding of flowers from several different plant species using two sets of generic primers, targeting the mitochondrial genes 16S rRNA and COI. Our results show that terrestrial arthropod species leave traces of DNA on the flowers that they interact with. We obtained eDNA from at least 135 arthropod species in 67 families and 14 orders, together representing diverse ecological groups including pollinators, parasitoids, gall inducers, predators, and phytophagous species. Arthropod communities clustered together according to plant species. Our data also indicate that this experiment was not exhaustive, and that an even higher arthropod richness could be obtained using this eDNA approach. Overall, our results demonstrate that it is possible to obtain information on diverse communities of insects and other terrestrial arthropods from eDNA metabarcoding of wild flowers. This novel source of eDNA represents a vast potential for addressing fundamental research questions in ecology, obtaining data on cryptic and unknown species of plant‐associated arthropods, as well as applied research on pest management or conservation of endangered species such as wild pollinators.

## 1 INTRODUCTION

Terrestrial arthropods are experiencing massive decline in Europe as well as globally (Collen, Böhm, Kemp, & Baillie, 2012; Dirzo et al., 2014; Nieto et al., 2014; van Swaay et al., 2010), although only a fraction of the species have been assessed and the majority of insects are still undescribed to science (Stork, 2018). As one example, grassland ecosystems are home to diverse taxonomic and functional groups of terrestrial arthropods, such as pollinators, phytophagous insects, and predators, that use nectar and pollen for food sources, and stem and leaf tissue for food and development. These communities harbor endangered species, since many habitats have disappeared or are under significant threat (Habel et al., 2013; Joern & Laws, 2013). Therefore, extensive efforts are being conducted in order to restore European grassland ecosystems and conserve biodiversity (Silva et al., 2008). For instance, pollinators like bees and butterflies represent an important ecological group that has undergone severe decline in Europe, indicating a dramatic loss of grassland biodiversity (Biesmeijer et al., 2006; Goulson, Nicholls, Botías, & Rotheray, 2015; Potts et al., 2010; van Swaay et al., 2013). The vast majority of flowering plants are pollinated by insects and other animals both in temperate regions and the tropics (Ollerton, Winfree, & Tarrant, 2011). The majority of insect species are herbivores feeding on different parts of plants, and most of these are specialists, relying on one or a few plant species as their main food resource (Price, Denno, Eubanks, Finke, & Kaplan, 2011). However, given the gap in knowledge on existing insect species, and the fact that most species are still undescribed, it is clear that for the majority of plant species in the world, we have only a vague idea about the arthropod communities that they harbor and interact with.

Terrestrial arthropod communities have traditionally been collected and studied using methods, such as Malaise traps and pitfall traps, which are very effective but somewhat cumbersome and potentially invasive methods. In some instances, these techniques fall short of performing efficient and standardized surveys, due to, for example, phenotypic plasticity, closely related species, and difficulties in identifying juvenile stages. Furthermore, morphological identification depends directly on taxonomic expertise, which is in decline (Hopkins & Freckleton, 2002; Sangster & Luksenburg, 2015; Wheeler, Raven, & Wilson, 2004). All such limitations of traditional biodiversity monitoring have created a demand for alternative approaches. Meanwhile, the advance in DNA sequencing technologies continuously provides new means of obtaining biological data (Bohmann et al., 2014; Bush et al., 2017; Creer et al., 2016; Thomsen & Willerslev, 2015). Hence, several new molecular approaches have recently been suggested for obtaining fast and efficient data on arthropod communities and their interactions through non‐invasive genetic techniques. This includes extracting DNA from sources such as bulk samples or *insect soups* (Arribas, Andújar, Hopkins, Shepherd, & Vogler, 2016; Elbrecht et al., 2016; Hajibabaei, Shokralla, Zhou, Singer, & Baird, 2011; Yu et al., 2012), empty leaf mines (Derocles, Evans, Nichols, Evans, & Lunt, 2015), spider webs (Blake, McKeown, Bushell, & Shaw, 2016; Xu, Yen, Bowman, & Turner, 2015), pitcher plant fluid (Bittleston, Baker, Strominger, Pringle, & Pierce, 2015), environmental samples like soil and water (environmental DNA [eDNA]) (Taberlet, Coissac, Hajibabaei, & Rieseberg, 2012; Thomsen et al., 2012; Thomsen & Willerslev, 2015; Zinger et al., 2018), host plant and predatory diet identification from insect DNA extracts (Jurado‐Rivera, Vogler, Reid, Petitpierre, & Gómez‐Zurita, 2009; Paula et al., 2016), and predator scat from bats (Bohmann et al., 2011; Vesterinen, Lilley, Laine, & Wahlberg, 2013). Recently, also DNA from pollen attached to insects has been used for retrieving information on plant–pollinator interactions (Bell et al., 2017; Pornon et al., 2016). Many of such recent studies rely on *DNA metabarcoding*—high‐throughput sequencing of PCR amplicons using generic primers (Taberlet, Bonin, Zinger, & Coissac, 2018; Taberlet et al., 2012).

**Figure 1:**
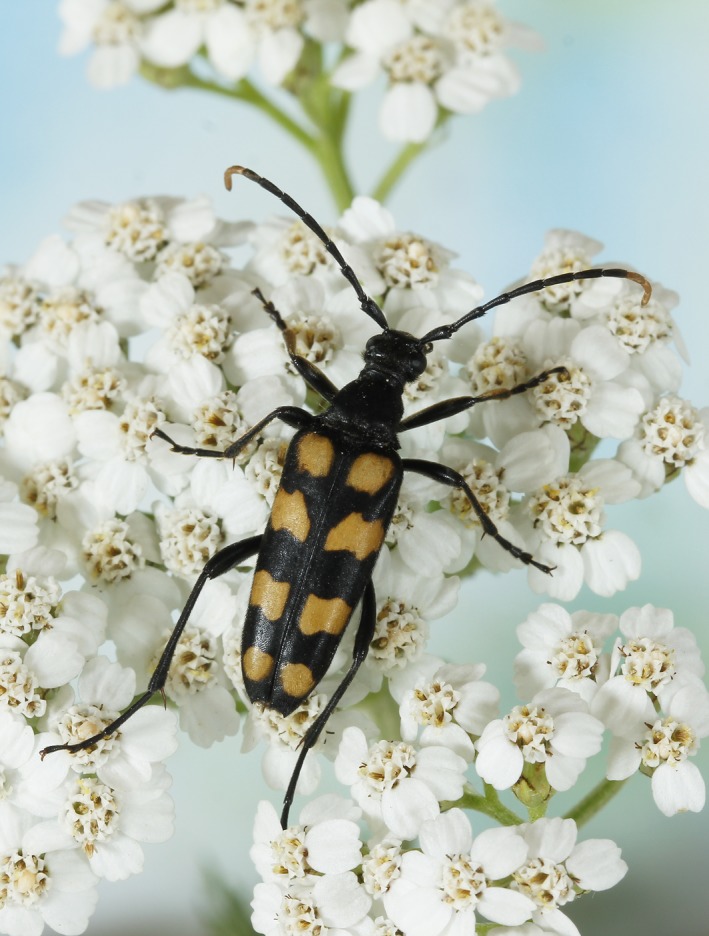
The longhorn beetle *Leptura quadrifasciata*—an example of a flower‐visiting insect found in this study. We show that eDNA from arthropods are deposited on flowers after interactions. Photo: Ole Martin

Given the recent success of eDNA metabarcoding for several different complex sample types, we argue that DNA traces may be more frequent in the environment than one would immediately imagine. Here, we propose the hypothesis that arthropods leave DNA traces on flowers after interaction, and we test the extent to which this source of arthropod eDNA can provide useful information on local species occurrences and communities (Figure 1).

## 2 MATERIALS AND METHODS

### 2.1 Study sites

**Figure 2:**
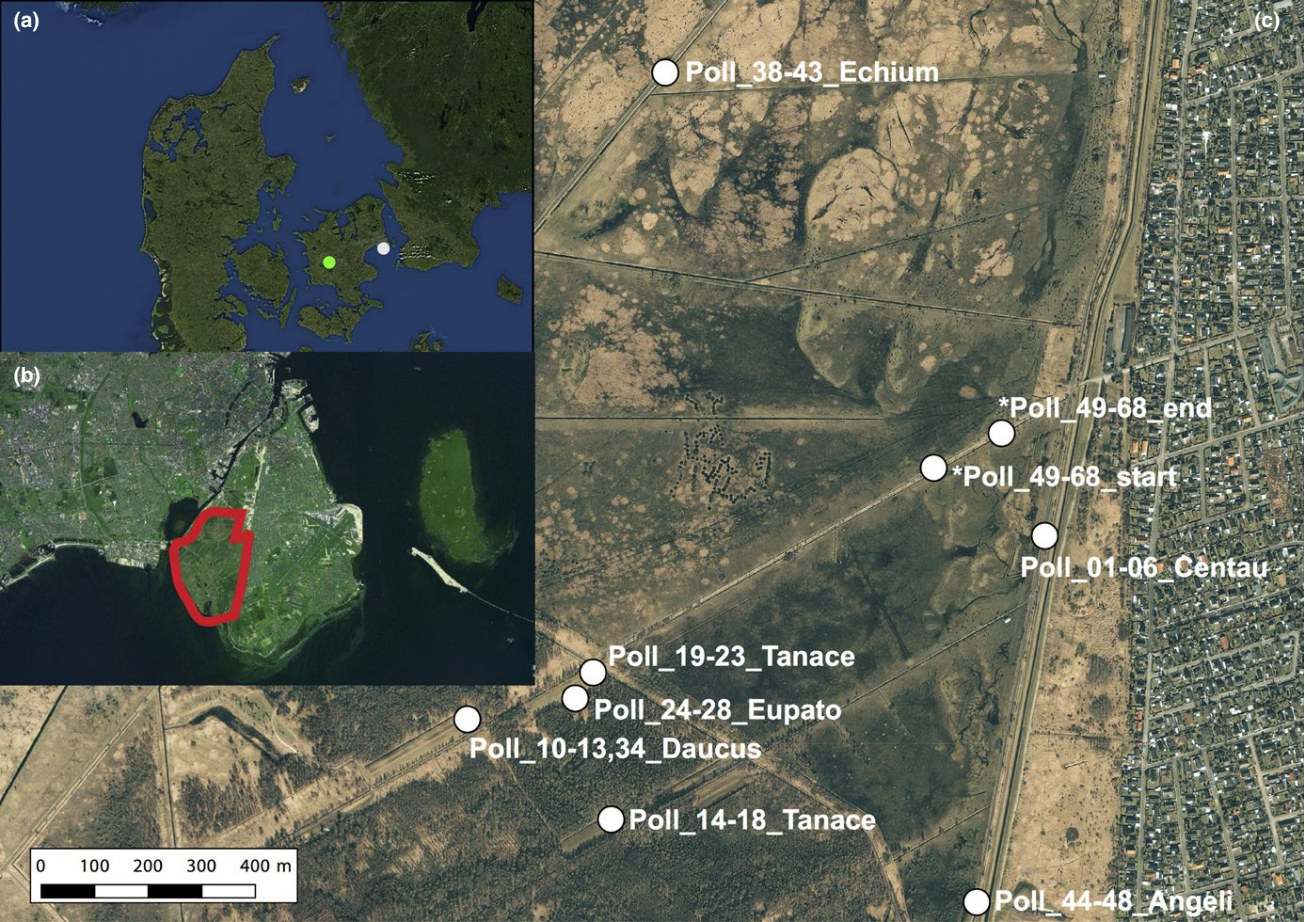
Map of sampling sites. (a) Denmark with the two sampling sites Vestamager (white) and Kristiansminde (green), (b) the island of Amager with the sampling site Vestamager surrounded by the red line, and (c) the plant sample sites within Vestamager. *Transect samples of *Centaurea* and *Daucus* collected interspersed with 10 m distance between each sample

Wild flowers were collected on the seminatural, dry grassland localities of Vestamager and Kristiansminde in Denmark in August 2017 on sunny days with abundance of active arthropods (Figure 2, Supporting information Table S1). The large majority of samples were collected at Vestamager. This locality is old seabed contained in the 1940s and consists mainly of beach meadows, grassland, and young woodland composed of deciduous trees—mainly birch and willow. The area is approximately 2,000 ha and is the southwestern part of the island Amager east of Zealand (Figure 2). Flowers of *Solidago canadensis* were collected at Kristiansminde. This site is a grassland with occurrences of deciduous trees, and surrounded by patches of forest and farmland.

### 2.2 Flower sampling

A total of 56 individual flowers representing seven species were used in the metabarcoding study. Flower heads (Asteraceae: *Centaurea jacea, Tanacetum vulgare*, *Eupatorium cannabinum,* *Solidago canadensis*), umbels (Apiaceae: *Daucus carota*, *Angelica archangelica*) or single complete flowers (Boriganaceae: *Echium vulgare*) (Figure 2, Supporting Information Table S1) were collected. For *Centaurea* and *Daucus*, samples were collected both in discrete areas as well as with the two species interspersed in a transect with 10 m distance between each flower (Figure 2c). Before collection, the flowers were thoroughly inspected to ensure that they did not contain any visible animals. Flowers were collected in sterile plastic tubes (5, 15 or 50 ml) using single‐use sterile nitrile gloves. All flowers were kept in a box with ice blocks immediately after sampling and stored at −20°C after return from the field (max. 5 hr after sampling). They were kept at −20°C until DNA extraction.

### 2.3 DNA extraction

DNA extractions were performed in the laboratories at Centre for GeoGenetics, University of Copenhagen, which are dedicated labs for working with samples of low DNA concentration. Regular decontamination routines are in place, including UV‐light, and pre‐ and post‐PCR work is separated. DNA was extracted using Qiagen DNeasy® Blood & Tissue Kit. Lysis was performed in the plastic tubes containing the flowers by adding 540, 900 or 1,800 μL ATL lysis buffer and 60, 100 or 200 μL proteinase K, respectively, depending on the size of the sample (Table S1). Samples were lysed at 56°C with agitation in a rotor for 3 hr. After lysis, samples were mixed on a vortexer for 10 s and a total of 500, 800, or 1,500 μL lysis mixture were retrieved, respectively. Equal amounts of AL buffer and absolute ethanol, corresponding to the volume of retrieved lysis mixture, were added to the tubes and vortexed thoroughly before the samples were applied to spin columns and spun through the membrane filters over several rounds (700 μL per round). Columns were washed by adding first 600 μL AW1 and then 600 μL AW2 buffers. Finally, DNA was eluted in 2 × 60 μL AE buffer, each time with a 15‐min incubation step at 37°C before spinning. All spinning steps were performed at 10,000 g. DNA extracts were stored at −20°C.

### 2.4 PCR amplification

For DNA metabarcoding, we used two different primer sets targeting two mitochondrial DNA (mtDNA) genes commonly used in arthropod studies: cytochrome c oxidase subunit I (COI) (~211 bp fragment) (Zeale, Butlin, Barker, Lees, & Jones, 2011) and 16S ribosomal RNA (~160 bp fragment) (Elbrecht et al., 2016) (Supporting Information Figure S1). Primers were uniquely tagged. Tags were designed using the OligoTag program (Coissac, 2012), and consisted of six nucleotides with a distance of at least three bases between any two tags. Tags were preceded by two or three random bases; NNN or NN (De Barba et al., 2014), and identical tags were used on the forward and reverse primers for each sample.

PCR reactions were carried out in four replicates per sample, using identical tags for PCR replicates, but a unique tag for each sample. Each PCR batch also contained two PCR replicates of a mock sample (positive control), all four DNA extraction blanks and two PCR blanks (64 in total). The mock sample (positive control) was prepared using tissue‐derived DNA in equimolar concentrations from a spider *Argyroneta aquatica*, a damselfly *Lestes virens*, a bug *Ilyocoris cimicoides*, and two beetles *Cybister lateralimarginalis *and *Dorcus parallelipipedus*.

PCR reactions were performed in 25 µl volumes of 3 µl template DNA, 12.3 µl ddH_2_O, 2.5 µl TaqGold Buffer, 2.5 µl MgCl_2_, 1 µl dNTPs (10 mM each), 1 µl BSA (20 mg/ml), 1 µl of each primer (10 µM), 0.5 µl Hl‐dsDNase (ArcticZymes) (5 U/µL), and 0.2 µl TaqGold enzyme. Before DNA extract was added, the reactions were stored at 37°C for 15 min and 60°C for 15 min for activation and inactivation of the DNAse treatment, respectively. The DNase removes any double‐stranded DNA (contamination) from the reactions before the target DNA template is added. Thermocycling parameters were 95°C for 10 min, 55 cycles of 94°C for 30 s, 54°C for 30 s, 72°C for 1 min, and a final elongation of 72°C for 7 min. Annealing temperature are according to Alberdi, Aizpurua, Gilbert, and Bohmann (2018), and we performed 55 cycles with the initial expectation that arthropod eDNA concentration was low in flower samples.

Fragment sizes were verified on 2% agarose gel stained with GelRed^TM^. Approximately half of the PCR products were verified. For each of the two primer sets, PCR products were mixed in four pools each containing one PCR replicate of each sample (5 µl per replicate), such that the same tag was added only once to each pool. The pools were purified using Qiagen's MinElute PCR purification kit.

### 2.5 Library building and next‐generation sequencing

Library building was performed on the purified pools of PCR products using the TruSeq DNA PCR‐free LT Sample Prep kit (Illumina). A total of eight libraries (corresponding to the four PCR replicates from each sample for each of the two primer sets) were constructed (Supporting Information Figure S1). Each pool thus included one replicate of every sample, four DNA extraction blanks, two PCR blanks, and two mock sample replicates.

The manufacturer's protocol was followed with the exception that samples were incubated with the elution buffer over two rounds of 37°C for 10 min. Approximately 750 ng of PCR product from each pool was used as input for the libraries, and a library blank was included. The concentration and fragment size distribution of the libraries were verified on an Agilent 2100 Bioanalyzer. Libraries were pooled in equimolar concentrations and sequenced on one mid‐output flow cell on an Illumina NextSeq 500 (150 bp paired‐end sequencing) at the Biotech Research and Innovation Centre (BRIC), Dept. of Biology, University of Copenhagen. A spike‐in of 10% PhiX was used to increase complexity in the runs.

### 2.6 High‐throughput sequencing data analyses

Supporting Information Figure S1 shows an overview of the workflow. After de‐multiplexing with a custom python script, Illumina sequences were analyzed using DADA2 (Callahan et al., 2016), in order to clean the data from errors generated during PCR and sequencing (Ficetola et al., 2015; Murray, Coghlan, & Bunce, 2015; Olds et al., 2016) (scripts are available at https://github.com/tobiasgf/Bioinformatic-tools/tree/master/Eva_Sigsgaard_2018). The error filtering in DADA2 is based on error models inferred from the data itself and was therefore done separately for each FASTQ file. Forward and reverse reads were then merged (min. of 5 bp overlap, no mismatches allowed) and likely chimeras were removed with the DADA2 function *removeBimeraDenovo*. Taxonomic assignment for 16S was performed using BLASTn and the NCBI nt database (Benson, Karsch‐Mizrachi, Lipman, Ostell, & Wheeler, 2005), followed by classification using the R package *taxize* and a custom R script (available at https://github.com/tobiasgf/Bioinformatic-tools/tree/master/Eva_Sigsgaard_2018). For the BLAST search, the maximum number of target sequences was set at 40. For the COI dataset, taxonomic assignment was performed using the Barcode of Life Data Systems (BOLD) (Ratnasingham & Hebert, 2007), due to larger taxonomic coverage. The “Species Level Barcode Records” part of the BOLD database was used. Final MOTU assignments were carefully reviewed such that only MOTUs with 99%–100% match (COI) or 100% match (16S) across the entire query sequence and to a single species were assigned to species level. For MOTUs with a 100% match to several species in the same Genus, the MOTU was assigned to Genus level, while MOTUs with 100% match to several genera in the same family were assigned to family level, etc. To produce a conservative estimate of the diversity obtained by eDNA, we excluded taxa found in only a single PCR replicate, but report all taxa obtained as supplementary data for overview.

Illumina raw sequence data are available from the Dryad Digital Repository (https://doi.org/10.5061/dryad.2j151bd).

### 2.7 Accumulation analyses

Species accumulation curves for replicate flower samples and PCR replicates were performed using the function *specaccum* from the R package *vegan* v. 2.4‐6 (Oksanen et al., 2018). The “exact” species accumulation method was used, which finds the mean species richness across sites/replicates.

### 2.8 Rarefaction analyses

Rarefaction curves for the four individual PCR replicates of each sample based on number of taxa as a function of sequencing depth were performed using the function *rarecurve* from *vegan* v. 2.4‐6.

### 2.9 Differentiation analyses

In order to investigate how well the arthropod communities were differentiated according to plant species, we performed a number of analyses. A redundancy analysis (RDA) of arthropod communities in the different flower samples was performed, using the *rda* function in *vegan*, with latitude and longitude of the sample sites as conditioning variables. Additionally, we made a bipartite diagram showing the links between plants and arthropods found in this study, using the R package *bipartite *(Dormann, Gruber, & Fruend, 2008). Finally, heatmap cluster analyses of the arthropod communities in the different plant species were performed using the R package *pheatmaps *(Kolde, 2018). Clustering was set to the average‐linkage method and was done using Raup–Crick distances calculated with the *vegdist* function of the *vegan* R package (Oksanen et al., 2018). Distances were transformed with cube transformation (*n* ^1/3^) to obtain an appropriate scale for the figure.

### 2.10 Faunistic data

We investigated how well the species obtained using eDNA corresponded with faunistic occurrence data. Data on species occurrences and distributions in Denmark were obtained from the extensive national biodiversity web portal Naturbasen (https://www.naturbasen.dk/) and the Danish biodiversity overview project “allearter” (Skipper, 2017) (www.allearter.dk). Furthermore, wider occurrence data on species were obtained from Fauna Europea (Jong et al., 2014) (https://fauna-eu.org/). From Naturbasen, species occurences were retrieved (February 2018) for all species found in Vestamager samples. Occurrence data were retrieved for five spatial levels; (a) the study site of Vestamager, (b) the island of Amager, (c) the wider region of Copenhagen, (d) the island of Zealand, and (e) the rest of Denmark outside Zealand. Only eDNA data for the Vestamager samples were used in the faunistic analyses due to a rich record of occurrence data at this site. Only species for which there was at least one record in Denmark were included in the analyses.

All reported *R* ^2^ values were adjusted to the number of predictors (adjusted *R* ^2^). Species accumulation and differentiation analyses were performed in RStudio v. 1.1.442 (RStudio Team, 2016), while the statistical comparisons with occurrence data were performed in R v. 2.13.1 (R Core Team, 2016).

## 3 RESULTS

### 3.1 DNA metabarcoding reads

A total of 286,678,508 raw reads passing the chastity filter were produced on the Illumina NextSeq 500 platform, of which 129,336,485 were from the COI gene, and 118,129,632 were from the 16S gene. We obtained similar sequencing depth across the eight libraries (PCR replicates): 30,933,265 ± 2,439,162 reads (mean ± SEM) per library. After data cleaning and merging of paired reads, a total of 36,538,959 reads were retained for COI (only including eDNA samples. Four libraries) and 41,155,857 reads were retained for 16S (only including eDNA samples. Four libraries). The samples had similar sequence depth with 183,732 ± 6,604 final reads (mean ± SEM) (16S) and 131,890 ± 6,824 final reads (mean ± SEM) (COI) per sample, respectively. Additionally, from the mock, extraction blanks and PCR blanks, a total of 1,427,826 reads were retained for COI and 1,400,496 reads were retained for 16S.

**Figure 3:**
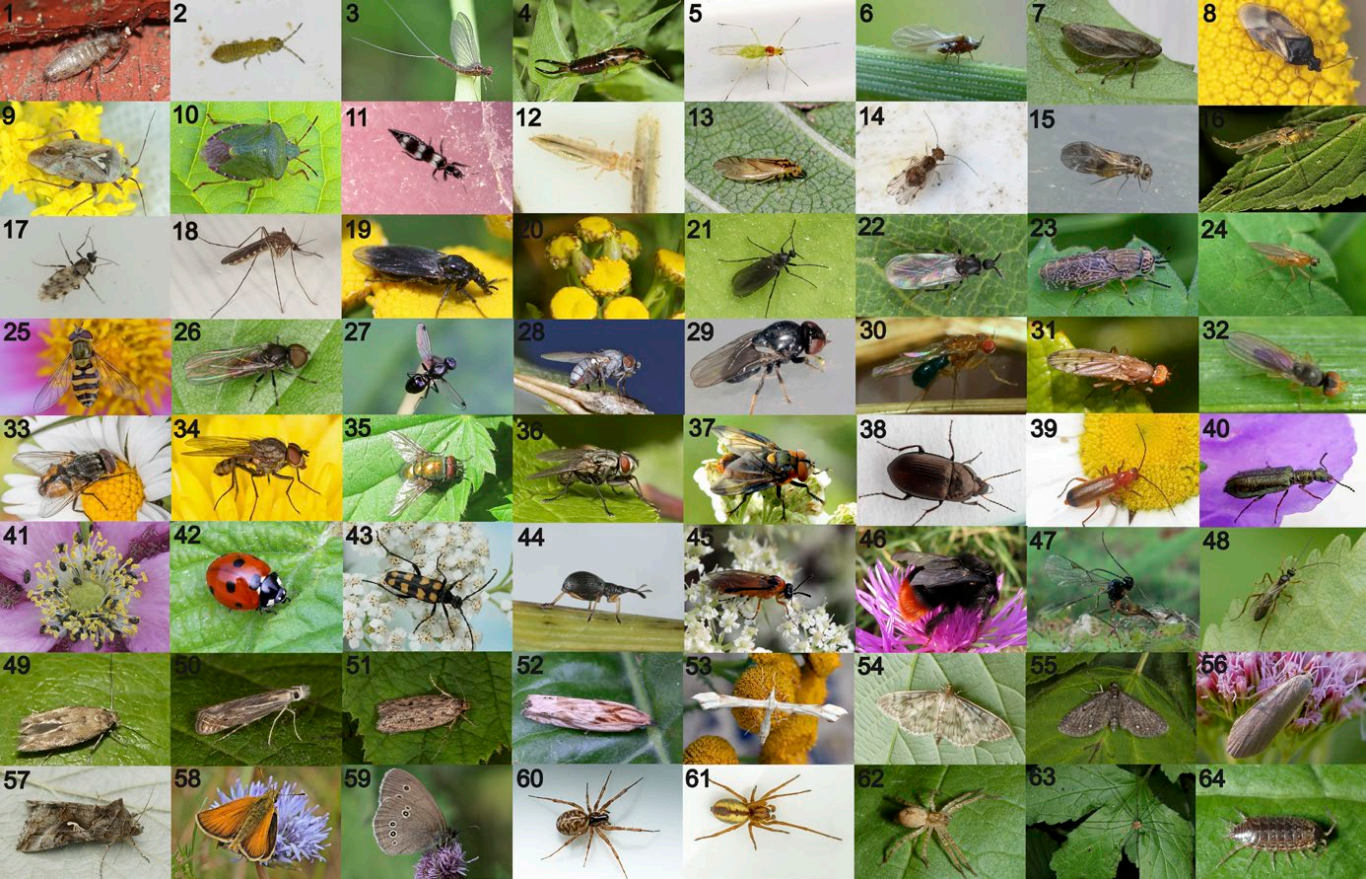
Photos of arthropod families found with eDNA on wild flowers in this study. A representative for each family is shown except the aquatic families Veliidae, Asellidae, and Polyphemidae. *The taxon found in the study is different from the one in the example photo, see Supporting Information Table S2. See the acknowledgments section for photo credits. COLLEMBOLA: (1) Entomobryidae (*Willowsia nigromaculata*), (2) Isotomidae (*Isotoma viridis*), INSECTA: (3) Baetidae (*Cloeon dipterum*), (4) Forficulidae (*Forficula auricularia*), (5) Aphididae (*Euceraphis betulae*), (6) Adelgidae (Pineus pini*), (7) Aphrophoridae (*Philaenus spumarius*), (8) Anthocoridae (*Orius* sp.), (9) Miridae (*Lygus rugulipennis*), (10) Pentatomidae (*Palomena prasina*), (11) Aeolothripidae (*Aeolothrips fasciatus*), (12) Thripidae (Thripidae sp.*), (13) Caeciliusidae (*Valenzuela flavidus*), (14) Ectopsocidae (*Ectopsocus briggsi*), (15) Peripsocidae (*Peripsocus subfasciatus*), (16) Chironomidae (Chironomidae sp.), (17) Ceratopogonidae (Culicoides punctatus*), (18) Culicidae (*Culex* sp.), (19) Bibionidae (*Dilophus febrilis*), (20) Cecidomyiidae (*Rhopalomyia* sp.), (21) Sciaridae (Schwenckfeldina carbonaria), (22) Scatopsidae (Coboldia fuscipes), (23) Tabanidae (Haematopota pluvialis*), (24) Lonchopteridae (*Lonchoptera bifurcata*), (25) Syrphidae (*Syrphus vitripennis*), (26) Pipunculidae (Pipunculidae sp.*), 27 Sepsidae (*Sepsis* sp.*), (28) Chamaemyiidae (*Leucopis* sp.), (29) Chloropidae (*Siphonella oscinina*), (30) Drosophilidae (*Drosophila fenestratum*), (31) Opomyzidae (*Opomyza florum*), (32) Anthomyzidae (*Anthomyza gracilis*), (33) Muscidae (*Musca autumnalis*), (34) Anthomyiidae (*Delia platura*), (35) Calliphoridae (*Lucilia caesar*), (36) Sarcophagidae (*Macronychia* sp.*), (37) Tachinidae (*Phasia hemiptera*), (38) Carabidae (*Amara similata*), (39) Cantharidae (*Rhangonycha fulva*), (40) Melyridae (*Dasytes plumbeus*), (41) Nitidulidae (*Meligethes aeneus*), (42) Coccinellidae (*Coccinella septempunctata*), (43) Cerambycidae (*Leptura quadrifasciata*), (44) Brentidae (*Apion fulvipes*), (45) Tenthredinidae (*Athalia rosae*), (46) Apidae (*Bombus lapidarius*), (47) Braconidae (Praon volucre*), (48) Ichneumonidae (*Promethes sulcator*), (49) Momphidae (*Mompha epilobiella*), (50) Gelechiidae (*Isophrictis striatella*), (51) Oecophoridae (*Hofmannophila pseudospretella*), (52) Tortricidae (*Dichrorampha obscuratana*), (53) Pterophoridae (*Gillmeria ochrodactyla*), (54) Crambidae (*Pleuroptya ruralis*), (55) Geometridae (*Eupithecia tripunctaria*), (56) Erebidae (*Eilema griseola*), (57) Noctuidae (*Autographa gamma*), (58) Hesperiidae (*Thymelicus lineola*), (59) Nymphalidae (*Aphantopus hyperantus*), ARACHNIDA: (60) Linyphiidae (*Neriene clathrata*), (61) Miturgidae (Cheiracanthium erraticum*), (62) Anyphaenidae (*Anyphaena accentuata*), (63) Leiobunidae (*Leiobunum rotundum*), MALACOSTRACA: (64) Philosciidae (*Philoscia muscorum*)

Initial clustering of the reads into MOTUs using the DADA2 pipeline created 1,162 MOTUs for the COI gene, and 843 MOTUs for the 16S gene, respectively. Final data, with MOTUs obtained from at least two independent PCR replicates, yielded 23,517,933 COI reads and 658,159 16S reads from arthropods as well as 1,164,170 COI reads and 1,716,231 16S reads from non‐arthropods, respectively. These final reads represented eDNA sequences from a total of 135 arthropod species in 67 families and 14 orders (Table 1, Figure 3, Supporting Information Tables S2–S4, Figures S2–S3). Several additional taxa were found when including MOTUs obtained in only a single PCR replicate (Table S3). Of the final authentic reads, the most abundant families (more than one million reads) were Thripidae, Geometridae, Cecidomyiidae and Nitidulidae, respectively. Of the COI sequences, 30% belonged to a single species (*Thrips major*), while for 16S the most abundant species was *Meligethes planiusculus*, which represented 51% of the final reads. In addition to arthropod sequences, we found eDNA from other taxa such as snails (*Deroceras agreste *and *Fruticicola fruticum*), and the mammal species fallow deer (*Dama dama*) and horse (*Equus ferus cabellus*), which occur in the area and could thus have come into direct contact with the flowers (Table S3). DNA from cow, pig, dog, human and pike were treated as contaminants. From the 16S gene, as many as 1,154,704 final reads were from human. We believe the pike DNA stems from previous work in the laboratory, while human, cow, pig, dog are all common contaminants.

**Table 1:** Final list of arthropod diversity identified. Number of families, genera, and species in each order are given along with the number of final reads for the two genes and the number of families on each plant species. Besides the read numbers given in the table, 215,194 reads of Lepidoptera spp. that could not be identified to family level were also obtained

Class	Order	Families	Genera	Species	COI reads	16S reads	Combined	Centaurea	Daucus	Tanacetum	Eupatorium	Echium	Angelica	Solidago
Collembola	Entomobryomorpha	2	3	3	55	3,608	3,663	2	2	1			1	
Insecta	Ephemeroptera	1	1	1	1,201	1,557	2,758	1						
Insecta	Dermaptera	1	1	1		120	120		1					
Insecta	Hemiptera	7	16	21	1,008,900	64,093	1,072,993	5	4	3	3	1	4	3
Insecta	Thysanoptera	2	2	3	7,043,572	285	7,043,857	1	1	2	1		1	1
Insecta	Psocoptera	3	3	3	59,874		59,874	2	2	1	2	1	1	1
Insecta	Diptera	22	47	59	5,939,455	39,012	5,978,467	9	17	5	6	2	14	8
Insecta	Coleoptera	7	9	10	2,163,802	510,635	2,674,437	3	6	4	4	1	4	1
Insecta	Hymenoptera	4	6	6	175,214	42	175,256	3	2	1	1		1	
Insecta	Lepidoptera	11	18	21	6,431,747		6,431,747	4	4	4	4	2	3	2
Arachnida	Araneae	3	3	3	692,305		692,305		1				1	1
Arachnida	Opiliones	1	1	1		37,806	37,806	1						1
Malacostraca	Isopoda	2	2	2	1,808	526	2,334	1	1					
Branchiopoda	Diplostraca	1	1	1		475	475	1						
TOTAL	14	67	113	135	23,517,933	658,159	24,176,092	33	41	21	21	7	30	18

In the final trimmed data, the mock samples yielded 1,214,927 COI reads and 876,798 16S reads (Table S5). The vast majority of these sequences matched species added to the mock (COI: *Cybister lateralimarginalis* and *Ilyocoris cimicoides*; 16S: *Argyroneta aquatic*, *Lestes virens*, *Ilyocoris cimicoides* and *Dorcus parallelipipedus*). Low‐abundance reads of some contaminants also occurred in the mock, representing <0.5% of the reads (Table S5). No PCR or extraction blanks gave visible bands on the initial gel images. They were sequenced nonetheless, and yielded 1694 and 445,277 reads (COI and 16S, respectively) from the extraction controls in the final data. These reads were all from human (16S) and Cecidomyiidae spp. (COI). The latter sequence was also found in the eDNA COI data (Cecidomyiidae sp.5), but given the comparatively low read number in the blank and since the sequence only occurred in one of four extraction blanks, this was considered an accidental and rare carryover in the extraction process, and not a general contamination. The PCR blanks gave no reads in the final data.

### 3.2 Arthropod eDNA diversity recovered

Overall, our study uncovered eDNA from several taxonomic and functional groups of arthropods.

#### 3.2.1 Pollinators

The red‐tailed bumblebee (*Bombus lapidarius*) is common in the study area, where it frequently visits flowers. The species was observed on *Centaurea* flowers, which was also the species from where *Bombus lapidarius* eDNA was recovered. Other pollinators such as four species of hoverflies and two species of butterflies were also found with eDNA. The butterfly *Thymelicus lineola* was observed on *Centaurea* flowers in the field, from which the majority of eDNA reads were obtained, and the butterfly *Aphantopus hyperantus* was observed on *Solidago* flowers, also corresponding with the eDNA results. As for hoverflies, eDNA was observed from *Syrphus vitripennis* (on *Daucus* and *Angelica*), *Eristalis pertinax* (on *Centaurea* and *Solidago*), *Platycheirus clypeatus* (on *Daucus* and *Centaurea*), and *Sphaerophoria* sp. (on *Daucus* and *Angelica*).

#### 3.2.2 Predators

The ground beetles *Amara* spp. are often known to visit flowers for feeding. The very abundant soldier beetle (*Rhagonycha fulva*) is frequently found in flowers of Apiaceae and Asteraceae, where it hunts smaller insects. Ladybirds (Coccinellidae) are some of the most characteristic insects on plants, where they hunt aphids, and two species (*Coccinella septempunctata *and *Harmonia axyridis*) were detected by eDNA in the study. In fact, eDNA from the invasive Asian lady beetle (*Harmonia axyridis*), which is a heavy predator on aphids (Koch, 2003), were detected on the same sample as eDNA from *Aphis* sp. were detected (Poll_10). We also detected eDNA from a number of spiders in the families Linyphiidae, Miturgidae, and Anyphaenidae.

#### 3.2.3 Gall inducers

Gall midges (Cecidomyiidae) were very abundant in the eDNA data. This family is very diverse, and several species could only be identified to Cecidomyiidae spp., possibly due to incomplete coverage in the database, and the fact that this family may be extraordinarily diverse based on molecular data from the DNA barcoding region (Hebert et al., 2016).

#### 3.2.4 Parasitoids

We recovered eDNA from two parasitic braconid wasps (Braconidae), which both use aphids as hosts (*Lysiphlebus hirticornis* and *Praon* sp.).

#### 3.2.5 Other phytophagous insects

Our study detected a number of insect taxa that feed on various parts of plants such as pollen, leaves, and sap. Thrips (Thysanoptera) were the most abundant taxon in the eDNA data. This group of insects can be very abundant in flowers, feeding on leaves, and pollen. Aphids (Aphididae) are often very numerous on flowering plants and were detected by eDNA from all plant species. We obtained eDNA from six aphid species in the study (Table S2). *Semiaphis dauci* uses *Daucus carotea* as a key host plant, and the large majority of eDNA reads were obtained from one sample of *Daucus*. *Euceraphis betulae* uses birch (*Betula pendula*) as host plant, which is very common at the sampling site. Several species of plant bugs (Miridae) were detected from eDNA in our study. As an example, we detected eDNA from two species of Orthops, *Orthops basalis* and *Orthops campestris* *, *known to live socially on Apiaceae flowers. The large majority of these reads were obtained from *Daucus* and *Angelica*, which are both members of Apiaceae. Other families of true bugs were also detected. The longhorn beetle *Leptura quadrifasciata* is frequently found on flowers of, for example, Apiaceae (such as *Daucus carota* in this study), where it feeds on pollen. The majority of eDNA reads from *Leptura quadrifasciata* were detected from *Daucus*, but it was also found on *Angelica* and *Eupatorium*.

### 3.3 Saturation and rarefaction analyses

**Figure 4:**
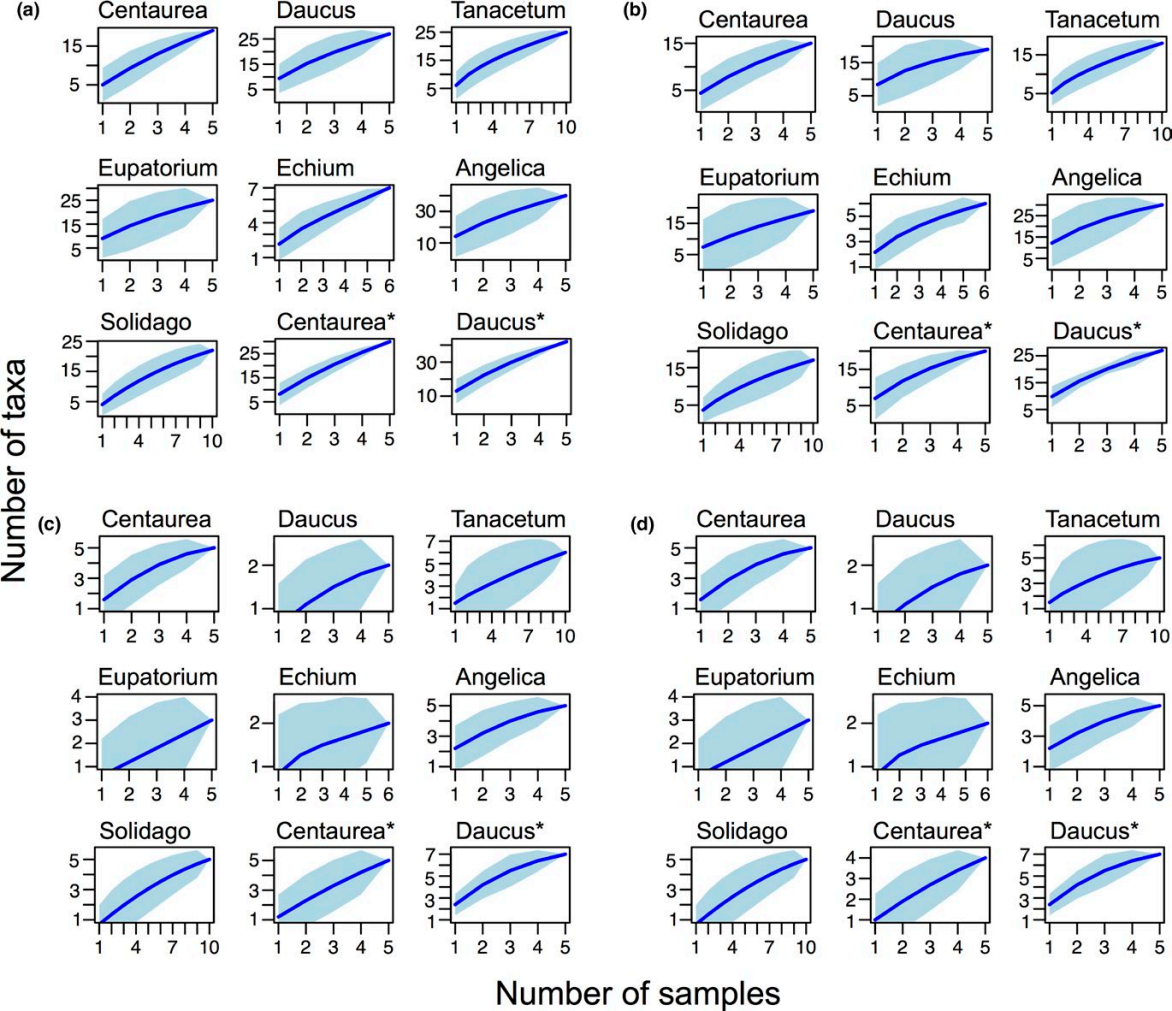
Accumulation curves for arthropods on each plant species. Accumulated expected mean species richness (blue line) and its standard deviation (turquoise area) with the number of samples analyzed. From the steepness of the curves, the analyses indicate that more taxa could be identified by including more samples. (a) COI species level, (b) COI family level, (c) 16S species level, (d) 16S family level. *Transect samples collected with 10 m distance between each

Accumulation curves for flower sample replicates of individual plant species indicated that greater sampling effort would probably increase recovered diversity (Figure 4). Similarly, there was generally a stepwise increase in number of taxa with the four PCR replicates (Supporting Information Figures S4–S5), indicating that more replicates would increase recovered richness. Rarefaction curves showed that the individual PCR replicates were sufficiently sequenced (Supporting Information Figures S6–S7).

### 3.4 Differences between the 16S and COI gene

Higher diversity was obtained for the COI gene than for the 16S gene (Supporting Information Table S2, Figures S2–S3). In fact, only seven unique families were obtained with 16S, and only 11 of the 67 families were recovered with both genes (Supporting Information Figure S2). Nevertheless, the two genes together covered a greater part of the arthropod diversity in the sampled flowers than each of them did in isolation. As an example, bees (Apidae) were only detected with 16S. In the mock sample, two of five species were recovered in COI, while were four of five were recovered for 16S (Table S5). Due to the incomplete 16S dataset, the subsequent analyses of the arthropod communities were performed on the COI dataset.

### 3.5 Differentiation of arthropod communities by plant species

**Figure 5:**
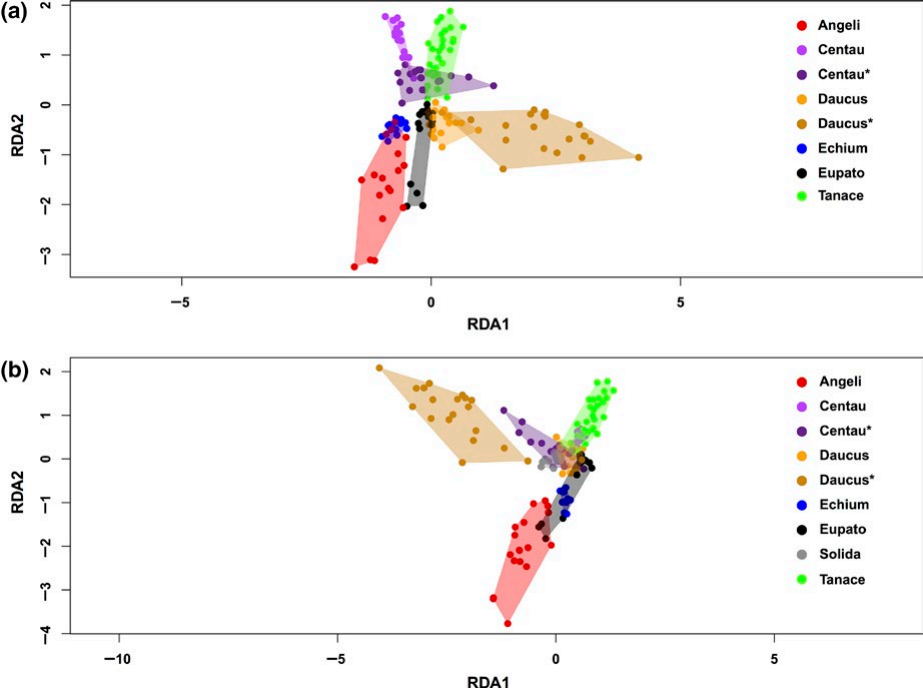
Redundancy analysis plot for COI, (a) Solidago not included, (b) Solidago included. Plant names: Angeli (*Angelica archangelica*), Centau (*Centaurea jacea*), Daucus (*Daucus carota*), Echium (*Echium vulgare*), Eupato (*Eupatorium cannabinum*), Solida (*Solidago canadensis*), Tanace (*Tanacetum vulgare*). *Transect samples collected with 10 m distance between each

**Figure 6:**
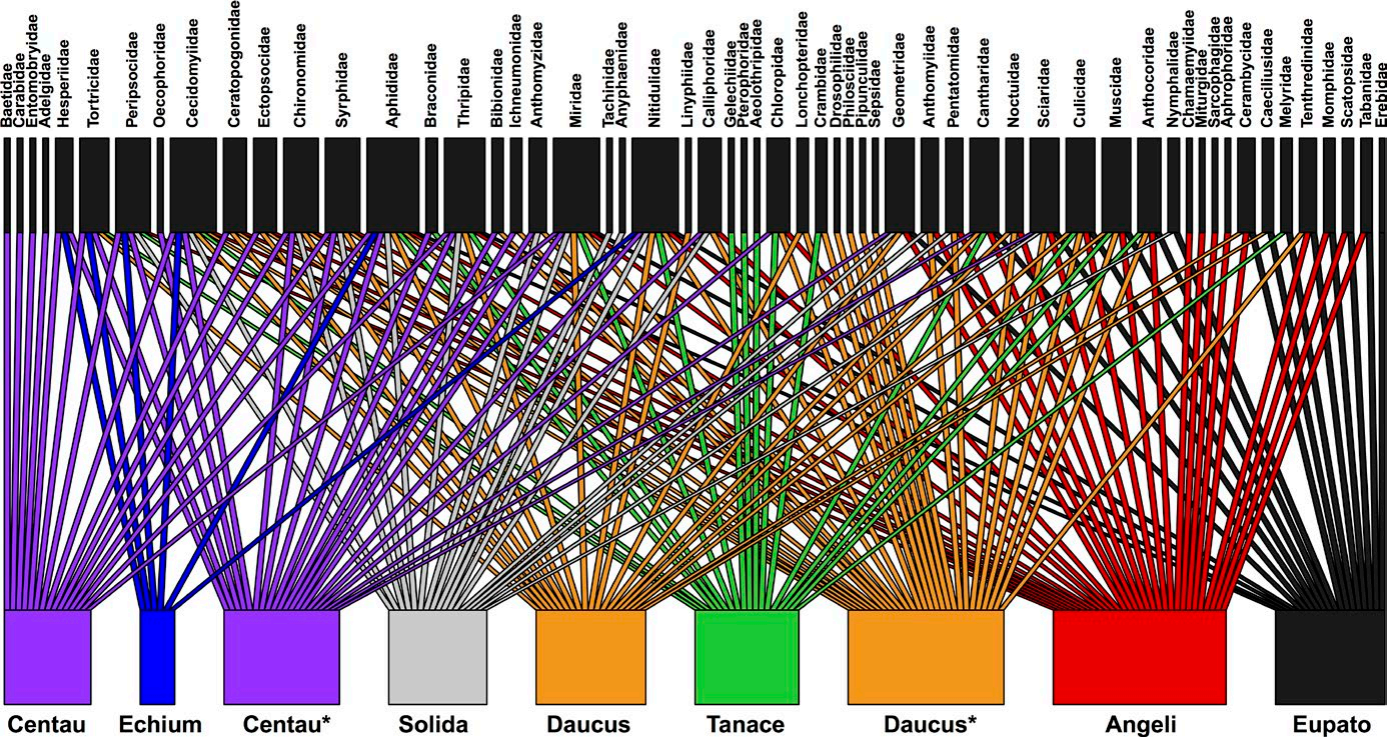
Bipartite plot for COI. The figure shows from which plants each arthropod family is obtained from. Plant names: Angeli (*Angelica archangelica*), Centau (*Centaurea jacea*), Daucus (*Daucus carota*), Echium (*Echium vulgare*), Eupato (*Eupatorium cannabinum*), Solida (*Solidago canadensis*), Tanace (*Tanacetum vulgare*). *Transect samples collected with 10 m distance between each

Results from the cluster analyses and RDA show that the communities of arthropods obtained from eDNA were somewhat, although not perfectly, segregated by plant species (Figure 5, Supporting Information Figure S8). The bipartite plot indicates that flower samples with large surface area such as Apiaceae umbels (*Angelica* and *Daucus*) contained the highest diversity, whereas samples collected as single flowers (*Echium*) had the lowest diversity (Figure 6). The arthropod families found in the most plants seemed to be abundant groups such as aphids, thrips, plant bugs, gall midges, and sap beetles (Nitidulidae).

### 3.6 Comparison of eDNA and faunistic records

Of the 135 taxa recovered from eDNA metabarcoding (COI and 16S combined), 93 taxa fulfilled the criteria that they (a) were obtained from Vestamager, (b) could be resolved to species (and in a few cases Genus) level, and (c) had at least one record in Naturbasen. Of these, 55 taxa (59%) have been recorded at the same sampling locality, and 68 taxa (73%) have been recorded in the island of Amager (Table S2). Two species obtained by eDNA are not previously recorded in Denmark, and for these, it was checked that reference sequences for all known Danish species in the particular genera were found in BOLD.

## 4 DISCUSSION

Terrestrial arthropods represent the majority of life on Earth (Mayhew, 2007; Stork, 2018), and many of them form complex associations with plants (Price et al., 2011). Numerous global research projects are providing new knowledge on these relationships (Bruce, 2015), and even the relatively species‐poor communities of Europe harbor thousands of species. The monitoring of species compositions and associations in a wild grassland habitat can thus be very demanding to study, and the challenge is exacerbated by the decline in taxonomic expertise. This necessitates the search for new methods to gain insights to the biological associations between arthropods and plants in terrestrial habitats.

The current study demonstrates that sequencing of eDNA from flowers can be a useful supplement to scientific experiments on terrestrial arthropods using traditional trapping methods, as well as for general biodiversity assessments. Our study uncovered eDNA from arthropods across many different taxonomic and functional groups. We obtained eDNA from pollinators (e.g., bees, butterflies, and hoverflies), predators (e.g., spiders and harvestmen), gall inducers (e.g., gall midges), parasitoids (e.g., braconid and ichneumonid wasps), and several phytophagous insects (e.g., weevils, true bugs, thrips etc.). Additionally, some species were most likely infrequent visitors (e.g., the mayfly *Cloeon dipterum*, the isopod *Philoscia muscorum*, and the earwig *Forficula auricularia*, although the latter is often found in flowers and on leaves). Such a non‐invasive approach could become useful for better estimation of species compositions and distributions, long‐term changes in abundance (Hallmann et al., 2017; Shortall et al., 2009), monitoring of endangered or invasive species, and for studies of insect fauna under environmental change (e.g., Thomsen, Jørgensen, et al., 2016). Also, the approach could be beneficial for documenting currently unknown plant–insect interactions for rare, cryptic or even undescribed insect species and for agricultural pest management. In the following, we discuss our results with increased focus on the limitations and future improvements of the current approach, which are essential to consider before it can be implemented to reach the above‐mentioned perspectives.

### 4.1 Differentiated arthropod communities

The arthropod communities recovered from eDNA clustered somewhat according to the plant species on which they were obtained. However, the separation was far from perfect as no plant species came out as a single monophyletic group of samples (Supporting Information Figure S8). The best clustering was seen for *Tanacetum*, *Echium* and *Angelica*, where all but one sample clustered together. Results from the RDA also showed some separation of communities according to the plant species, but again there were areas of overlap (Figure 5). Notably, even when *Centaurea* and *Daucus* flowers were sampled interspersed in a transect (Poll_49‐68: Centau* and Daucus*), they still seem to group somewhat by species, as well as with flowers of the same species sampled further away (Centau and Daucus) (Figure 5, Supporting Information Figure S8). The *Angelica* flowers yielded the highest number of arthropod taxa, while *Echium* displayed the lowest species richness (Supporting Information Figure S3). This is generally in accordance with the bipartite plot, where most families were obtained from Apiacae (*Angelica* and *Daucus*), while *Echium* had the lowest diversity (Figure 6). This could be explained by surface area of the flowers sampled, which simply allows contacts with more arthropods. However, these finding might not reflect the actual number of taxa that these flowers host, as the result could also be influenced by an incomplete coverage in sequencing and/or PCR replicates (Figure 4, Supporting Information Figures S4–S7).

### 4.2 Comparison of eDNA metabarcoding with faunistic records

Generally, we found very good concordance between faunistic records of occurrence and eDNA metabarcoding results. In total, 59% of the species obtained from eDNA in Vestamager are known to occur at the site. Considering that public citizen science data are far from complete, this is rather impressive. Thus, it is most likely that the incongruence between the two sources of occurrence data is partly due to incomplete investigations in the study area. Intriguingly, we found eDNA from the braconid wasp *Praon* sp. (100% match to both *P. longicorne* and *P. volucre*). *Praon* spp. are parasitoids on aphids, which were also found by eDNA in the study. Environmental DNA from *Praon* sp. was obtained in large read numbers from sample Poll_49, which also yielded many eDNA reads from the aphid *Hyalopterus pruni*—a known host species of *P. volucre* (Kavallieratos et al., 2005). This indicates that the current approach can potentially also be used to infer links between insects and their unknown host species.

### 4.3 Perspectives for pollination studies

The majority of insect pollination studies focus on bees, butterflies, and hoverflies (Ollerton, 2017). However, moths and flies are likely very underrepresented in pollination analyses. For example, moths are, by a large margin, the most diverse group of pollinators due to their specialized mouthparts (Ollerton, 2017; Wardhaugh, 2015), and the importance of non‐syrphid flies as pollinators is generally neglected (Orford, Vaughan, & Memmott, 2015). In crop pollination, the importance of non‐bees has been demonstrated and might even be more robust to changes in land use (Rader et al., 2016). A notable finding in our study is the diversity of families obtained from various insect groups (Figure 3, Table 1). In fact, the highest diversity (in families and species) was obtained from Diptera and Lepidoptera. Besides butterflies and hoverflies, we also obtained eDNA from several potentially important and understudied pollinators such as true flies (Muscidae), flower flies (Anthomyiidae), frit‐flies (Chloropidae), as well as several moth families such as geometer moths (Geometridae), and tortrix moths (Tortricidae). Primer bias toward certain insect groups must be taken into account (see section 4.5 below), but our results are promising for detection of flower‐visiting insect species.

Also, insects such as sap beetles and thrips, that are abundant in our eDNA sequencing data, are small in size and not conspicuously hairy, but can be extremely abundant and may thus contribute more to pollination than expected. Future studies would have to investigate the relative contribution of insect families to pollination and here, the flower eDNA approach may shed light on which families and species to focus on.

### 4.4 Other sources of arthropod eDNA on flowers

It is obvious that some of the arthropod eDNA obtained from flowers in this study could originate from other sources than actual eDNA left as traces from sloughed cells, fecal pellets etc. Some insect eggs, larvae, or very small imagoes may have been hidden in the flowers and escaped attention during sampling and DNA extraction. For example, given the relatively small size of thrips along with their abundance in the eDNA reads, we stress that the eDNA from this insect order might come from eggs or small juveniles hidden in the flowers. Also, several species of moths were detected only on their respective larval host plants—for instance, *Isophrictis striatella*, *Gillmeria ochrodactyla,* and *Dichrorampha obscuratana* were only detected on *Tanacetum vulgare*. This indicates that the eDNA might originate from eggs or perhaps from traces of larval activity. It has previously been shown that DNA can be obtained from empty leaf mines, although these must be assumed to contain little DNA (Derocles et al., 2015). However, these cases are considered the exception, and at least for the larger species, the eDNA must be assumed to originate from sloughed cells or fecal pellets left on the flowers. For spiders, eDNA may have originated from webs (Blake et al., 2016; Xu et al., 2015). Finally, arthropods visiting the flowers could potentially be carrying eDNA from other arthropod species originating from previous flower visits, which could be deposited on the flowers during subsequent visits. We assume that this is very infrequent compared to the source of eDNA deposited directly on the sampled flowers by the visiting species.

### 4.5 Choice of primers and database coverage in metabarcoding studies

The generic primers used in this study have been designed for metabarcoding of degraded DNA, and tested previously (Elbrecht et al., 2016; Zeale et al., 2011). Given the short amplicon size, they perform comparatively well by resolving most taxa to species level (Tables S2–S3). However, an inadequacy of the current approach is that the targeted region (for 16S) cannot resolve more groups to species level due to its low interspecific variation and incompleteness of the reference database compared with COI. Although new probabilistic methods for taxonomic assignments using 16S could improve the approach in future studies (Somervuo, Koskela, Pennanen, Henrik Nilsson, & Ovaskainen, 2016; Somervuo et al., 2017), identification to the species level is generally necessary for inferring relevant biological information on plant associations. Nevertheless, an impressive diversity of arthropods was still detected in the study. We chose two sets of primers in this study for increased taxonomic coverage (Alberdi et al., 2018) and because the two genes offer different advantages. Ribosomal genes are superior in metabarcoding studies due to the unbiased amplification of taxa within the target group (Deagle, Jarman, Coissac, Pompanon, & Taberlet, 2014), while increased taxonomic resolution is possible for the COI gene due to the extensive reference database. Meanwhile, the COI gene will most likely provide a biased representation of an eDNA sample, which was evident from the results of the mock sample that gave a much poorer representation of the actual sample content (Table S5).

Despite the more comprehensive coverage of arthropod diversity with the COI gene in this study, it is evident from accumulation analyses that even for COI, higher diversity can be obtained from flower samples than what we recovered here (Figure 4). Higher sequencing depth might saturate the number of taxa recovered, but also more PCR replicates on the same samples would likely increase the recovered diversity (Supporting Information Figures S4–S5). Some aquatic eDNA metabarcoding studies suggests running 12 PCR replicates of each sample in order to capture the rare sequences (Valentini et al., 2016), while a metabarcoding study on soil fungi argues that higher sequencing depth is more important for describing diversity than PCR replication (Smith & Peay, 2014).

The lack of database coverage for arthropods is a serious impediment for biodiversity studies using eDNA. The taxonomic identification of taxa is no better than the reference database used. Similar to other metabarcoding studies, the imperfect taxonomic identification of this study is thus due to the fact that (a) only an estimated 10% of arthropod species are described by science (less relevant for the study area in Denmark compared to other parts of the world), (b) not all described species have a DNA reference sequence for any of the two particular target fragments, and finally (c) the primers used here cannot positively discriminate all species, as mentioned above. The database issue is however continuously being abated as more reference sequences are generated.

### 4.6 Future focus and validation

While the current flower–arthropod eDNA approach provides great perspectives for both fundamental and applied research on plant‐associated arthropod communities, a range of uncertainties should be addressed to fully validate the perspectives. In the following, we thus suggest several experiments for future studies in order to obtain better insights into the nature of arthropod eDNA on flowers. Firstly, a comparative study of actual trapping experiments in parallel with eDNA sampling would further validate the correspondence between eDNA reads and insect richness and abundance from traps. In the current study, we indirectly validated our results through Danish citizen science occurrence data (naturbasen.dk), but these data are incomplete and do not reflect the actual occurrence or abundance of arthropods in the particular time of sampling. Especially, the quantitative aspect of abundance would be relevant to investigate, as eDNA from other sources such as soil and water samples suggest that relative abundance estimates are to some degree possible (Andersen et al., 2012; Thomsen et al., 2012; Thomsen, Møller, et al., 2016). Also, the degradation of eDNA on the flowers should be investigated. The seasonal and diurnal variation of eDNA on flowers, and how this relates to actual visitation by insects, which is indirectly related to the degradation time, should also be explored. Seasonal signals have been observed in studies of aquatic eDNA from nonbiting midges (Chironomidae) (Bista et al., 2017), as well as from marine fishes (Sigsgaard et al., 2017; Stoeckle, Soboleva, & Charlop‐Powers, 2017). Another question to test through experiments would be to investigate to what extent certain insects leave more eDNA on the flowers than others, and whether this is associated with more frequent and/or longer visits to the flowers. Additionally, the origin of arthropod DNA on the flowers would be very relevant to explore. For example, do insects carry eDNA from other insects with them between flowers (as mentioned above)? If this is the case, it would lead to eDNA detection of insects that had no contact with the flower, and could lead to false positive results on insect–plant associations and occurrences. Furthermore, a general improvement of methodological approaches would be relevant. It has been shown that complete mitochondrial genomes can be extracted from water samples (Deiner et al., 2017)—such an approach would greatly improve the taxonomic identification and subsequent ecological inferences in studies such as this one (Crampton‐Platt, Yu, Zhou, & Vogler, 2016). We believe the DNA extraction procedure on arthropod eDNA from flowers could also be made more efficient through comparative experiments, which has been made for eDNA in water samples (Deiner, Walser, Mächler, & Altermatt, 2015).

Finally, we encourage a general replication of the approach, which should also include other types of habitats and other families of plants, in order to test the reproducibility of the method.

## CONFLICT OF INTEREST

None declared.

## AUTHOR CONTRIBUTIONS

PFT conceived the idea and designed the methodology; PFT collected the samples; PFT and EES performed the laboratory work; PFT and EES analyzed the data; PFT and EES wrote the manuscript.

## Data Availability

Illumina NextSeq raw sequence data are available from the Dryad Digital Repository (https://doi.org/10.5061/dryad.2j151bd).
