# Supplementary material for: Environmental DNA metabarcoding of wild flowers reveals diverse communities of terrestrial arthropods
Source: Ecol Evol. 2019 Feb 7;9(4):1665–79. doi: 10.1002/ece3.4809 (PMC6392377; doi:10.1002/ece3.4809)
Supplement: Supplementary file 1 [file ECE3-9-1665-s001.docx]

**SUPPORTING INFORMATION**

for

**Environmental DNA metabarcoding of wild flowers reveals diverse communities of terrestrial arthropods**

Philip Francis Thomsen*, Eva Egelyng Sigsgaard

Department of Bioscience, University of Aarhus, Ny Munkegade 116, DK-8000 Aarhus C, Denmark.

*Correspondence author. E-mail: pfthomsen@bios.au.dk

**Table S1**. Overview of samples, GPS coordinates, parts of the plant sampled and DNA extraction information.

| **Sample name** | **No. of samples** | **Short plant name** | **Plant species** | **Lat.** | **Long.** | **Part sampled** | **Input ATL/ul** | **Input prot. K/ul** |
| --- | --- | --- | --- | --- | --- | --- | --- | --- |
| **Poll_01-06** | **6** | **Centau** | ***Centaurea jacea*** | **55,599308** | **12,5734841381709** | **1 head** | **900** | **100** |
| **Poll_10-13, 34** | **5** | **Daucus** | ***Daucus carotea*** | **55,596236** | **12,5563711474251** | **1 umbell** | **1800** | **200** |
| **Poll_14-18** | **5** | **Tanace** | ***Tanacetum vulgare*** | **55,594559** | **12,5606342418961** | **5 heads** | **1800** | **200** |
| **Poll_19-23** | **5** | **Tanace** | ***Tanacetum vulgare*** | **55,597020** | **12,5601004761393** | **5 heads** | **1800** | **200** |
| **Poll_24-28** | **5** | **Eupato** | ***Eupatorium cannabinum*** | **55,596587** | **12,5595717623930** | **several heads** | **1800** | **200** |
| **Poll_38-43** | **6** | **Echium** | ***Echium vulgare*** | **55,607067** | **12,5622393270987** | **1 flower** | **540** | **60** |
| **Poll_44-48** | **5** | **Angeli** | ***Angelica archangelica*** | **55,593174** | **12,5714660848952** | **1 umbell** | **1800** | **200** |
| **Poll_49-68start** | **10** | **Centau*, Daucus*** | ***Centaurea jacea, Daucus carotea*** | **55,600447** | **12,5701930000000** | **as above** | **as above** | **as above** |
| **Poll_49-68end** | **NA** | **Centau*, Daucus*** | ***Centaurea jacea, Daucus carotea*** | **55,601027** | **12,5722031939625** | **as above** | **NA** | **NA** |
| **Poll_69-78** | **10** | **Solida** | ***Solidago canadensis*** | **55,417490** | **11,5892630000000** | **several heads** | **1800** | **200** |

**Table S2**. Final list of reads from arthropod species identified to min. family level, and obtained from min. 2 PCR replicates. Table includes genes from which the family is obtained and sites of Danish occurrences (data from Naturbasen). ^) The match to *Asteromyia* sp. should be considered unverified since this is a Nearctic genus (Gagné & Jaschhof, 2017). See Table S3 for more details on identifications.

| **Class** | **Order** | **Family** | **Genus** | **Species** | **COI** | **16S** | **Vestamager** | **Amager** | **Copenhagen** | **Zealand** | **Rest of Denmark** | **notes** |
| --- | --- | --- | --- | --- | --- | --- | --- | --- | --- | --- | --- | --- |
| **Arachnida** | **Araneae** | **Anyphaenidae** | **Anyphaena** | **Anyphaena accentuata** | **1** |  |  |  |  |  |  |  |
| **Arachnida** | **Araneae** | **Linyphiidae** | **Neriene** | **Neriene clathrata** | **1** |  |  |  | **1** |  |  |  |
| **Arachnida** | **Araneae** | **Miturgidae** | **Cheiracanthium** | **Cheiracanthium sp.** | **1** |  |  |  | **1** |  |  |  |
| **Arachnida** | **Opiliones** | **Leiobunidae** | **Leiobunum** | **Leiobunum rotundum** |  | **1** | **1** | **1** |  |  |  |  |
| **Branchiopoda** | **Diplostraca** | **Polyphemidae** | **Polyphemus** | **Polyphemus pediculus** |  | **1** | **no data** | **no data** | **no data** | **no data** | **no data** | **no data** |
| **Collembola** | **Entomobryomorpha** | **Entomobryidae** | **Entomobrya** | **Entomobrya sp.** | **1** |  |  | **1** |  |  |  |  |
| **Collembola** | **Entomobryomorpha** | **Entomobryidae** | **Willowsia** | **Willowsia nigromaculata** |  | **1** |  |  | **1** |  |  |  |
| **Collembola** | **Entomobryomorpha** | **Isotomidae** | **Isotoma** | **Isotoma viridis** | **1** | **1** |  | **1** |  |  |  |  |
| **Insecta** | **Coleoptera** | **Brentidae** | **Apion** | **Apion fulvipes** | **1** | **1** | **1** | **1** |  |  |  |  |
| **Insecta** | **Coleoptera** | **Cantharidae** | **Rhagonycha** | **Rhagonycha fulva** | **1** |  | **1** | **1** |  |  |  |  |
| **Insecta** | **Coleoptera** | **Carabidae** | **Amara** | **Amara similata** | **1** |  |  |  | **1** |  |  |  |
| **Insecta** | **Coleoptera** | **Carabidae** | **Carabus** | **Carabus nemoralis** | **1** | **1** | **1** | **1** |  |  |  |  |
| **Insecta** | **Coleoptera** | **Cerambycidae** | **Leptura** | **Leptura quadrifasciata** | **1** |  | **1** | **1** |  |  |  |  |
| **Insecta** | **Coleoptera** | **Coccinellidae** | **Coccinella** | **Coccinella septempunctata** |  | **1** | **1** | **1** |  |  |  |  |
| **Insecta** | **Coleoptera** | **Coccinellidae** | **Harmonia** | **Harmonia axyridis** |  | **1** | **1** | **1** |  |  |  |  |
| **Insecta** | **Coleoptera** | **Melyridae** | **Dasytes** | **Dasytes plumbeus** | **1** |  | **1** | **1** |  |  |  |  |
| **Insecta** | **Coleoptera** | **Nitidulidae** | **Meligethes** | **Meligethes aeneus** | **1** | **1** | **1** | **1** |  |  |  |  |
| **Insecta** | **Coleoptera** | **Nitidulidae** | **Meligethes** | **Meligethes planiusculus** | **1** | **1** |  |  | **1** |  |  |  |
| **Insecta** | **Dermaptera** | **Forficulidae** | **Forficula** | **Forficula auricularia** |  | **1** | **1** | **1** |  |  |  |  |
| **Insecta** | **Diptera** | **Anthomyiidae** | **Botanophila** | **Botanophila fugax** | **1** |  | **1** | **1** |  |  |  |  |
| **Insecta** | **Diptera** | **Anthomyiidae** | **Delia** | **Delia platura** | **1** |  | **1** | **1** |  |  |  |  |
| **Insecta** | **Diptera** | **Anthomyiidae** | **Delia** | **Delia radicum** | **1** |  |  |  |  | **1** |  |  |
| **Insecta** | **Diptera** | **Anthomyzidae** | **Anthomyza** | **Anthomyza collini** | **1** |  | **1** | **1** |  |  |  |  |
| **Insecta** | **Diptera** | **Anthomyzidae** | **Anthomyza** | **Anthomyza gracilis** | **1** |  |  |  |  |  |  |  |
| **Insecta** | **Diptera** | **Bibionidae** | **Dilophus** | **Dilophus febrilis** | **1** |  | **1** | **1** |  |  |  |  |
| **Insecta** | **Diptera** | **Calliphoridae** | **Angioneura** | **Angioneura acerba** | **1** |  | **1** | **1** |  |  |  |  |
| **Insecta** | **Diptera** | **Calliphoridae** | **Lucilia** | **Lucilia caesar** | **1** | **1** |  |  |  |  |  |  |
| **Insecta** | **Diptera** | **Calliphoridae** | **Lucilia** | **Lucilia sp.** | **1** |  | **1** | **1** |  |  |  |  |
| **Insecta** | **Diptera** | **Calliphoridae** | **Pollenia** | **Pollenia rudis** |  | **1** | **1** | **1** |  |  |  |  |
| **Insecta** | **Diptera** | **Cecidomyiidae** | **Asteromyia** | **Asteromyia sp.^** | **1** |  | **NA** | **NA** | **NA** | **NA** | **NA** |  |
| **Insecta** | **Diptera** | **Cecidomyiidae** | **NA** | **Cecidomyiidae sp.1** | **1** |  | **NA** | **NA** | **NA** | **NA** | **NA** |  |
| **Insecta** | **Diptera** | **Cecidomyiidae** | **NA** | **Cecidomyiidae sp.2** | **1** |  | **NA** | **NA** | **NA** | **NA** | **NA** |  |
| **Insecta** | **Diptera** | **Cecidomyiidae** | **NA** | **Cecidomyiidae sp.3** | **1** |  | **NA** | **NA** | **NA** | **NA** | **NA** |  |
| **Insecta** | **Diptera** | **Cecidomyiidae** | **NA** | **Cecidomyiidae sp.4** | **1** |  | **NA** | **NA** | **NA** | **NA** | **NA** |  |
| **Insecta** | **Diptera** | **Cecidomyiidae** | **NA** | **Cecidomyiidae sp.5** | **1** |  | **NA** | **NA** | **NA** | **NA** | **NA** |  |
| **Insecta** | **Diptera** | **Cecidomyiidae** | **NA** | **Cecidomyiidae sp.7** | **1** |  | **NA** | **NA** | **NA** | **NA** | **NA** |  |
| **Insecta** | **Diptera** | **Cecidomyiidae** | **NA** | **Cecidomyiidae sp.8** | **1** |  | **NA** | **NA** | **NA** | **NA** | **NA** |  |
| **Insecta** | **Diptera** | **Cecidomyiidae** | **Ozirhincus** | **Ozirhincus longicollis** | **1** |  |  | **1** |  |  |  |  |
| **Insecta** | **Diptera** | **Cecidomyiidae** | **Peromyia** | **Peromyia sp.** | **1** |  | **no data** | **no data** | **no data** | **no data** | **no data** | **no data** |
| **Insecta** | **Diptera** | **Cecidomyiidae** | **Rhopalomyia** | **Rhopalomyia sp.** | **1** |  | **1** | **1** |  |  |  |  |
| **Insecta** | **Diptera** | **Ceratopogonidae** | **Culicoides** | **Culicoides impunctatus** | **1** |  | **no data** | **no data** | **no data** | **no data** | **no data** | **no data** |
| **Insecta** | **Diptera** | **Ceratopogonidae** | **Dasyhelea** | **Dasyhelea sp.** | **1** |  | **no data** | **no data** | **no data** | **no data** | **no data** | **no data** |
| **Insecta** | **Diptera** | **Chamaemyiidae** | **Leucopis** | **Leucopis sp.** | **1** |  |  |  | **1** |  |  |  |
| **Insecta** | **Diptera** | **Chironomidae** | **Bryophaenocladius** | **Bryophaenocladius sp.** | **1** |  | **no data** | **no data** | **no data** | **no data** | **no data** | **no data** |
| **Insecta** | **Diptera** | **Chironomidae** | **Cricotopus** | **Cricotopus ornatus** | **1** |  | **no data** | **no data** | **no data** | **no data** | **no data** | **no data** |
| **Insecta** | **Diptera** | **Chironomidae** | **Glyptotendipes** | **Glyptotendipes sp.** | **1** |  | **no data** | **no data** | **no data** | **no data** | **no data** | **no data** |
| **Insecta** | **Diptera** | **Chironomidae** | **Guttipelopia** | **Guttipelopia guttipennis** | **1** |  | **no data** | **no data** | **no data** | **no data** | **no data** | **no data** |
| **Insecta** | **Diptera** | **Chironomidae** | **Halocladius** | **Halocladius variabilis** | **1** |  | **no data** | **no data** | **no data** | **no data** | **no data** | **no data** |
| **Insecta** | **Diptera** | **Chironomidae** | **Halocladius** | **Halocladius varians** | **1** |  | **no data** | **no data** | **no data** | **no data** | **no data** | **no data** |
| **Insecta** | **Diptera** | **Chironomidae** | **Metriocnemus** | **Metriocnemus fuscipes** | **1** |  | **no data** | **no data** | **no data** | **no data** | **no data** | **no data** |
| **Insecta** | **Diptera** | **Chloropidae** | **Elachiptera** | **Elachiptera cornuta** | **1** |  | **1** | **1** |  |  |  |  |
| **Insecta** | **Diptera** | **Chloropidae** | **Meromyza** | **Meromyza sp.** | **1** |  |  |  | **1** |  |  |  |
| **Insecta** | **Diptera** | **Chloropidae** | **Oscinella** | **Oscinella sp.** | **1** |  | **1** | **1** |  |  |  |  |
| **Insecta** | **Diptera** | **Chloropidae** | **Siphonella** | **Siphonella oscinina** | **1** |  |  |  |  |  | **1** |  |
| **Insecta** | **Diptera** | **Culicidae** | **Culex** | **Culex sp.** |  | **1** | **1** | **1** |  |  |  |  |
| **Insecta** | **Diptera** | **Culicidae** | **Ochlerotatus** | **Ochlerotatus detritus** | **1** |  | **no data** | **no data** | **no data** | **no data** | **no data** | **no data** |
| **Insecta** | **Diptera** | **Drosophilidae** | **Drosophila** | **Drosophila fenestrarum** | **1** |  |  |  | **1** |  |  |  |
| **Insecta** | **Diptera** | **Lonchopteridae** | **Lonchoptera** | **Lonchoptera bifurcata** | **1** |  | **1** | **1** |  |  |  |  |
| **Insecta** | **Diptera** | **Muscidae** | **Coenosia** | **Coenosia tigrina** | **1** |  | **1** | **1** |  |  |  |  |
| **Insecta** | **Diptera** | **Muscidae** | **Morellia** | **Morellia aenescens** | **1** |  | **1** | **1** |  |  |  |  |
| **Insecta** | **Diptera** | **Muscidae** | **Morellia** | **Morellia hortorum** | **1** |  | **1** | **1** |  |  |  |  |
| **Insecta** | **Diptera** | **Muscidae** | **Musca** | **Musca autumnalis** | **1** | **1** | **1** | **1** |  |  |  |  |
| **Insecta** | **Diptera** | **Muscidae** | **Neomyia** | **Neomyia cornicina** | **1** |  | **1** | **1** |  |  |  |  |
| **Insecta** | **Diptera** | **Opomyzidae** | **Opomyza** | **Opomyza florum** |  | **1** | **1** | **1** |  |  |  |  |
| **Insecta** | **Diptera** | **Pipunculidae** | **Cephalops** | **Cephalops semifumosus** | **1** |  | **no data** | **no data** | **no data** | **no data** | **no data** | **no data** |
| **Insecta** | **Diptera** | **Sarcophagidae** | **Macronychia** | **Macronychia polyodon** | **1** |  | **1** | **1** |  |  |  |  |
| **Insecta** | **Diptera** | **Scatopsidae** | **Coboldia** | **Coboldia fuscipes** | **1** |  | **no data** | **no data** | **no data** | **no data** | **no data** | **no data** |
| **Insecta** | **Diptera** | **Sciaridae** | **Bradysia** | **Bradysia nitidicollis** | **1** |  | **no data** | **no data** | **no data** | **no data** | **no data** | **no data** |
| **Insecta** | **Diptera** | **Sciaridae** | **Hyperlasion** | **Hyperlasion wasmanni** | **1** |  | **no data** | **no data** | **no data** | **no data** | **no data** | **no data** |
| **Insecta** | **Diptera** | **Sciaridae** | **Schwenckfeldina** | **Schwenckfeldina carbonaria** | **1** | **1** |  |  |  |  |  |  |
| **Insecta** | **Diptera** | **Sepsidae** | **Sepsis** | **Sepsis cynipsea** | **1** |  | **1** | **1** |  |  |  |  |
| **Insecta** | **Diptera** | **Syrphidae** | **Eristalis** | **Eristalis pertinax** | **1** |  | **1** | **1** |  |  |  |  |
| **Insecta** | **Diptera** | **Syrphidae** | **Platycheirus** | **Platycheirus clypeatus** | **1** |  | **1** | **1** |  |  |  |  |
| **Insecta** | **Diptera** | **Syrphidae** | **Sphaerophoria** | **Sphaerophoria sp.** | **1** |  | **1** | **1** |  |  |  |  |
| **Insecta** | **Diptera** | **Syrphidae** | **Syrphus** | **Syrphus vitripennis** | **1** |  | **1** | **1** |  |  |  |  |
| **Insecta** | **Diptera** | **Tabanidae** | **Haematopota** | **Haematopota sp.** | **1** |  | **1** | **1** |  |  |  |  |
| **Insecta** | **Diptera** | **Tachinidae** | **Dinera** | **Dinera ferina** | **1** |  |  |  |  |  |  |  |
| **Insecta** | **Diptera** | **Tachinidae** | **Phasia** | **Phasia hemiptera** | **1** |  |  |  |  |  |  |  |
| **Insecta** | **Ephemeroptera** | **Baetidae** | **Cloeon** | **Cloeon dipterum** | **1** | **1** |  |  | **1** |  |  |  |
| **Insecta** | **Hemiptera** | **Adelgidae** | **Pineus** | **Pineus sp.** | **1** |  | **no data** | **no data** | **no data** | **no data** | **no data** | **no data** |
| **Insecta** | **Hemiptera** | **Anthocoridae** | **Orius** | **Orius laticollis** | **1** |  | **no data** | **no data** | **no data** | **no data** | **no data** | **no data** |
| **Insecta** | **Hemiptera** | **Anthocoridae** | **Orius** | **Orius niger** | **1** |  |  |  | **1** |  |  |  |
| **Insecta** | **Hemiptera** | **Anthocoridae** | **Orius** | **Orius sp.** | **1** |  |  |  | **1** |  |  |  |
| **Insecta** | **Hemiptera** | **Aphididae** | **Aphis** | **Aphis sp.** | **1** |  | **1** | **1** |  |  |  |  |
| **Insecta** | **Hemiptera** | **Aphididae** | **Euceraphis** | **Euceraphis betulae** | **1** |  |  |  | **1** |  |  |  |
| **Insecta** | **Hemiptera** | **Aphididae** | **Hyadaphis** | **Hyadaphis foeniculi** | **1** |  |  |  | **1** |  |  |  |
| **Insecta** | **Hemiptera** | **Aphididae** | **Hyalopterus** | **Hyalopterus pruni** | **1** |  | **1** | **1** |  |  |  |  |
| **Insecta** | **Hemiptera** | **Aphididae** | **Phyllaphis** | **Phyllaphis fagi** | **1** |  |  | **1** |  |  |  |  |
| **Insecta** | **Hemiptera** | **Aphididae** | **Semiaphis** | **Semiaphis dauci** | **1** |  | **no data** | **no data** | **no data** | **no data** | **no data** | **no data** |
| **Insecta** | **Hemiptera** | **Aphrophoridae** | **Philaenus** | **Philaenus spumarius** | **1** |  | **1** | **1** |  |  |  |  |
| **Insecta** | **Hemiptera** | **Miridae** | **Adelphocoris** | **Adelphocoris lineolatus** |  | **1** | **1** | **1** |  |  |  |  |
| **Insecta** | **Hemiptera** | **Miridae** | **Lygus** | **Lygus rugulipennis** | **1** |  |  | **1** |  |  |  |  |
| **Insecta** | **Hemiptera** | **Miridae** | **NA** | **Miridae sp.1** |  | **1** | **NA** | **NA** | **NA** | **NA** | **NA** |  |
| **Insecta** | **Hemiptera** | **Miridae** | **NA** | **Miridae sp.2** |  | **1** | **NA** | **NA** | **NA** | **NA** | **NA** |  |
| **Insecta** | **Hemiptera** | **Miridae** | **Neolygus** | **Neolygus sp.** | **1** |  | **1** | **1** |  |  |  |  |
| **Insecta** | **Hemiptera** | **Miridae** | **Orthops** | **Orthops basalis** | **1** |  |  |  | **1** |  |  |  |
| **Insecta** | **Hemiptera** | **Miridae** | **Orthops** | **Orthops campestris** | **1** |  |  | **1** |  |  |  |  |
| **Insecta** | **Hemiptera** | **Miridae** | **Phytocoris** | **Phytocoris varipes** | **1** |  | **1** | **1** |  |  |  |  |
| **Insecta** | **Hemiptera** | **Pentatomidae** | **Palomena** | **Palomena prasina** | **1** |  | **1** | **1** |  |  |  |  |
| **Insecta** | **Hemiptera** | **Veliidae** | **Microvelia** | **Microvelia reticulata** |  | **1** |  |  | **1** |  |  |  |
| **Insecta** | **Hymenoptera** | **Apidae** | **Bombus** | **Bombus lapidarius** |  | **1** | **1** | **1** |  |  |  |  |
| **Insecta** | **Hymenoptera** | **Braconidae** | **Lysiphlebus** | **Lysiphlebus hirticornis** | **1** |  | **no data** | **no data** | **no data** | **no data** | **no data** | **no data** |
| **Insecta** | **Hymenoptera** | **Braconidae** | **Praon** | **Praon sp.** | **1** |  | **no data** | **no data** | **no data** | **no data** | **no data** | **no data** |
| **Insecta** | **Hymenoptera** | **Ichneumonidae** | **Promethes** | **Promethes sulcator** | **1** |  | **no data** | **no data** | **no data** | **no data** | **no data** | **no data** |
| **Insecta** | **Hymenoptera** | **Tenthredinidae** | **Athalia** | **Athalia rosae** | **1** |  |  | **1** |  |  |  |  |
| **Insecta** | **Hymenoptera** | **Tenthredinidae** | **Tenthredo** | **Tenthredo sp.** | **1** |  | **1** | **1** |  |  |  |  |
| **Insecta** | **Lepidoptera** | **Crambidae** | **Agriphila** | **Agriphila sp.** | **1** |  | **1** | **1** |  |  |  |  |
| **Insecta** | **Lepidoptera** | **Crambidae** | **Pleuroptya** | **Pleuroptya ruralis** | **1** |  | **1** | **1** |  |  |  |  |
| **Insecta** | **Lepidoptera** | **Erebidae** | **Eilema** | **Eilema griseola** | **1** |  |  |  | **1** |  |  |  |
| **Insecta** | **Lepidoptera** | **Gelechiidae** | **Isophrictis** | **Isophrictis striatella** | **1** |  |  |  |  | **1** |  |  |
| **Insecta** | **Lepidoptera** | **Geometridae** | **Acasis** | **Acasis viretata** | **1** |  |  | **1** |  |  |  |  |
| **Insecta** | **Lepidoptera** | **Geometridae** | **Chloroclystis** | **Chloroclystis v-ata** | **1** |  |  | **1** |  |  |  |  |
| **Insecta** | **Lepidoptera** | **Geometridae** | **Eupithecia** | **Eupithecia absinthiata** | **1** |  |  | **1** |  |  |  |  |
| **Insecta** | **Lepidoptera** | **Geometridae** | **Eupithecia** | **Eupithecia tripunctaria** | **1** |  |  |  | **1** |  |  |  |
| **Insecta** | **Lepidoptera** | **Geometridae** | **Eupithecia** | **Eupithecia virgaureata** | **1** |  |  |  |  |  |  |  |
| **Insecta** | **Lepidoptera** | **Geometridae** | **Scotopteryx** | **Scotopteryx chenopodiata** | **1** |  | **1** | **1** |  |  |  |  |
| **Insecta** | **Lepidoptera** | **Hesperiidae** | **Thymelicus** | **Thymelicus lineola** | **1** |  | **1** | **1** |  |  |  |  |
| **Insecta** | **Lepidoptera** | **Momphidae** | **Mompha** | **Mompha epilobiella** | **1** |  |  |  | **1** |  |  |  |
| **Insecta** | **Lepidoptera** | **Noctuidae** | **Apamea** | **Apamea anceps** | **1** |  |  | **1** |  |  |  |  |
| **Insecta** | **Lepidoptera** | **Noctuidae** | **Autographa** | **Autographa gamma** | **1** |  | **1** | **1** |  |  |  |  |
| **Insecta** | **Lepidoptera** | **Noctuidae** | **Mythimna** | **Mythimna sp.** | **1** |  | **1** | **1** |  |  |  |  |
| **Insecta** | **Lepidoptera** | **Nymphalidae** | **Aphantopus** | **Aphantopus hyperantus** | **1** |  | **1** | **1** |  |  |  |  |
| **Insecta** | **Lepidoptera** | **Oecophoridae** | **Hofmannophila** | **Hofmannophila pseudospretella** | **1** |  |  | **1** |  |  |  |  |
| **Insecta** | **Lepidoptera** | **Pterophoridae** | **Gillmeria** | **Gillmeria ochrodactyla** | **1** |  | **1** | **1** |  |  |  |  |
| **Insecta** | **Lepidoptera** | **Tortricidae** | **Dichrorampha** | **Dichrorampha obscuratana** | **1** |  | **no data** | **no data** | **no data** | **no data** | **no data** | **no data** |
| **Insecta** | **Lepidoptera** | **Tortricidae** | **Eucosma** | **Eucosma cana** | **1** |  |  |  | **1** |  |  |  |
| **Insecta** | **Lepidoptera** | **Tortricidae** | **Eucosma** | **Eucosma hohenwartiana/fulvana/parvulana complex** | **1** |  |  |  | **1** |  |  |  |
| **Insecta** | **Psocoptera** | **Caeciliusidae** | **Valenzuela** | **Valenzuela flavidus** | **1** |  | **1** | **1** |  |  |  |  |
| **Insecta** | **Psocoptera** | **Ectopsocidae** | **Ectopsocus** | **Ectopsocus briggsi** | **1** |  |  |  | **1** |  |  |  |
| **Insecta** | **Psocoptera** | **Peripsocidae** | **Peripsocus** | **Peripsocus subfasciatus** | **1** |  |  |  | **1** |  |  |  |
| **Insecta** | **Thysanoptera** | **Aeolothripidae** | **Aeolothrips** | **Aeolothrips fasciatus** | **1** |  |  | **1** |  |  |  |  |
| **Insecta** | **Thysanoptera** | **Thripidae** | **NA** | **Thripidae sp.** |  | **1** | **NA** | **NA** | **NA** | **NA** | **NA** |  |
| **Insecta** | **Thysanoptera** | **Thripidae** | **Thrips** | **Thrips major** | **1** |  | **no data** | **no data** | **no data** | **no data** | **no data** | **no data** |
| **Malacostraca** | **Isopoda** | **Asellidae** | **Asellus** | **Asellus aquaticus** | **1** | **1** | **1** | **1** |  |  |  |  |
| **Malacostraca** | **Isopoda** | **Philosciidae** | **Philoscia** | **Philoscia muscorum** | **1** |  | **1** | **1** |  |  |  |  |
| **TOTAL** |  |  |  |  | **120** | **25** | **55** | **68** | **22** | **2** | **1** | **93** |
| **% occurence** |  |  |  |  |  |  | **59** | **73** | **24** | **2** | **1** | **100** |

**Table S3**. List of all taxa identified including species obtained from only one PCR replicate with notes on identification. For 16S the identification notes are alternative taxonomic ID with match percentages. Taxa in blue denotes the additional taxa to the final list (Table S2) and is obtained from only a single PCR replicate. Taxa in red denotes obvious contaminants. *) Final taxonomic level of identification is adjusted based on similar matches. DK*) Final taxa ID is determined based on presence in Denmark. Data is given for COI and 16S separately. ^) The match to *Asteromyia* sp. should be considered unverified since this is a Nearctic genus (Gagné & Jaschhof, 2017).

| **COI** | | | | | | |
| --- | --- | --- | --- | --- | --- | --- |
| **Class** | **Order** | **Family** | **Genus** | **Species** | | **Identification notes** |
| **Arachnida** | **Araneae** | **Anyphaenidae** | **Anyphaena** | **Anyphaena accentuata** | |  |
| **Arachnida** | **Araneae** | **Araneidae** | **Araneus** | **Araneus quadratus** | |  |
| **Arachnida** | **Araneae** | **Clubionidae** | **Clubiona** | **Clubiona comta** | |  |
| **Arachnida** | **Araneae** | **Clubionidae** | **Clubiona** | **Clubiona phragmitis** | |  |
| **Arachnida** | **Araneae** | **Linyphiidae** | **Diplocephalus** | **Diplocephalus cristatus** | |  |
| **Arachnida** | **Araneae** | **Linyphiidae** | **Neriene** | **Neriene clathrata** | |  |
| **Arachnida** | **Araneae** | **Miturgidae** | **Cheiracanthium** | **Cheiracanthium sp.*** | | **Cheiracanthium pennyi, Cheiracanthium erraticum, Cheiracanthium campestre** |
| **Arachnida** | **Oribatida** | **Ceratozetidae** | **NA** | **Ceratozetidae sp.** | | **99% match** |
| **Arachnida** | **Oribatida** | **Punctoribatidae** | **Punctoribates** | **Punctoribates punctum** | |  |
| **Collembola** | **Entomobryomorpha** | **Entomobryidae** | **Entomobrya** | **Entomobrya sp.** | | **99% match** |
| **Collembola** | **Entomobryomorpha** | **Isotomidae** | **Isotoma** | **Isotoma sp.** | | **99% match** |
| **Insecta** | **Coleoptera** | **Brentidae** | **Apion** | **Apion apricans** | |  |
| **Insecta** | **Coleoptera** | **Brentidae** | **Apion** | **Apion fulvipes** | |  |
| **Insecta** | **Coleoptera** | **Brentidae** | **Apion** | **Apion loti** | |  |
| **Insecta** | **Coleoptera** | **Byturidae** | **Byturus** | **Byturus tomentosus** | |  |
| **Insecta** | **Coleoptera** | **Cantharidae** | **Rhagonycha** | **Rhagonycha fulva** | |  |
| **Insecta** | **Coleoptera** | **Carabidae** | **Amara** | **Amara similata** | |  |
| **Insecta** | **Coleoptera** | **Carabidae** | **Calathus** | **Calathus sp.*** | | **Calathus cinctus, Calathus micropterus, Calathus melanocephalus, Calathus ingratus** |
| **Insecta** | **Coleoptera** | **Carabidae** | **Carabus** | **Carabus nemoralis** | |  |
| **Insecta** | **Coleoptera** | **Carabidae** | **Demetrias** | **Demetrias atricapillus** | |  |
| **Insecta** | **Coleoptera** | **Carabidae** | **Olisthopus** | **Olisthopus rotundatus** | |  |
| **Insecta** | **Coleoptera** | **Carabidae** | **Ophonus** | **Ophonus rufibarbis** | |  |
| **Insecta** | **Coleoptera** | **Carabidae** | **Pterostichus** | **Pterostichus melanarius** | |  |
| **Insecta** | **Coleoptera** | **Cerambycidae** | **Leptura** | **Leptura quadrifasciata** | |  |
| **Insecta** | **Coleoptera** | **Cerambycidae** | **Rhagium** | **Rhagium mordax** | |  |
| **Insecta** | **Coleoptera** | **Chrysomelidae** | **Chaetocnema** | **Chaetocnema picipes** | | **Chaetocnema picipes, Chaetocnema laevicollis (synonym of picipes)** |
| **Insecta** | **Coleoptera** | **Chrysomelidae** | **Oulema** | **Oulema sp.*** | | **Oulema duftschmidi, Oulema melanopus** |
| **Insecta** | **Coleoptera** | **Coccinellidae** | **Scymnus** | **Scymnus schmidti** | | **Scymnus mimulus (syn), Scymnus schmidti** |
| **Insecta** | **Coleoptera** | **Curculionidae** | **Ceutorhynchus** | **Ceutorhynchus fennicus** | |  |
| **Insecta** | **Coleoptera** | **Curculionidae** | **Ceutorhynchus** | **Ceutorhynchus sp.*** | | **Ceutorhynchus typhae (syn. floralis), Ceutorhynchus floralis, Ceutorhynchus cakilis** |
| **Insecta** | **Coleoptera** | **Curculionidae** | **Hypera** | **Hypera meles** | |  |
| **Insecta** | **Coleoptera** | **Melyridae** | **Dasytes** | **Dasytes plumbeus** | |  |
| **Insecta** | **Coleoptera** | **Nitidulidae** | **Meligethes** | **Meligethes aeneus** | |  |
| **Insecta** | **Coleoptera** | **Nitidulidae** | **Meligethes** | **Meligethes carinulatus** | |  |
| **Insecta** | **Coleoptera** | **Nitidulidae** | **Meligethes** | **Meligethes planiusculus** | |  |
| **Insecta** | **Diptera** | **Anthomyiidae** | **Botanophila** | **Botanophila fugax DK*** | | **Botanophila fugax, Botanophila profuga (not in DK), Botanophila hucketti (not in DK)** |
| **Insecta** | **Diptera** | **Anthomyiidae** | **Delia** | **Delia platura** | |  |
| **Insecta** | **Diptera** | **Anthomyiidae** | **Delia** | **Delia radicum** | |  |
| **Insecta** | **Diptera** | **Anthomyzidae** | **Anthomyza** | **Anthomyza collini DK*** | | **Anthomyza anderssoni (not in DK), Anthomyza collini** |
| **Insecta** | **Diptera** | **Anthomyzidae** | **Anthomyza** | **Anthomyza gracilis** | |  |
| **Insecta** | **Diptera** | **Bibionidae** | **Dilophus** | **Dilophus febrilis** | |  |
| **Insecta** | **Diptera** | **Calliphoridae** | **Angioneura** | **Angioneura acerba** | |  |
| **Insecta** | **Diptera** | **Calliphoridae** | **Bellardia** | **Bellardia sp.** | | **Bellardia viarum, Bellardia vulgaris** |
| **Insecta** | **Diptera** | **Calliphoridae** | **Lucilia** | **Lucilia caesar** | |  |
| **Insecta** | **Diptera** | **Calliphoridae** | **Lucilia** | **Lucilia sp.*** | | **Lucilia sericata, Lucilia illustris, Lucilia cuprina** |
| **Insecta** | **Diptera** | **Calliphoridae** | **Melinda** | **Melinda gentilis** | |  |
| **Insecta** | **Diptera** | **Cecidomyiidae** | **Asteromyia** | **Asteromyia sp.^** | | **Unverified. A Nearctic genus** |
| **Insecta** | **Diptera** | **Cecidomyiidae** | **NA** | **Cecidomyiidae sp.1** | | **Cecidomyiidae spp.** |
| **Insecta** | **Diptera** | **Cecidomyiidae** | **NA** | **Cecidomyiidae sp.2** | | **Cecidomyiidae spp.** |
| **Insecta** | **Diptera** | **Cecidomyiidae** | **NA** | **Cecidomyiidae sp.3** | | **Cecidomyiidae spp.** |
| **Insecta** | **Diptera** | **Cecidomyiidae** | **NA** | **Cecidomyiidae sp.4** | | **Cecidomyiidae spp.** |
| **Insecta** | **Diptera** | **Cecidomyiidae** | **NA** | **Cecidomyiidae sp.5** | | **Cecidomyiidae spp.** |
| **Insecta** | **Diptera** | **Cecidomyiidae** | **NA** | **Cecidomyiidae sp.6** | | **Cecidomyiidae spp.** |
| **Insecta** | **Diptera** | **Cecidomyiidae** | **NA** | **Cecidomyiidae sp.7** | | **Cecidomyiidae spp.** |
| **Insecta** | **Diptera** | **Cecidomyiidae** | **NA** | **Cecidomyiidae sp.8** | | **Dasineura leguminicola, Dasineura mali, Aphidoletes aphidimyza** |
| **Insecta** | **Diptera** | **Cecidomyiidae** | **Ozirhincus** | **Ozirhincus longicollis** | | **Ozirhincus hungaricus (non-DK), Ozirhincus tanaceti (syn longicollis, Asphondylia sp.** |
| **Insecta** | **Diptera** | **Cecidomyiidae** | **Peromyia** | **Peromyia sp.** | | **Peromyia horridula and Peromyia muscorum not in BOLD** |
| **Insecta** | **Diptera** | **Cecidomyiidae** | **Rhopalomyia** | **Rhopalomyia sp.** | |  |
| **Insecta** | **Diptera** | **Ceratopogonidae** | **Culicoides** | **Culicoides impunctatus** | |  |
| **Insecta** | **Diptera** | **Ceratopogonidae** | **Dasyhelea** | **Dasyhelea sp.** | | **YES, but DK species: Dasyhelea corinneae, Dasyhelea dufouri, Dasyhelea flavoscutellata, Dasyhelea septuosa, Dasyhelea versicolor NOT in BOLD** |
| **Insecta** | **Diptera** | **Ceratopogonidae** | **Forcipomyia** | **Forcipomyia sp.** | | **99% match** |
| **Insecta** | **Diptera** | **Chamaemyiidae** | **Leucopis** | **Leucopis sp.** | |  |
| **Insecta** | **Diptera** | **Chironomidae** | **Bryophaenocladius** | **Bryophaenocladius sp.** | |  |
| **Insecta** | **Diptera** | **Chironomidae** | **Cladotanytarsus** | **Cladotanytarsus sp.** | | **Cladotanytarsus wexionensis (syn. bicornutus), Cladotanytarsus bicornutus, BUT Cladotanytarsus lepidocalcar NOT in BOLD** |
| **Insecta** | **Diptera** | **Chironomidae** | **Corynoneura** | **Corynoneura sp.** | |  |
| **Insecta** | **Diptera** | **Chironomidae** | **Cricotopus** | **Cricotopus ornatus** | |  |
| **Insecta** | **Diptera** | **Chironomidae** | **Glyptotendipes** | **Glyptotendipes sp.** | | **Glyptotendipes pallens, Glyptotendipes paripes** |
| **Insecta** | **Diptera** | **Chironomidae** | **Guttipelopia** | **Guttipelopia guttipennis** | |  |
| **Insecta** | **Diptera** | **Chironomidae** | **Halocladius** | **Halocladius variabilis** | |  |
| **Insecta** | **Diptera** | **Chironomidae** | **Halocladius** | **Halocladius varians** | |  |
| **Insecta** | **Diptera** | **Chironomidae** | **Metriocnemus** | **Metriocnemus fuscipes** | |  |
| **Insecta** | **Diptera** | **Chironomidae** | **Micropsectra** | **Micropsectra sp.** | | **Micropsectra notescens, Micropsectra apposita** |
| **Insecta** | **Diptera** | **Chironomidae** | **Parakiefferiella** | **Parakiefferiella coronata (perhasp new to DK?)** | | **All 2 DK species in BOLD** |
| **Insecta** | **Diptera** | **Chironomidae** | **Pseudosmittia** | **Pseudosmittia albipennis** | |  |
| **Insecta** | **Diptera** | **Chironomidae** | **Pseudosmittia** | **Pseudosmittia trilobata (perhasp new to DK?)** | | **All 3 DK species in BOLD** |
| **Insecta** | **Diptera** | **Chironomidae** | **Smittia** | **Smittia pratorum** | |  |
| **Insecta** | **Diptera** | **Chironomidae** | **Smittia** | **Smittia sp. (pratorum)** | |  |
| **Insecta** | **Diptera** | **Chloropidae** | **Elachiptera** | **Elachiptera cornuta DK*** | | **Elachiptera decipiens (N. America), Elachiptera cornuta** |
| **Insecta** | **Diptera** | **Chloropidae** | **Meromyza** | **Meromyza sp.** | | **YES, but 3 DK Meromyza species not in BOLD** |
| **Insecta** | **Diptera** | **Chloropidae** | **Oscinella** | **Oscinella sp.** | |  |
| **Insecta** | **Diptera** | **Chloropidae** | **Siphonella** | **Siphonella oscinina** | |  |
| **Insecta** | **Diptera** | **Culicidae** | **Culex** | **Culex pipiens DK*** | | **Culex pipiens, Culex quinquefasciatus (non-EU)** |
| **Insecta** | **Diptera** | **Culicidae** | **Culex** | **Culex sp.** | | **99% match** |
| **Insecta** | **Diptera** | **Culicidae** | **Ochlerotatus** | **Ochlerotatus detritus** | | **Aedes detritus, Ochlerotatus detritus (Aedes is synonym of Ochlerotatus)** |
| **Insecta** | **Diptera** | **Culicidae** | **Ochlerotatus** | **Ochlerotatus sp*** | | **Aedes dorsalis, Aedes caspius (Aedes is synonym of Ochlerotatus)** |
| **Insecta** | **Diptera** | **Dolichopodidae** | **Chrysotus** | **Chrysotus sp.** | | **Chrysotus femoratus, Chrysotus neglectus, Chrysotus gramineus** |
| **Insecta** | **Diptera** | **Dolichopodidae** | **Dolichopus** | **Dolichopus sp.** | | **Dolichopus spp.** |
| **Insecta** | **Diptera** | **Drosophilidae** | **Drosophila** | **Drosophila fenestrarum** | |  |
| **Insecta** | **Diptera** | **Drosophilidae** | **Scaptomyza** | **Scaptomyza sp.** | | **Scaptomyza flava, Scaptomyza pallida** |
| **Insecta** | **Diptera** | **Fanniidae** | **Fannia** | **Fannia similis** | |  |
| **Insecta** | **Diptera** | **Limoniidae** | **Helius** | **Helius longirostris** | |  |
| **Insecta** | **Diptera** | **Lonchopteridae** | **Lonchoptera** | **Lonchoptera bifurcata** | |  |
| **Insecta** | **Diptera** | **Muscidae** | **Coenosia** | **Coenosia tigrina** | |  |
| **Insecta** | **Diptera** | **Muscidae** | **Helina** | **Helina sp.** | | **99% match** |
| **Insecta** | **Diptera** | **Muscidae** | **Hydrotaea** | **Hydrotaea albipuncta** | |  |
| **Insecta** | **Diptera** | **Muscidae** | **Morellia** | **Morellia aenescens** | |  |
| **Insecta** | **Diptera** | **Muscidae** | **Morellia** | **Morellia hortorum** | |  |
| **Insecta** | **Diptera** | **Muscidae** | **Musca** | **Musca autumnalis** | |  |
| **Insecta** | **Diptera** | **Muscidae** | **Neomyia** | **Neomyia cornicina** | | **Dasyphora sp., Neomyia cornicina** |
| **Insecta** | **Diptera** | **Muscidae** | **Phaonia** | **Phaonia tuguriorum** | | **Phaonia tuguriorum, Phaonia sp.** |
| **Insecta** | **Diptera** | **Muscidae** | **Polietes** | **Polietes domitor** | |  |
| **Insecta** | **Diptera** | **NA** | **NA** | **Diptera sp.** | | **Diptera spp.** |
| **Insecta** | **Diptera** | **Pipunculidae** | **Cephalops** | **Cephalops semifumosus DK*** | | **Cephalops digitatus (N.America), Cephalops varipes (syn. semifumosus), Cephalops hardyi (N.America)** |
| **Insecta** | **Diptera** | **Psychodidae** | **Psychoda** | **Psychoda sp.** | | **Psychoda parthenogenetica not in BOLD** |
| **Insecta** | **Diptera** | **Sarcophagidae** | **Macronychia** | **Macronychia polyodon** | |  |
| **Insecta** | **Diptera** | **Sarcophagidae** | **Sarcophaga** | **Sarcophaga sp.** | | **Sarcophaga haemorrhoa, Sarcophaga bulgarica, Sarcophaga depressifrons** |
| **Insecta** | **Diptera** | **Scatopsidae** | **Coboldia** | **Coboldia fuscipes** | |  |
| **Insecta** | **Diptera** | **Scatopsidae** | **NA** | **Scatopsidae sp.** | | **Scatopsidae sp., etc** |
| **Insecta** | **Diptera** | **Sciaridae** | **Bradysia** | **Bradysia nitidicollis** | |  |
| **Insecta** | **Diptera** | **Sciaridae** | **Hyperlasion** | **Hyperlasion wasmanni** | |  |
| **Insecta** | **Diptera** | **Sciaridae** | **Schwenckfeldina** | **Schwenckfeldina carbonaria** | |  |
| **Insecta** | **Diptera** | **Sepsidae** | **Sepsis** | **Sepsis cynipsea DK*** | | **Sepsis cynipsea, Sepsis neocynipsea (not in DK)** |
| **Insecta** | **Diptera** | **Syrphidae** | **Eristalis** | **Eristalis pertinax** | |  |
| **Insecta** | **Diptera** | **Syrphidae** | **Melanostoma** | **Melanostoma mellinum** | |  |
| **Insecta** | **Diptera** | **Syrphidae** | **Meliscaeva** | **Meliscaeva cinctella** | |  |
| **Insecta** | **Diptera** | **Syrphidae** | **Platycheirus** | **Platycheirus clypeatus DK*** | | **Platycheirus clypeatus, Platycheirus quadratus (N.America)** |
| **Insecta** | **Diptera** | **Syrphidae** | **Sphaerophoria** | **Sphaerophoria sp.** | | **Sphaerophoria spp.** |
| **Insecta** | **Diptera** | **Syrphidae** | **Syrphus** | **Syrphus ribesii** | |  |
| **Insecta** | **Diptera** | **Syrphidae** | **Syrphus** | **Syrphus vitripennis DK*** | | **Syrphus vitripennis, Syrphus sexmaculatus, Syrphus opinator, Syrphus rectus** |
| **Insecta** | **Diptera** | **Tabanidae** | **Haematopota** | **Haematopota sp.** | | **Haematopota pluvialis, Haematopota subcylindrica** |
| **Insecta** | **Diptera** | **Tachinidae** | **Blondelia** | **Blondelia migripes** | |  |
| **Insecta** | **Diptera** | **Tachinidae** | **Dinera** | **Dinera ferina** | |  |
| **Insecta** | **Diptera** | **Tachinidae** | **Lydina** | **Lydina aenea** | |  |
| **Insecta** | **Diptera** | **Tachinidae** | **Phasia** | **Phasia hemiptera** | |  |
| **Insecta** | **Diptera** | **Tachinidae** | **Phryxe** | **Phryxe vulgaris DK*** | | **Phryxe vulgaris, Phryxe pecosensis (N.America)** |
| **Insecta** | **Diptera** | **Tachinidae** | **Prosena** | **Prosena siberita** | |  |
| **Insecta** | **Diptera** | **Tachinidae** | **Siphona** | **Siphona geniculata** | |  |
| **Insecta** | **Diptera** | **Tachinidae** | **Voria** | **Voria ruralis** | |  |
| **Insecta** | **Ephemeroptera** | **Baetidae** | **Cloeon** | **Cloeon dipterum** | |  |
| **Insecta** | **Hemiptera** | **Adelgidae** | **Pineus** | **Pineus sp.** | | **Pineus pini, Pineus orientalis, Pineus strobi** |
| **Insecta** | **Hemiptera** | **Anthocoridae** | **Orius** | **Orius laticollis** | |  |
| **Insecta** | **Hemiptera** | **Anthocoridae** | **Orius** | **Orius niger** | |  |
| **Insecta** | **Hemiptera** | **Anthocoridae** | **Orius** | **Orius sp.** | | **Orius minutus, Orius laticollis, Orius vicinus, Orius horvathi** |
| **Insecta** | **Hemiptera** | **Aphididae** | **Aphis** | **Aphis craccae** | |  |
| **Insecta** | **Hemiptera** | **Aphididae** | **Aphis** | **Aphis sp.** | | **Aphis fabae, Aphis solanella, Aphis hederae, Aphis ilicis, Aphis viburni** |
| **Insecta** | **Hemiptera** | **Aphididae** | **Drepanosiphum** | **Drepanosiphum platanoidis DK*** | | **Drepanosiphum platanoidis, Drepanosiphum oregonensis (not in DK)** |
| **Insecta** | **Hemiptera** | **Aphididae** | **Euceraphis** | **Euceraphis betulae** | |  |
| **Insecta** | **Hemiptera** | **Aphididae** | **Hyadaphis** | **Hyadaphis foeniculi DK*** | | **YES, but some wrong ID specimens are foeniculi** |
| **Insecta** | **Hemiptera** | **Aphididae** | **Hyalopterus** | **Hyalopterus pruni** | |  |
| **Insecta** | **Hemiptera** | **Aphididae** | **Phyllaphis** | **Phyllaphis fagi** | |  |
| **Insecta** | **Hemiptera** | **Aphididae** | **Schizaphis** | **Schizaphis sp.** | | **99% match** |
| **Insecta** | **Hemiptera** | **Aphididae** | **Semiaphis** | **Semiaphis dauci** | |  |
| **Insecta** | **Hemiptera** | **Aphrophoridae** | **Philaenus** | **Philaenus spumarius** | |  |
| **Insecta** | **Hemiptera** | **Lachnidae** | **Cinara** | **Cinara pruinosa** | |  |
| **Insecta** | **Hemiptera** | **Miridae** | **Adelphocoris** | **Adelphocoris lineolatus** | | **Adelphocoris lineolatus, Adelphocoris triannulatus (not in EU), Adelphocoris suturalis (not in EU)** |
| **Insecta** | **Hemiptera** | **Miridae** | **Lygus** | **Lygus rugulipennis** | | **Lygus rugulipennis, Lygus lineolaris (N.America)** |
| **Insecta** | **Hemiptera** | **Miridae** | **Neolygus** | **Neolygus sp.** | | **Neolygus contaminatus, Neolygus viridis** |
| **Insecta** | **Hemiptera** | **Miridae** | **Orthops** | **Orthops basalis** | |  |
| **Insecta** | **Hemiptera** | **Miridae** | **Orthops** | **Orthops campestris** | |  |
| **Insecta** | **Hemiptera** | **Miridae** | **Phytocoris** | **Phytocoris varipes** | |  |
| **Insecta** | **Hemiptera** | **Pentatomidae** | **Palomena** | **Palomena prasina** | |  |
| **Insecta** | **Hymenoptera** | **Braconidae** | **Aphidius** | **Aphidius avenae** | |  |
| **Insecta** | **Hymenoptera** | **Braconidae** | **Lysiphlebus** | **Lysiphlebus hirticornis** | | **Lysiphlebus hirticornis, Lysiphlebus fabarum (not in DK), Lysiphlebus cardui (not in DK)** |
| **Insecta** | **Hymenoptera** | **Braconidae** | **Praon** | **Praon sp. (perhaps new to DK?)** | | **Praon longicorne, Praon volucre, all DK species in BOLD** |
| **Insecta** | **Hymenoptera** | **Ichneumonidae** | **Promethes** | **Promethes sulcator** | |  |
| **Insecta** | **Hymenoptera** | **Tenthredinidae** | **Athalia** | **Athalia rosae** | |  |
| **Insecta** | **Hymenoptera** | **Tenthredinidae** | **Dolerus** | **Dolerus germanicus** | | **Dolerus germanicus, Dolerus abstrusus (Not in EU)** |
| **Insecta** | **Hymenoptera** | **Tenthredinidae** | **NA** | **Tenthredinidae sp.** | | **Nematinus acuminatus, Dineura pullior, Dineura viridodorsata** |
| **Insecta** | **Hymenoptera** | **Tenthredinidae** | **Tenthredo** | **Tenthredo sp.** | | **Tenthredo spp. (T. notha, T. arcuata, etc.)** |
| **Insecta** | **Lepidoptera** | **Crambidae** | **Agriphila** | **Agriphila sp.** | | **Agriphila selasella, Agriphila tristella** |
| **Insecta** | **Lepidoptera** | **Crambidae** | **Pleuroptya** | **Pleuroptya ruralis DK*** | | **Several matches, only ruralis in DK** |
| **Insecta** | **Lepidoptera** | **Elachistidae** | **Elachista** | **Elachista nobilella** | |  |
| **Insecta** | **Lepidoptera** | **Erebidae** | **Eilema** | **Eilema griseola** | |  |
| **Insecta** | **Lepidoptera** | **Gelechiidae** | **Aproaerema** | **Aproaerema anthyllidella** | |  |
| **Insecta** | **Lepidoptera** | **Gelechiidae** | **Isophrictis** | **Isophrictis striatella** | | **Isophrictis striatella, Lepidoptera spp.?(non-DK)** |
| **Insecta** | **Lepidoptera** | **Geometridae** | **Acasis** | **Acasis viretata** | |  |
| **Insecta** | **Lepidoptera** | **Geometridae** | **Chloroclystis** | **Chloroclystis v-ata** | | **Chloroclystis v-ata, Melanapamea mixta (non-EU)** |
| **Insecta** | **Lepidoptera** | **Geometridae** | **Eupithecia** | **Eupithecia absinthiata DK*** | | **exotic Lepidoptera, Eupithecia absinthiata, Eupithecia expallidata (non-DK)** |
| **Insecta** | **Lepidoptera** | **Geometridae** | **Eupithecia** | **Eupithecia tripunctaria** | |  |
| **Insecta** | **Lepidoptera** | **Geometridae** | **Eupithecia** | **Eupithecia virgaureata DK*** | | **Eupithecia virgaureata, Eupithecia sharronata (non-DK)** |
| **Insecta** | **Lepidoptera** | **Geometridae** | **Scotopteryx** | **Scotopteryx chenopodiata** | |  |
| **Insecta** | **Lepidoptera** | **Hesperiidae** | **Thymelicus** | **Thymelicus lineola** | | **Thymelicus lineola, non-DK Lepidoptera** |
| **Insecta** | **Lepidoptera** | **Momphidae** | **Mompha** | **Mompha epilobiella** | |  |
| **Insecta** | **Lepidoptera** | **NA** | **NA** | **Lepidoptera sp.1** | | **Lepidoptera spp.** |
| **Insecta** | **Lepidoptera** | **NA** | **NA** | **Lepidoptera sp.2** | | **Lepidoptera spp.** |
| **Insecta** | **Lepidoptera** | **NA** | **NA** | **Lepidoptera sp.3** | | **Lepidoptera spp.** |
| **Insecta** | **Lepidoptera** | **NA** | **NA** | **Lepidoptera sp.4** | |  |
| **Insecta** | **Lepidoptera** | **NA** | **NA** | **Lepidoptera sp.5** | |  |
| **Insecta** | **Lepidoptera** | **NA** | **NA** | **Lepidoptera sp.6** | |  |
| **Insecta** | **Lepidoptera** | **NA** | **NA** | **Lepidoptera sp.7** | | **Lepidoptera spp.** |
| **Insecta** | **Lepidoptera** | **Noctuidae** | **Apamea** | **Apamea anceps** | | **Apamea anceps, Apamea pyxina (non-EU), Afotella cylindrica (non-EU)** |
| **Insecta** | **Lepidoptera** | **Noctuidae** | **Apamea** | **Apamea sordens** | |  |
| **Insecta** | **Lepidoptera** | **Noctuidae** | **Autographa** | **Autographa gamma** | |  |
| **Insecta** | **Lepidoptera** | **Noctuidae** | **Mythimna** | **Mythimna sp.** | | **Mythimna pallens, Mythimna favicolor** |
| **Insecta** | **Lepidoptera** | **Noctuidae** | **Phlogophora** | **Phlogophora meticulosa** | |  |
| **Insecta** | **Lepidoptera** | **Nymphalidae** | **Aphantopus** | **Aphantopus hyperantus** | | **Aphantopus hyperantus, Aphantopus bieti (non-EU)** |
| **Insecta** | **Lepidoptera** | **Oecophoridae** | **Hofmannophila** | **Hofmannophila pseudospretella** | | **Lepidoptera spp. (non-EU), and single hit to Plemyria rubiginata** |
| **Insecta** | **Lepidoptera** | **Psychidae** | **Dahlica** | **Dahlica sp.** | | **Dahlica lichenella, Dahlica lazuri, Dahlica charlottae (not in DK), Dahlica wehrlii (not in DK), Dahlica goppensteinensis (not in DK), Dahlica generosensis (not in DK)** |
| **Insecta** | **Lepidoptera** | **Pterophoridae** | **Gillmeria** | **Gillmeria ochrodactyla** | | **Gillmeria ochrodactyla, Platyptilia tetradactyla (synonym)** |
| **Insecta** | **Lepidoptera** | **Tortricidae** | **Dichrorampha** | **Dichrorampha obscuratana** | | **Dahlica lichenella** |
| **Insecta** | **Lepidoptera** | **Tortricidae** | **Eucosma** | **Eucosma cana** | |  |
| **Insecta** | **Lepidoptera** | **Tortricidae** | **Eucosma** | **Eucosma hohenwartiana/fulvana/parvulana complex** | |  |
| **Insecta** | **Neuroptera** | **Hemerobiidae** | **Micromus** | **Micromus variegatus** | |  |
| **Insecta** | **Orthoptera** | **Tettigoniidae** | **Leptophyes** | **Leptophyes punctatissima** | |  |
| **Insecta** | **Psocoptera** | **Caeciliusidae** | **Valenzuela** | **Valenzuela flavidus** | |  |
| **Insecta** | **Psocoptera** | **Ectopsocidae** | **Ectopsocus** | **Ectopsocus briggsi** | | **match to other non-EU species** |
| **Insecta** | **Psocoptera** | **Lachesillidae** | **Lachesilla** | **Lachesilla pedicularia** | |  |
| **Insecta** | **Psocoptera** | **Peripsocidae** | **Peripsocus** | **Peripsocus subfasciatus** | |  |
| **Insecta** | **Thysanoptera** | **Aeolothripidae** | **Aeolothrips** | **Aeolothrips fasciatus** | |  |
| **Insecta** | **Thysanoptera** | **Thripidae** | **Thrips** | **Thrips major** | |  |
| **Insecta** | **Thysanoptera** | **Thripidae** | **Thrips** | **Thrips tabaci** | |  |
| **Malacostraca** | **Isopoda** | **Asellidae** | **Asellus** | **Asellus aquaticus** | |  |
| **Malacostraca** | **Isopoda** | **Philosciidae** | **Philoscia** | **Philoscia muscorum** | |  |
| **Mollusca** | **Gastropoda** | **Agriolimacidae** | **Deroceras** | **Deroceras agreste** | |  |
| **Mollusca** | **Gastropoda** | **Agriolimacidae** | **Deroceras** | **Deroceras invadens** | | **synonym: Deroceras panormitanum** |
| **Mollusca** | **Gastropoda** | **Arionidae** | **Arion** | **Arion cf. vulgaris** | | **99% match, Arion vulgaris (synonym of A. lusitanicus)** |
| **Mollusca** | **Gastropoda** | **Bradybaenidae** | **Fruticicola** | **Fruticicola fruticum** | | **99% match** |
| **Nematoda** | **Chromadorea** | **Steinernematidae** | **Steinernema** | **Steinernema feltiae** | |  |
| **Rotifera** | **Bdelloidea** | **Adinetidae** | **Adineta** | **Adineta vaga** | |  |
| **Rotifera** | **Bdelloidea** | **Habrotrochidae** | **Habrotrocha** | **Habrotrocha elusa** | |  |
| **Heterokontophyta** | **Oomycetes** | **Albuginaceae** | **Albugo** | **Albugo candida** | |  |
| **Heterokontophyta** | **Oomycetes** | **Peronosporaceae** | **Peronospora** | **Peronospora ervi** | |  |
| **Heterokontophyta** | **Oomycetes** | **Peronosporaceae** | **Peronospora** | **Peronospora obovata** | |  |
| **Heterokontophyta** | **Oomycetes** | **Peronosporaceae** | **Peronospora** | **Peronospora radii** | |  |
| **Heterokontophyta** | **Oomycetes** | **Peronosporaceae** | **Peronospora** | **Peronospora trifolii-minoris** | |  |
| **Heterokontophyta** | **Oomycetes** | **Peronosporaceae** | **Peronospora** | **Peronospora trifoliorum** | |  |
|  |  |  |  |  | |  |
| **16S** | | | | | | |
| **Class** | **Order** | **Family** | **Genus** | | **Species** | **Identification notes** |
| **Arachnida** | **Opiliones** | **Leiobunidae** | **Leiobunum** | | **Leiobunum rotundum** | **Leiobunum rotundum, 100** |
| **Collembola** | **Entomobryomorpha** | **Entomobryidae** | **Willowsia** | | **Willowsia nigromaculata** | **Willowsia nigromaculata, 100** |
| **Collembola** | **Entomobryomorpha** | **Isotomidae** | **Isotoma** | | **Isotoma viridis** | **Isotoma viridis, 100** |
| **Insecta** | **Coleoptera** | **Brentidae** | **Apion** | | **Apion fulvipes** | **Protapion fulvipes, 100.000, Protapion fulvipes, 99.363, Protapion nigritarse, 97.452** |
| **Insecta** | **Coleoptera** | **Carabidae** | **Carabus** | | **Carabus nemoralis DK*** | **Carabus nemoralis, 100.000, Carabus pseudomonticola, 100.000, Carabus nemoralis nemoralis, 99.383, Carabus nemoralis, 98.137, Carabus nemoralis prasinotinctus, 98.137, Carabus steuartii, 96.273, Carabus famini numidus, 95.679, Carabus problematicus, 95.062, Carabus famini, 95.062, Carabus mikhaili loustei, 95.062, Carabus riffensis, 95.062, Carabus tibetanophilus yak, 95.031** |
| **Insecta** | **Coleoptera** | **Coccinellidae** | **Coccinella** | | **Coccinella septempunctata** | **Coccinella septempunctata, 100** |
| **Insecta** | **Coleoptera** | **Coccinellidae** | **Harmonia** | | **Harmonia axyridis** | **Harmonia axyridis, 100** |
| **Insecta** | **Coleoptera** | **Coccinellidae** | **Tytthaspis** | | **Tytthaspis sedecimpunctata** | **Tytthaspis sedecimpunctata, 100** |
| **Insecta** | **Coleoptera** | **Curculionidae** | **Tychius** | | **Tychius picirostris** | **Tychius picirostris, 100** |
| **Insecta** | **Coleoptera** | **Dermestidae** | **Trogoderma** | | **Trogoderma sp.** | **Trogoderma teukton, 100. T. Angustum, T. Versicolor NOT in genbank** |
| **Insecta** | **Coleoptera** | **Nitidulidae** | **Meligethes** | | **Meligethes planiusculus** | **Meligethes planiusculus, 100.000, Meligethes isoplexidis, 96.226, Meligethes canariensis, 96.226, Meligethes conformis, 95.597** |
| **Insecta** | **Coleoptera** | **Nitidulidae** | **Meligethes** | | **Meligethes sp. (aeneus)** | **Meligethes sp. TJH-2004, 100.000, Brassicogethes aeneus, 99.359** |
| **Insecta** | **Coleoptera** | **Phalacridae** | **Stilbus** | | **Stilbus testaceus** | **Stilbus testaceus, 100.000, Pedetontinus luanchuanensis, 97.059** |
| **Insecta** | **Dermaptera** | **Forficulidae** | **Forficula** | | **Forficula auricularia** | **Forficula auricularia, 100.000, Forficula auricularia, 99.367, Forficula auricularia A, 99.367, Forficula auricularia, 98.734, Forficula auricularia, 98.101** |
| **Insecta** | **Diptera** | **Calliphoridae** | **Lucilia** | | **Lucilia caesar** | **Lucilia caesar, 100.000, Lucilia cuprina, 99.363, Lucilia illustris, 99.363, Lucilia caesar, 99.363, Lucilia cf. illustris/caesar LUCIL104-12, 99.363, Lucilia cuprina, 98.726, Lucilia caesar, 98.726, Lucilia ampullacea, 98.726, Lucilia porphyrina, 98.726, Lucilia caesar, 98.089, Lucilia porphyrina, 98.089, Lucilia cuprina, 97.452, Lucilia porphyrina, 97.452** |
| **Insecta** | **Diptera** | **Calliphoridae** | **Pollenia** | | **Pollenia rudis** | **Pollenia rudis, 100** |
| **Insecta** | **Diptera** | **Culicidae** | **Culex** | | **Culex sp.** | **Wuchereria bancrofti, 100, Culex quinquefasciatus, 100, Culex tritaeniorhynchus, 100, Culex pipiens, 100** |
| **Insecta** | **Diptera** | **Dolichopodidae** | **Hercostomus** | | **Hercostomus sp.** | **Hercostomus fulvicaudis, 100, Hercostomus praeceps, 100** |
| **Insecta** | **Diptera** | **Dolichopodidae** | **Medetera** | | **Medetera truncorum** | **Medetera truncorum, 100.000, Medetera truncorum, 99.371** |
| **Insecta** | **Diptera** | **Muscidae** | **Musca** | | **Musca autumnalis** | **Musca autumnalis, 100** |
| **Insecta** | **Diptera** | **Opomyzidae** | **Opomyza** | | **Opomyza florum** | **Opomyza florum, 100** |
| **Insecta** | **Diptera** | **Sciaridae** | **Schwenckfeldina** | | **Schwenckfeldina carbonaria** | **Schwenckfeldina carbonaria, 100** |
| **Insecta** | **Ephemeroptera** | **Baetidae** | **Cloeon** | | **Cloeon dipterum** | **Cloeon dipterum, 100.000, Cloeon dipterum, 98.667** |
| **Insecta** | **Hemiptera** | **Aphididae** | **NA** | | **Aphididae sp.** | **Sitobion avenae, 100.000, Sitobion avenae, 98.374, Amphorophora rubi, 96.748, Myzus philadelphi, 96.552** |
| **Insecta** | **Hemiptera** | **Miridae** | **Adelphocoris** | | **Adelphocoris lineolatus** | **Adelphocoris lineolatus, 100.000, Adelphocoris lineolatus, 98.726, Adelphocoris fasciaticollis, 98.726, Adelphocoris nigritylus, 98.726, Adelphocoris lineolatus, 98.089, Adelphocoris suturalis, 98.089** |
| **Insecta** | **Hemiptera** | **Miridae** | **NA** | | **Miridae sp.1** | **Lygocoris sp. SJ-2010, 100** |
| **Insecta** | **Hemiptera** | **Miridae** | **NA** | | **Miridae sp.3** | **Urocaridella antonbruunii, 100, Urocaridella pulchella, 100, Urocaridella cyrtorhyncha, 100** |
| **Insecta** | **Hemiptera** | **Miridae** | **Plagiognathus** | | **Miridae sp.2** | **Plagiognathus chrysanthemi, 100, Placochilus seladonicus, 100** |
| **Insecta** | **Hemiptera** | **Veliidae** | **Microvelia** | | **Microvelia reticulata** | **Microvelia reticulata, 100.000, Trechus melanocephalus, 96.875** |
| **Insecta** | **Hymenoptera** | **Apidae** | **Bombus** | | **Bombus lapidarius** | **Bombus lapidarius, 100** |
| **Insecta** | **Hymenoptera** | **Apidae** | **Bombus** | | **Bombus soroeensis** | **Bombus soroeensis, 100** |
| **Insecta** | **Orthoptera** | **Acrididae** | **NA** | | **Acrididae sp.** | **Gomphocerippus rufus, 100.000, Chorthippus jacobsi, 100.000, Euthystira brachyptera, 100.000, Dnopherula albonemus, 100.000, Gomphocerippus rufus, 99.355, Chorthippus jacobsi, 99.355, Chorthippus albonemus, 97.419, Stauroderus scalaris, 96.129, Chorthippus chinensis, 96.129, Chorthippus hammarstroemi, 95.541, Gomphocerus sibiricus tibetanus, 95.484, Gomphocerus licenti, 95.484, Gomphocerus sibiricus, 95.484** |
| **Insecta** | **Thysanoptera** | **Thripidae** | **Anaphothrips** | | **Anaphothrips obscurus** | **Anaphothrips obscurus, 100** |
| **Insecta** | **Thysanoptera** | **Thripidae** | **NA** | | **Thripidae sp.** | **Erianthus serratus, 100, Meimuna opalifera, 100, Erianthus versicolor, 100, Meimuna choui, 100, Dactylocerca rubra, 100, Phylinae aff. Stoebea sp. 726, 100** |
| **Malacostraca** | **Decapoda** | **Astacidae** | **Pacifastacus** | | **Pacifastacus leniusculus** | **Pacifastacus leniusculus, 100.000, Pacifastacus leniusculus, 99.351, Pacifastacus leniusculus, 99.346, Pacifastacus leniusculus, 98.701, Pacifastacus leniusculus, 98.693, Pacifastacus leniusculus, 98.065, Pacifastacus leniusculus, 98.052, Pacifastacus leniusculus, 98.039** |
| **Malacostraca** | **Decapoda** | **Palaemonidae** | **Palaemon** | | **Palaemon adspersus** | **Palaemon adspersus, 100.000, Palaemon adspersus, 99.375** |
| **Malacostraca** | **Isopoda** | **Asellidae** | **Asellus** | | **Asellus aquaticus** | **Asellus aquaticus, 100.000, Asellus aquaticus, 99.265** |
| **Malacostraca** | **Isopoda** | **Asellidae** | **NA** | | **Isopoda sp.** | **Philoscia musorum NOT in Genbank! Proasellus coxalis, 100.000, Proasellus coxalis, 99.265, Proasellus coxalis, 98.529, Proasellus coxalis, 95.588** |
| **Branchiopoda** | **Diplostraca** | **Polyphemidae** | **Polyphemus** | | **Polyphemus pediculus** | **Polyphemus pediculus, 100.000, Podon leuckartii, 100.000, Polyphemus pediculus, 99.346, Polyphemus pediculus, 98.693** |
| **Gastropoda** | **Stylommatophora** | **Helicidae** | **Arianta** | | **Arianta arbustorum** | **Arianta arbustorum, 100.000, Theba geminata, 95.122, Theba impugnata, 95.122, Theba sp. 1b CG-2011, 95.122, Theba sp. 4 CG-2011, 95.122** |
| **Gastropoda** | **Stylommatophora** | **Helicidae** | **Cornu** | | **Cornu aspersum** | **Helix aspersa, 100** |
| **Clitellata** | **Haplotaxida** | **Enchytraeidae** | **NA** | | **Enchytraeidae sp.** | **Diplocardia komareki, 100.000, Cernosvitoviella minor, 100.000, Parachilota sp. 561, 100.000, Euenchytraeus clarae, 100.000, Henlea ventriculosa, 97.581, N/A, 97.561, Marionina cf. levitheca PDW-2010, 95.000** |
| **Clitellata** | **Haplotaxida** | **Lumbricidae** | **Aporrectodea** | | **Aporrectodea tuberculata** | **Aporrectodea tuberculata, 100.000, Aporrectodea tuberculata, 99.237, Aporrectodea tuberculata, 98.473, Aporrectodea tuberculata, 97.710** |
| **Clitellata** | **Haplotaxida** | **Lumbricidae** | **Eiseniella** | | **Eiseniella tetraedra** | **Eiseniella tetraedra, 100.000, Eiseniella tetraedra, 99.219, Eiseniella tetraedra, 96.094, Eiseniella tetraedra, 95.935, Eiseniella tetraedra, 95.312** |
| **Clitellata** | **Haplotaxida** | **Lumbricidae** | **Lumbricus** | | **Lumbricus rubellus** | **Lumbricus rubellus, 100.000, Lumbricus rubellus, 99.231, Lumbricus rubellus, 98.462, Lumbricus rubellus, 96.923, Lumbricus rubellus, 95.385** |
| **Clitellata** | **Haplotaxida** | **Tubificidae** | **Chaetogaster** | | **Chaetogaster diaphanus** | **Chaetogaster diaphanus, 100** |
| **Clitellata** | **Haplotaxida** | **Tubificidae** | **Stylaria** | | **Stylaria lacustris** | **Stylaria lacustris, 100.000, Stylaria lacustris, 96.875, Ripistes parasita, 96.875, Stylaria fossularis, 95.312** |
| **Hydrozoa** | **Anthoathecata** | **Hydridae** | **Hydra** | | **Hydra circumcincta** | **Hydra circumcincta, 100.000, Hydra circumcincta, 99.535, Hydra circumcincta, 98.605, Hydra circumcincta, 97.209, Hydra utahensis, 95.370** |
| **Actinopteri** | **Esociformes** | **Esocidae** | **Esox** | | **Esox lucius** | **Esox lucius, 100.000, Esox flaviae, 100.000, Esox lucius, 99.533, Esox lucius, 99.065, Esox reichertii, 98.598** |
| **Actinopteri** | **Perciformes** | **Scorpaenidae** | **Pterois** | | **Pterois miles** | **Pterois miles, 100.000, Pterois miles, 99.539, Pterois russelii, 99.539, Pterois miles, 99.078, Pterois volitans, 99.078, Pterois volitans, 98.618, Dendrochirus zebra, 95.392, Pterois radiata, 95.392** |
| **Mammalia** | **Carnivora** | **Canidae** | **Canis** | | **Canis lupus familiaris** | **Canis lupus, 100** |
| **Mammalia** | **Cetartiodactyla** | **Bovidae** | **Bos** | | **Bos taurus** | **Bos primigenius, 100.000, Bos taurus, 100.000, Bos indicus, 100.000, Phascolosoma esculenta, 100.000, Bos taurus, 99.471, Bos indicus, 99.471, Phascolosoma esculenta, 99.471** |
| **Mammalia** | **Cetartiodactyla** | **Bovidae** | **Ovis** | | **Ovis aries** | **Ovis aries, 100, Ovis orientalis, 100** |
| **Mammalia** | **Cetartiodactyla** | **Cervidae** | **Dama** | | **Dama dama** | **Dama dama, 100.000, Cervus elaphus, 95.238, Rusa unicolor, 95.238** |
| **Mammalia** | **Cetartiodactyla** | **Suidae** | **Sus** | | **Sus scrofa** | **Sus scrofa, 100** |
| **Mammalia** | **Perissodactyla** | **Equidae** | **Equus** | | **Equus caballus** | **Equus caballus, 100, Equus przewalskii, 100, Equus lambei, 100** |
| **Mammalia** | **Primates** | **Hominidae** | **Homo** | | **Homo sapiens** | **Homo sapiens, 100.000, Homo sapiens, 99.474** |

**Table S4**. Sequence list for all identified species. DK*) Final taxa ID is determined based on presence in Denmark. Data is given for COI and 16S separately.

| **COI** | |
| --- | --- |
| **Anyphaena accentuate** | **AGCTTGAGCTGCTATAGTAGGAACTGGAATAAGAGTATTAATTCGTATAGAGTTAGGACAATCAGGAAGATTTTTAGGTGATGATCATATATATAATGTGATTGTAACTGCTCATGCTTTTGTAATAATTTTTTTTATAGTAATACCTATTTTAATT** |
| **Araneus quadratus** | **AGCTTGAGCTGCTATAGTTGGAACAGCTATAAGAGTATTAATTCGAATTGAATTAGGTCAACCTGGAAGATTTATGGGTGATGATCAACTTTATAATGTAATTGTAACTGCACATGCGTTTGTTATAATTTTTTTTATGGTAATGCCTATTTTAATT** |
| **Clubiona comta** | **GTCTTGATCTGCTATAGTAGGGACAGCTATAAGAGTATTAATCCGGATAGAGTTAGGACAGTCGGGTGCATTATTAGGTGATGATCATTTATATAATGTAGTAGTAACTGCTCATGCTTTTGTTATAATTTTTTTTATAGTTATACCTATTTTGATT** |
| **Clubiona phragmitis** | **ATCTTGATCTGCTATAGTTGGAACAGCTATAAGAGTATTAATTCGTATGGAATTGGGGCAGTCTGGAATATTTTTAGGGGATGATCATTTATATAATGTAGTAGTTACAGCTCATGCTTTTGTTATAATTTTTTTTATAGTTATACCTATTTTAATT** |
| **Diplocephalus cristatus** | **GGCATGAGCTGCTATAGTAGGGACAGCAATGAGAGTATTAATTCGAATTGAGTTGGGTCAAACTGGAAGATTATTAGGAGATGACCAGTTGTATAATGTTATTGTTACTGCTCATGCGTTTGTGATAATTTTTTTTATAGTTATACCTATTTTAATT** |
| **Neriene clathrata** | **TGCTTGAGCTGCTATGGTAGGGACAGCTATAAGAGTTTTAATTCGAATTGAATTAGGACAAGTTGGAAGAATATTAGGAGATGATCAATTGTATAATGTAATTGTTACAGCTCATGCTTTTGTAATAATTTTTTTTATAGTTATACCAATTTTAATT** |
| **Cheiracanthium sp.*** | **AGCTTGATCAGCTATAGCTGGAACTGCTATAAGTATTATAATTCGTATGGAATTAGGACAGGTTGGATCTTTTTTAGGAGATGATCAGTTATATAATGTTGTAGTAACTGCTCATGCTTTTGTTATAATTTTTTTTATAGTAATACCAATTTTGATT** |
| **Ceratozetidae sp.** | **GGCTTGAGCTGGACTTTTGGGTTCTGCTCTTAGTGGATTAATTCGATTAGAATTAAGACAACCAGGTTCTTTATTAGAAAATGATCAAATTTACAATACAATCGTTACCGCTCATGCTTTTGTCATAATTTTTTTTATGGTTATGCCAGTAATAATT** |
| **Punctoribates punctum** | **GGCCTGAGCTGGACTGTTAGGATCTGCTTTAAGAGGACTAATTCGTTTAGAGCTAGGACAGCCCGGTTCTCTAATAGAGAACGACCAAATTTATAATACTGTAGTAACAGCACATGCTTTTGTGATAATTTTCTTTATAGTGATGCCCGTGATAATT** |
| **Entomobrya sp.** | **AGTTTGAGCCGCCATAGTTGGGACTGCTTTTAGTGTTTTAATTCGCCTTGAATTAGGACAACCAGGAAGATTTATTGGAGATGACCAAATTTATAATGTTATAGTTACTGCACACGCTTTTATTATAATTTTCTTTATAGTTATACCTATTATAATT** |
| **Isotoma sp.** | **AGTTTGATCAGCAATAGTAGGTACTGCTTTTAGAGTTTTAATCCGGTTAGAATTGGGTCAACCAGGAAGATTTATTGGGGATGACCAAATTTATAATGTAATAGTTACCGCCCATGCTTTTATTATAATTTTTTTTATAGTTATACCTATCATAATC** |
| **Apion apricans** | **ACTATGATCAGGAATAATTGGCACTTCATTAAGAATACTAATTCGTATTGAATTAGGTAACCCTGGGTCATTAATTGGTAATGATCAAATTTATAATGTTATTGTCACAGCTCATGCATTTATTATAATTTTTTTTATAGTTATACCTGTTATAATT** |
| **Apion fulvipes** | **TCTTTGATCAGGAATAATTGGTACGTCTTTAAGTATACTAATTCGTATTGAATTAGGAAACCCTGGATCATTAATTGGTAATGACCAAATTTATAATGTTATTGTTACAGCTCATGCTTTTATTATAATTTTCTTTATAGTAATACCAGTAATAATT** |
| **Apion loti** | **ACTATGGTCAGGGATAGCCGGAACTTCATTAAGAATACTAATTCGTATTGAATTAGGGACCCCAGGGTCTTTAATTGGTAATGACCAAATTTATAATGTCATTGTTACAGCCCATGCTTTTATTATAATTTTTTTTATAGTTATACCTATTATAATT** |
| **Byturus tomentosus** | **TGCATGAGCAGGAATAGTGGGAACTTCATTAAGATTACTAATTCGATCTGAATTAGGAAACCCCGGATCATTAATTGGAGATGATCAAATCTACAATGTAATTGTAACAGCCCATGCTTTCATTATAATTTTTTTCATAGTAATACCTATTGTAATT** |
| **Rhagonycha fulva** | **AGCCTGATCGGGATCTTTAGGATTAGCCTTAAGATTATTAATTCGAGCTGAATTAGGAACTCCAGGAACTTTAATTGGTAATGATCAAATTTATAATGTTATTGTTACTGCTCATGCTTTTATCATAATTTTCTTCATAGTTATACCTATTATAATT** |
| **Amara similata** | **AGCATGATCAGGAATAGTAGGTACTTCATTAAGTATATTGATTCGAGCTGAATTAGGAAATCCTGGAGCATTAATTGGTGATGATCAAATTTATAATGTTATTGTCACTGCTCATGCATTTGTTATAATTTTTTTTATAGTAATACCTATTATAATT** |
| **Calathus sp.*** | **TGCATGAGCAGGAATAGTAGGAACATCCTTAAGTATACTTATTCGTGCTGAATTAGGAAATCCAGGAGCATTGATTGGTGATGACCAAATTTATAATGTTATTGTAACTGCCCATGCATTTGTTATAATTTTTTTTATAGTAATACCTATTATAATT** |
| **Carabus nemoralis** | **TGCTTGATCAGGTATAGTGGGAACTTCACTAAGTATACTAATTCGAGCAGAATTAGGAAACCCGGGATCCTTAATTGGAGATGATCAAATTTATAATGTTATTGTAACAGCTCATGCTTTTGTAATAATTTTCTTTATAGTAATACCTATTATAATT** |
| **Demetrias atricapillus** | **TGCTTGAGCCGGTATAGTAGGAACATCCTTAAGAATACTAATTCGAGCAGAATTAGGAAACCCGGGAGCTCTAATTGGTGACGATCAAATTTATAATGTAATTGTAACTGCTCATGCATTTATCATAATTTTTTTTATAGTAATACCAATTATAATT** |
| **Olisthopus rotundatus** | **GGCTTGATCAGGGATAGTAGGAACTTCATTAAGAATATTAATTCGAGCTGAGCTAGGAAATCCTGGTTCATTAATTGGAGATGATCAAATTTATAATGTTATTGTAACTGCTCATGCTTTTATTATAATTTTTTTTATAGTAATGCCAATTATAATT** |
| **Ophonus rufibarbis** | **AGCTTGAGCAGGAATAGTAGGAACTTCATTAAGCATATTAATTCGAGCTGAATTGGGGACTCCTGGAGCATTGATTGGTGATGATCAAATTTATAATGTTATTGTTACTGCACATGCTTTTATTATAATTTTTTTTATAGTTATACCTATTATAATT** |
| **Pterostichus melanarius** | **TGCTTGAGCAGGAATAGTAGGAACTTCATTAAGAATATTAATTCGAGCTGAATTAGGAAATCCTGGATCATTAATTGGTGATGATCAAATTTATAATGTTATTGTAACTGCTCATGCATTTGTTATAATTTTTTTTATAGTAATACCTATTATAATT** |
| **Leptura quadrifasciata** | **AGCCTGGGCAGGCATAGTAGGAACATCACTAAGACTTTTAATTCGATCAGAACTTGGCAATCCTGGATCATTAATCGGTGATGATCAAATTTATAATGTAATTGTTACAGCTCATGCATTTGTAATAATTTTTTTTATAGTTATACCTATTATAATT** |
| **Rhagium mordax** | **AGCTTGATCAGGAATAGTCGGAACATCTTTAAGTTTATTAATTCGATCAGAATTAGGAAATCCAGGATCATTAATTGGTGATGATCAAATTTATAATGTAATTGTTACCGCTCATGCTTTCATTATAATTTTCTTTATAGTTATACCTATTATAATT** |
| **Chaetocnema picipes** | **AATTTGGTCAGGTATAGTTGGAACTTCCCTAAGAGTTCTAATTCGAACAGAACTAGGTAACCCTGGTAGTTTAATTGGAAATGACCAAATTTATAATGTAATTGTAACCGCTCATGCTTTTATTATAATTTTTTTTATAGTGATACCTATTATAATT** |
| **Oulema sp.*** | **GGCCTGATCAGGCATAGTCGGAACATCTTTAAGAATAATAATCCGAACTGAATTAGGAAACCCGGGATCATTAATTGGTAATGATCAAATTTACAATGTAATTGTTACTGCACATGCCTTTATTATAATTTTTTTTATAGTAATGCCAATTATAATT** |
| **Scymnus schmidti** | **ATTATGAGCAGGAATAGTAGGTACATCTTTAAGAATTTTAATTCGCTTAGAACTTGGAACTACAAGAGCATTAATTGGAAACGACCAAATTTATAATGTAATTGTTACAGCTCATGCTTTTATCATAATTTTTTTTATAGTAATACCAATTATAATT** |
| **Ceutorhynchus fennicus** | **ATCCTGAGCTGGAATAGCAGGAACTTCATTAAGAATACTTATCCGAACTGAATTAGGAAACCCTGGTATACTCATTGGAGACGACCAAATTTATAACTCAATTGTAACTGCTCATGCTTTTATTATAATTTTTTTTATAGTAATACCAATTTTAATT** |
| **Ceutorhynchus sp.*** | **GTCCTGAGCAGGAATAGCAGGAACTTCGTTAAGAATACTCATTCGAACCGAGTTAGGAAACCCAGGTAGCTTAATTGGTAATGACCAAATTTATAATTCAATTGTCACTGCTCATGCTTTTATTATAATTTTTTTTATAGTAATACCAATTTTAATT** |
| **Hypera meles** | **AACATGAGCAGGAACTGTTGGTACTAGCTTAAGAATTTTGATTCGTACAGAATTAGGAAATCCAGGATCTTTAATTGGAAATGACCAAATTTATAACACAATTGTGACAGCTCATGCATTCATTATAATTTTTTTCATAGTTATACCGATTATAATT** |
| **Dasytes plumbeus** | **AGCTTGATCAGGAATAGTAGGAATATCATTAAGATTACTAATTCGATCAGAATTAAATAACCCAGGAACATTAATTGGTAATGATCAAATTTATAATGTTATTGTAACTGCCCATGCATTCATTATAATTTTTTTTATAGTAATACCAATTTTAATT** |
| **Meligethes aeneus** | **AGCTTGATCTGGAATAGTAGGTACTTCTTTAAGTATATTAATTCGGACAGAATTAGGTAACCCGGGATCACTAATTGGAAATGACCAAATCTATAATGTTATTGTAACAGCCCATGCATTTGTTATAATTTTTTTTATAGTTATACCATTTATAATT** |
| **Meligethes carinulatus** | **GGCCTGATCAGGTATAGTAGGGACCTCATTAAGTATATTAATTCGAACAGAGTTAGGAAATCCAGGGTCATTGATTGGAAATGACCAAATTTACAATGTAATTGTTACAGCCCATGCCTTTGTTATAATTTTTTTTATAGTTATACCTTTTATAATC** |
| **Meligethes planiusculus** | **AGCATGATCTGGAATAATTGGAACATCTTTAAGAATATTAATTCGAACTGAATTAGGAAATCCTGGATCTTTAATTGGAAATGATCAAATCTATAATGTAATTGTAACAGCACATGCTTTTGTAATAATTTTTTTTATAGTAATACCATTTATAATC** |
| **Botanophila fugax DK*** | **AGCATGATCAGGAATAGTAGGAACTTCATTAAGTATTTTAATTCGAGCTGAATTAGGACACCCTGGAGCACTAATTGGAGATGATCAAATTTATAATGTTATTGTAACAGCACATGCTTTTATTATAATTTTTTTTATAGTAATACCTATTATAATT** |
| **Delia platura** | **AGCCTGGTCAGGAATAGTGGGAACCTCATTAAGTATTTTAATTCGAGCTGAATTAGGGAACCCTGGAGCATTAATTGGAGATGATCAAATTTATAATGTAATTGTAACAGCTCATGCTTTTATTATAATTTTCTTTATAGTAATACCTATTATAATT** |
| **Delia radicum** | **AGCATGATCAGGAATAGTAGGAACTTCATTAAGAATTTTAATTCGAGCCGAATTAGGACATCCTGGAGCATTAATTGGAGATGATCAAATTTATAACGTAATTGTAACAGCCCATGCTTTTATTATAATTTTTTTTATAGTAATACCTATTATAATT** |
| **Anthomyza collini DK*** | **AGCATGAGCAGGAATAGCAGGTACATCAATAAGAATTCTAATTCGAACAGAATTAGGTCACCCAGGTGCCTTAATTGGTGATGATCAGATTTATAATGTTATTGTAACTGCACATGCATTTGTTATAATTTTCTTTATAGTAATACCAATTATAATT** |
| **Anthomyza gracilis** | **AGCTTGAGCAGGAATAGCTGGTACATCAATAAGAATTCTTATTCGAACAGAATTGGGTCACCCCGGTGCCTTAATTGGTGATGACCAAATTTATAACGTTATTGTAACTGCACACGCATTTGTTATAATTTTCTTCATGGTAATACCCATTATAATT** |
| **Dilophus febrilis** | **AGCATGAGCAGGAATACTGGGAACATCTTTAAGAATATTAATTCGAGCCGAATTAGGCCATCCAGGAGCATTAATTGGGAATGATCAAATTTATAATGTAATTGTAACTGCCCATGCTTTTATTATAATTTTTTTTATAGTAATACCTATTATAATT** |
| **Angioneura acerba** | **AGCTTGATCAGGAATAATCGGAACTTCGTTAAGAATTTTAGTACGAGCTGAATTAGGTCATCCTGGTGCATTAATTGGAGATGACCAAATTTATAATGTAATTGTAACAGCTCATGCTTTTATTATAATTTTTTTTATAGTTATACCAATTATAATT** |
| **Bellardia sp.** | **GGCTTGATCTGGAATAATTGGAACTTCATTAAGTATATTAATTCGAGCTGAATTAGGACATCCTGGAGCATTAATTGGAGATGACCAAATTTATAATGTAATCGTTACAGCCCATGCATTTATTATAATTTTTTTTATGGTTATACCAATTATAATT** |
| **Lucilia caesar** | **AGCTTGATCCGGTATAATCGGAACTTCATTAAGAATTTTAATTCGAGCTGAATTAGGACACCCTGGTGCATTAATTGGAGATGACCAAATTTATAATGTAATTGTTACAGCTCATGCTTTTATTATAATTTTCTTTATAGTAATACCAATTATAATT** |
| **Lucilia sp.*** | **AGCTTGATCCGGAATAATTGGAACTTCTTTAAGAATTCTAATTCGAGCTGAATTAGGACATCCTGGAGCTTTAATTGGAGATGATCAAATTTATAATGTAATTGTTACAGCTCATGCTTTTATTATAATTTTTTTTATAGTAATGCCAATTATAATT** |
| **Melinda gentilis** | **AGCTTGATCAGGAATAATTGGAACTTCATTAAGAATTTTAGTACGAGCCGAGTTAGGACACCCCGGAGCATTAATTGGAGATGACCAAATTTATAATGTAATTGTCACAGCTCATGCTTTTATTATAATTTTTTTTATAGTAATACCAATTATAATC** |
| **Asteromyia sp.** | **GATTTGAGCAGGAATAGTAGGAACATCTTTAAGAATCTTAATTCGATTAGAATTAAGAACAATTTCTAATTTAATTGGTAATGATCAAATTTATAATGTTATTGTTACAGCTCATGCATTTATTATAATTTTTTTTATAGTTATACCTATTTTAATT** |
| **Cecidomyiidae sp.1** | **AATTTGATCAGGAATATTAGGAACTTCCTTAAGAATCTTAATTCGTTTAGAATTAAGAACTATCTCTAACTTAATTGGTAATGATCAAATTTATAATGTAATTGTTACTGCTCATGCTTTTATTATAATTTTTTTTATAGTAATGCCTATTTTAATT** |
| **Cecidomyiidae sp.2** | **AATTTGAGCTGGAATAATTGGAACTTCATTAAGAATTTTAATTCGATTAGAATTAAGAACTATTTCTAATTTAATTGGAAATGATCAAATTTATAATGTAATTGTAACAGCCCATGCTTTTATTATAATTTTTTTTATAGTTATACCGATTTTAATT** |
| **Cecidomyiidae sp.3** | **AATTTGAGCAGGTATAATTGGAACTTCATTAAGAATTTTAATTCGATTAGAATTAAGAACTATTTCCAATTTAATTGGAAATGACCAAATTTATAATGTTATCGTTACAGCTCATGCTTTTATTATAATTTTTTTTATAGTTATACCAATTATAATT** |
| **Cecidomyiidae sp.4** | **AATTTGAGCAGGAATAGTAGGAACATCATTAAGTTTATTAATTCGATTAGAATTAAGAACAATTAGAAATTTAATTGGAAATGATCAAATTTATAATGTTATTGTAACTGCACATGCGTTTATTATAATTTTTTTTATAGTTATACCTATTTTAATT** |
| **Cecidomyiidae sp.5** | **AATTTGAGCCGGAATAGTGGGAACTTCTTTAAGATTATTAATTCGAATAGAATTAAGAACTATTAGAAATTTAATTGGAAATGATCAAATTTATAATGTAATTGTTACAGCCCATGCTTTTATTATAATTTTTTTTATAGTTATACCAATTTTAATT** |
| **Cecidomyiidae sp.6** | **AATTTGATCAGGTATAGTAGGAACTTCATTAAGAATATTAATTCGATTAGAGTTAGGGGCTCCTGGAGCTTTAATTGGTAATGACCAAATTTATAATGTTATTGTAACTGCACATGCTTTTATTATAATTTTTTTTATAGTTATACCTATTATAATT** |
| **Cecidomyiidae sp.7** | **AATTTGATCTGGTTTAATTGGAACTTCTTTAAGAATATTAATTCGATTTGAATTAAGGACTACTAATGCTTTAATTGGTAATGATCAAATCTATAACGTTCTTGTCACCGCTCACGCTTTTATTATAATTTTTTTTATAGTTATGCCAATTATAATC** |
| **Cecidomyiidae sp.8** | **AATTTGAGCAGGAATATTAGGAACATCATTAAGAATATTAATTCGATTAGAATTAAGAACAATTTATAATTTAATTGGAAATGATCAAATTTATAATGTTATTGTTACTGCTCATGCTTTTATTATAATTTTTTTTATGGTTATACCAATTCTTATT** |
| **Ozirhincus longicollis** | **AATTTGATCAGGAATAGTAGGAACATCATTAAGAATTTTAATTCGAATAGAATTAAGAAGAATTTCTAATTTAATTGGAAATGATCAAATTTATAACGTTATTGTAACAGCTCATGCTTTTATTATAATTTTTTTTATAGTAATACCAATTTTAATT** |
| **Peromyia sp.** | **TATTTGATCTGGAATAGTAGGAACTTCTTTAAGTATATTAATTCGGTTTGAATTAGGAACAATTAATACTTTAATTGGTAATGATCAAATTTATAATGTAATTGTTACAGCTCATGCTTTTATTATAATTTTTTTTATAGTTATACCTATTATAATT** |
| **Rhopalomyia sp.** | **AATTTGATCAGGAATAATTGGAACATCATTAAGAATTATTATTCGAATAGAATTAAGAAGATTAAATAATTTAATTGGAAATGATCAAATTTATAATGTAATTGTAACTGCTCATGCTTTTATTATAATTTTTTTTATAGTAATACCAATTTTAATT** |
| **Culicoides impunctatus** | **AGCTTGAGCTGGAATAGTAGGAACATCTTTAAGTATTTTAATTCGTGCAGAACTTGGACACCCCGGGGCCTTAATTGGAAATGATCAAATTTATAACGTAATCGTAACCGCCCATGCTTTCGTAATGATTTTTTTTATAGTAATACCAATCATAATT** |
| **Dasyhelea sp.** | **AGCTTGGGCCGGAACAGTTGGAACTTCTTTAAGAATCCTAATCCGGGCCGAACTAGGCCACCCAGGAGCTCTTATCGGAAACGATCAAATTTATAACGTTATTGTAACAGCCCATGCATTTATTATAATTTTCTTTATAGTAATGCCAATTATAATT** |
| **Forcipomyia sp.** | **AGCTTGGGCCGGAATAGTGGGAACATCTTTAAGAATCCTAATTCGAGCTGAGTTAGGACATCCCGGTGCTTTAATTGGAGACGATCAAATTTATAACGTTATTGTTACCGCTCACGCATTCGTAATAATTTTTTTTATGGTAATGCCTATTATAATT** |
| **Leucopis sp.** | **AGCTTGATCAGGTATGGTTGGAACTTCACTAAGTATTTTAATTCGAATAGAATTAGGCCACCCAGGAGCTCTTATTGGAGATGATCAAATTTATAATGTAATTGTTACTGCCCACGCTTTTGTAATAATTTTCTTTATAGTTATACCAATTATAATT** |
| **Bryophaenocladius sp.** | **AGCTTGATCAGGTATAGTAGGTACTTCTTTAAGTATCCTTATTCGTGCAGAACTAGGACACGCTGGATCTTTAATTGGAGACGATCAAATTTATAATGTTATCGTAACAGCTCATGCTTTTGTTATAATTTTCTTCATAGTAATGCCTATTTTAATT** |
| **Cladotanytarsus sp.** | **AGCCTGATCAGGAATAGTGGGCACTTCTTTAAGAATACTAATCCGAGCCGAATTAGGGCATCCCGGAACCTTAATTGGAGATGATCAAATTTATAATGTTATTGTTACTGCACATGCTTTTATTATAATTTTTTTTATAGTTATACCTATTTTAATT** |
| **Corynoneura sp.** | **GGCCTGATCAGGAATGGTTGGAACTTCTCTAAGAATTCTAATTCGACTAGAATTAGGTCATTCAGGTTCATTAATTGGAGATGATCAAATTTATAATGTTATTGTAACAGCTCATGCTTTTGTAATAATTTTTTTTATAGTTATACCTATTTTAATT** |
| **Cricotopus ornatus** | **TGCCTGATCAGGGATAGTTGGTACTTCTCTAAGAATCTTAATTCGAGCTGAATTAGGTCACGCCGGATCATTAATTGGTGATGATCAAATTTATAATGTAATTGTTACAGCTCACGCTTTTGTTATAATTTTTTTTATAGTTATACCTATTTTAATT** |
| **Glyptotendipes sp.** | **TGCCTGATCTGGGATAGTCGGAACATCTCTAAGAATGCTTATTCGAGCAGAATTAGGACGACCTGGAACTTTTATCGGAGACGACCAAATTTATAACGTAATTGTTACAGCTCATGCTTTTATTATAATTTTTTTTATAGTTATACCTATTTTAATT** |
| **Guttipelopia guttipennis** | **AGCATGATCAGGAATAGTTGGTACTTCCCTTAGTATTTTAATTCGCACTGAACTTGGACACCCAGGAGCATTAATTGGAGATGATCAAATCTATAATGTAATTGTTACTGCACATGCTTTTGTAATAATTTTTTTTATAGTAATGCCTATTTTAATT** |
| **Halocladius variabilis** | **AGCATGATCTGGAATAGTAGGTACATCCCTTAGAATTTTAATTCGTGCTGAACTTGGACATGCCGGCTCTTTAATTGGAGACGACCAAATTTATAATGTAATTGTTACTGCTCATGCTTTTGTAATAATTTTTTTTATAGTTATACCTATTTTAATT** |
| **Halocladius varians** | **AGCTTGATCTGGAATAGTAGGCACTTCCCTTAGAATTTTAATTCGTGCCGAATTAGGTCATGCTGGTTCCTTGATTGGAGACGACCAAATTTATAATGTAATTGTTACAGCACATGCTTTTGTTATAATTTTTTTTATAGTTATACCAATTTTAATT** |
| **Metriocnemus fuscipes** | **GGCCTGATCAGGCATAGTAGGAACTTCCTTAAGAATTTTAATTCGAGCTGAATTAGGCCACGCCGGCTCTTTAATTGGAGATGACCAAATTTATAATGTAATTGTTACTGCTCATGCATTTATTATAATTTTTTTTATAGTAATACCGATTTTAATT** |
| **Micropsectra sp.** | **AGCTTGATCCGGAATAATTGGAACCTCATTAAGAATACTTATTCGAGCAGAATTAGGTCATCCAGGTACACTAATTGGAGATGATCAAATTTATAATGTAATTGTTACAGCTCACGCTTTTATTATAATTTTTTTTATAGTTATACCCATTTTAATT** |
| **Parakiefferiella coronata (perhaps new to DK?)** | **AGCTTGATCTGGCATAATCGGAACTTCTTTAAGAATTTTAATTCGAGCAGAATTAGGGCACGTAGGTTCTTTAATCGGAGACGACCAAATTTATAATGTTATCGTTACTGCTCATGCATTTATTATAATTTTTTTTATAGTTATACCTATTTTAATT** |
| **Pseudosmittia albipennis** | **AGCCTGATCCGGAATAATTGGGACTTCCCTTAGAATTTTAATTCGAGCTGAATTAGGACACTCAGGAGCATTAATTGGAGACGATCAAATTTATAATGTTATTGTTACAGCCCATGCCTTTGTAATAATTTTTTTTATAGTAATACCTATCTTAATT** |
| **Pseudosmittia trilobata (perhaps new to DK?)** | **AGCCTGATCTGGAATAGTAGGAACTTCTTTAAGAATTTTAATTCGAGCAGAACTAGGGCATTCGGGTGCTTTAATTGGAGATGACCAAATTTATAATGTTATTGTTACTGCTCATGCTTTTGTAATAATTTTTTTTATAGTTATACCTATTTTAATT** |
| **Smittia pratorum** | **GGCTTGATCAGGTATAGTTGGAACTTCTTTAAGAATTTTAATTCGGGCAGAATTAGGACATGCAGGTTCATTAATTGGTGATGATCAAATTTATAATGTAATTGTTACAGCTCATGCTTTTGTAATAATTTTTTTTATAGTAATACCAATTTTAATT** |
| **Smittia sp. (pratorum)** | **GGCTTGATCAGGTATAGTTGGAACTTCCTTAAGAATTTTAATTCGAGCAGAATTAGGACACGCAGGTTCATTAATTGGTGATGATCAAATTTATAATGTAATTGTTACAGCTCATGCTTTTGTAATAATTTTTTTTATAGTAATACCAATTTTAATT** |
| **Elachiptera cornuta DK*** | **GGCTTGAGCTGGAATAGTAGGAACTTCATTAAGAATTTTAATTCGAGCTGAATTAGGACATCCAGGAGCTTTAATTGGTGATGATCAAATCTATAATGTAATTGTTACTGCTCATGCTTTTGTAATAATTTTTTTTATAGTAATACCTATTATAATT** |
| **Meromyza sp.** | **TGCATGAGCAGGAATAGTAGGAACTTCATTAAGAATCTTAATTCGAGCAGAATTAGGTCACCCAGGTGCATTAATTGGAGACGATCAAATTTATAACGTAATTGTAACAGCACATGCATTTGTTATAATTTTTTTTATAGTTATACCTATTATAATT** |
| **Oscinella sp.** | **AGCGTGAGCCGGAATAGTAGGAACATCATTAAGAATTTTAATTCGAGCTGAATTAGGTCATCCAGGAGCATTAATTGGTGATGATCAAATTTATAATGTAATTGTTACTGCTCACGCTTTTGTAATAATTTTTTTTATAGTAATACCTATTATAATT** |
| **Siphonella oscinina** | **AGCTTGAGCTGGAATAGTAGGAACATCTTTAAGAATCTTAATTCGAGCAGAATTAGGACATCCTGGAGCCTTAATTGGTGATGATCAAATTTATAATGTAATTGTAACAGCACATGCATTTGTAATAATTTTTTTTATAGTAATACCTATTATAATT** |
| **Culex pipiens DK*** | **AGCTTGAGCTGGAATAGTTGGAACTTCTTTAAGTTTACTAATTCGAGCAGAATTAAGTCAACCAGGTGTATTTATTGGAAATGATCAAATTTATAATGTTATTGTAACTGCTCATGCTTTTATTATAATTTTTTTTATAGTAATACCAATCATAATT** |
| **Culex sp.** | **AGCTTGAGCTGGAATAGTAGGTACTTCTTTAAGTTTACTTATTCGAGCTGAATTAAGTCAACCTGGAGTATTTATTGGAAATGATCAAATTTATAATATTATTGTAACTGCACATGCATTTATTATAATTTTTTTTATAGTAATACCAATTATAATT** |
| **Ochlerotatus detritus** | **GGTTTGATCAGGAATAGTTGGTACATCTTTAAGTATTTTAATTCGAGCTGAATTAAGTCAACCAGGAATATTTATTGGTAATGATCAAATTTATAATGTAATTGTTACAGCACACGCTTTTATTATAATTTTCTTTATAGTAATACCTATCATAATT** |
| **Ochlerotatus sp*** | **AGTTTGATCAGGAATAGTTGGAACATCATTAAGAATTTTAATTCGTGCTGAATTAAGTCAACCAGGTATATTTATTGGAAATGACCAAATTTATAATGTAATTGTTACAGCTCATGCTTTTATTATAATTTTCTTTATAGTAATACCTATTATAATT** |
| **Chrysotus sp.** | **AGCATGAGCAGGAATAGTGGGAACATCTCTTAGTATTATTGTTCGAATAGAATTAGGACACCCAGGAGCACTAATTGGAGATGATCAAATTTATAATGTAATTGTTACAGCACATGCTTTTATTATAATTTTTTTTATAGTAATACCCATTATAATT** |
| **Dolichopus sp.** | **GGCTTGAGCAGGTATAGTGGGGACATCTCTTAGTATTATTGTTCGAGCAGAATTAGGTCATCCAGGAGCTCTAATTGGAGATGATCAAATCTATAATGTAGTAGTTACTGCCCATGCATTTGTTATAATTTTCTTTATAGTAATACCAATCATAATT** |
| **Drosophila fenestrarum** | **GGCATGAGCCGGTATAGTGGGAACATCATTAAGAATTTTAATTCGAGCTGAACTTGGTCACCCGGGAGCATTAATTGGAGATGATCAAATTTATAATGTGATCGTTACAGCTCATGCTTTTATTATAATTTTTTTTATAGTTATACCAATTATAATT** |
| **Scaptomyza sp.** | **AGCTTGAGCAGGAATAGTGGGAACATCTCTTAGTATTTTAATTCGAGCAGAACTGGGTCATCCTGGAGCTTTAATTGGAGATGATCAAATTTATAATGTAATTGTTACAGCACATGCTTTTGTAATAATTTTTTTTATAGTTATACCAATTATGATT** |
| **Fannia similis** | **TGCCTGATCTGGAATAATTGGTACTTCTTTAAGCATTTTAATTCGAGCTGAATTAGGTCATCCAGGAGCATTAATTGGTGATGATCAAATTTATAACGTTATTGTAACGGCTCATGCTTTTATTATAATTTTTTTTATAGTAATACCTATTATAATT** |
| **Helius longirostris** | **AGCTTGAGCAGGAATAGTTGGAACTTCTTTAAGTATATTAATTCGGGCAGAATTAGGTCATCCAGGGGCTTTAATTGGAGATGATCAAATTTATAATGTAATTGTTACTGCCCATGCATTTATTATAATTTTTTTTATAGTTATGCCTATTATAATT** |
| **Lonchoptera bifurcata** | **AGCTTGAGCAGGAATAGTAGGTACTTCTCTTAGAATTTTAATTCGAGCTGAATTAGGAAATCCAGGAGCTTTAATTGGTGATGATCAAATTTATAATGTGATTGTTACAGCTCATGCTTTTGTAATAATTTTTTTTATAGTTATACCTATTATAATT** |
| **Coenosia tigrina** | **AGCTTGATCTGGAATAGTAGGTACATCTTTAAGAATTTTAATTCGAGCTGAATTAGGTCATCCAGGAGCATTAATTGGTGATGATCAAATTTATAATGTAATTGTTACTGCTCATGCATTTATTATAATTTTTTTTATGGTAATACCAATTATAATT** |
| **Helina sp.** | **AGCTTGATCAGGAATAGTAGGAACTTCATTAAGTATTTTAATTCGAACTGAATTAGGTCATCCTGGTGCTTTAATTGGTAATGACCAAATTTATAATGTTATTGTAACAGCTCATGCTTTTATTATAATTTTTTTTATAGTAATACCAATTATAATC** |
| **Hydrotaea albipuncta** | **AGCATGATCTGGAATAGTAGGAACTTCTTTAAGAATTTTAATTCGAGCTGAATTAGGTCATCCAGGTGCATTAATTGGTGATGATCAAATTTATAATGTAATTGTTACAGCTCATGCATTTATTATAATTTTTTTTATAGTAATACCTATTATAATT** |
| **Morellia aenescens** | **AGCATGATCAGGAATAATCGGAACTTCATTAAGAATTTTAATTCGAGCTGAATTAGGACATCCTGGAGCCTTAATTGGTGATGATCAAATTTATAATGTAATTGTTACAGCTCACGCTTTTATTATAATTTTCTTTATAGTTATACCTATTATAATT** |
| **Morellia hortorum** | **AGCATGATCAGGTATAGTCGGAACTTCATTAAGAATTTTAATTCGAGCTGAACTAGGACATCCTGGAGCTTTAATTGGTGATGACCAAATTTATAATGTAATTGTTACAGCTCATGCTTTTATTATAATTTTCTTTATAGTTATACCTATTATAATT** |
| **Musca autumnalis** | **AGCATGATCTGGTATAATTGGAACTTCATTAAGAATTTTAATTCGAGCTGAATTAGGGCACCCTGGTGCACTAATTGGTGATGACCAAATTTATAATGTTATTGTAACAGCTCATGCTTTTATTATAATTTTCTTTATAGTTATACCTATTATAATT** |
| **Neomyia cornicina** | **AGCATGATCTGGAATAATTGGAACTTCCCTAAGAATTTTAATTCGTGCTGAATTAGGACATCCAGGAGCATTAATTGGTGACGACCAAATTTATAATGTAATTGTTACAGCTCATGCTTTTATTATAATTTTCTTTATAGTTATACCTATTATAATT** |
| **Phaonia tuguriorum** | **AGCCTGATCAGGAATAGTAGGAACTTCCCTAAGTATTCTTATTCGTGCTGAATTAGGACACCCTGATGCTTTAATTGGTGATGACCAAATTTATAACGTAATTGTAACAGCTCATGCTTTTGTAATGATTTTTTTTATAGTAATACCTATTATAATT** |
| **Polietes domitor** | **AGCATGATCTGGAATAATTGGAACTTCCTTAAGTATTTTAATTCGAGCAGAATTAGGACATCCTGGTGCACTAATTGGTGATGATCAAATTTATAATGTAATTGTTACTGCTCATGCTTTTATTATGATTTTTTTTATAGTTATACCTATTATAATT** |
| **Diptera sp.** | **AGCTTGAGCAGGAATAGTAGGAACTTCATTAAGAATTCTTATTCGAGCAGAATTAGGTCATCCTGGAGCATTAATTGGAGATGATCAAATTTATAATGTAATTGTTACAGCTCATGCTTTTATTATAATTTTTTTTATAGTAATACCTATTATAATT** |
| **Cephalops semifumosus DK*** | **TGCTTGAGCTGGAATAGTAGGAACTTCCTTAAGAATTTTAATTCGAGCAGAACTTGGACATCCAGGTTCTTTAATTGGAGATGATCAAATTTATAATGTTATTGTAACTGCCCATGCATTTGTAATAATTTTTTTTATAGTAATACCAATTATAATT** |
| **Psychoda sp.** | **TAGTTGAGCAAGAATAGTTGGTACTTCTTTAAGAATAATTATTCGAGCTGAATTAGGTCACCCAGGATCTTTAATTGGTAATGACCAAATTTATAATACTATTGTTACTGCACATGCTTTTGTTATAATTTTTTTTATAGTAATGCCTATTATAATT** |
| **Macronychia polyodon** | **AGCTTGATCAGGAATAGTAGGAACTTCTTTAAGAATTTTAGTACGAGCTGAACTAGGTCATCCCGGAGCTTTAATTGGAGATGATCAAATTTATAATGTAATTGTTACAGCTCATGCCTTTGTTATAATTTTCTTTATAGTAATGCCAATTATAATT** |
| **Sarcophaga sp.** | **AGCTTGAGCAGGTATAGTGGGAACTTCTCTAAGAATTCTTATTCGAGCAGAGTTAGGTCACCCTGGAGCATTAATTGGAGATGATCAAATTTATAACGTAATTGTTACAGCCCATGCTTTTATTATAATTTTCTTTATAGTAATACCAATTATAATT** |
| **Coboldia fuscipes** | **AGCTTGAGCTGGAATAGTGGGGACTTCTTTAAGAATTTTAGTTCGAGCCGAATTAGGACACCCAGGAGCATTAATTGGAGATGATCAAATTTACAATGTAATTGTTACTGCTCATGCCTTTGTTATAATTTTTTTTATAGTTATACCTATTATAATT** |
| **Scatopsidae sp.** | **AGCTTGAGCAGGAATAGTAGGGACATCCCTTAGAATCCTAGTCCGAGCCGAATTAGGTCATCCTGGTGCTTTAATTGGAGACGATCAAATTTATAATGTTATTGTAACAGCTCATGCTTTTGTAATAATTTTTTTTATAGTTATGCCTATTATAATT** |
| **Bradysia nitidicollis** | **TGCGTGATCAAGAATAGTGGGCACGTCACTTAGAATGCTAATTCGAGCAGAACTAGGGTGCCCTAATGCATTAATTGGGGACGATCAGATCTACAATGTAATTGTTACAGCACATGCATTTATTATAATTTTCTTTATAGTAATACCGATCATAATT** |
| **Hyperlasion wasmanni** | **AGCCTGATCGAGTATAGTAGGGACTTCCCTTAGACTTTTAATTCGAACGGAATTAGGAACACCTAATTCCTTAATTGGGAATGATCAAATTTATAATGTAATTGTTACAGCTCATGCTTTCATTATAATTTTTTTTATAGTTATACCTATTATAATC** |
| **Schwenckfeldina carbonaria** | **TGTTTGATCTAGAATAGTAGGTACTTCATTAAGAATATTAATTCGAGCAGAATTAGGATGCCCCAATGCTTTAATTGGTGACGACCAAATTTATAATGTAATTGTAACTGCCCATGCTTTTATTATAATTTTTTTTATAGTTATACCTATTATAATT** |
| **Sepsis cynipsea DK*** | **AGCTTGAGCAGGAATAGTAGGAACTTCATTAAGAATTTTAATTCGAGCTGAATTAGGACACCCTGGAGCTTTAATTGGTGATGACCAGATTTATAATGTAATTGTTACAGCTCATGCTTTTGTAATAATTTTCTTTATAGTTATGCCTATTATAATT** |
| **Eristalis pertinax** | **AACATGAGCAGGTATAGTAGGAACTTCATTAAGAATTTTAATTCGAGCTGAATTAGGTCATCCCGGAGCATTAATTGGTGATGACCAAATTTATAATGTTATTGTAACAGCTCATGCATTTGTAATAATTTTCTTTATAGTAATACCTATTATAATT** |
| **Melanostoma mellinum** | **AGCTTGAGCAGGTATAGTAGGAACATCATTAAGTATACTAATTCGTGCTGAACTTGGTCATCCAGGTGCTTTAATTGGAGATGACCAAATTTATAATGTAATTGTTACAGCTCATGCTTTTGTTATAATTTTTTTTATAGTTATACCAATTATAATT** |
| **Meliscaeva cinctella** | **AGCTTGAGCCGGAATAGTAGGTACTTCTTTAAGTGTATTAATTCGTGCTGAACTTGGACATCCAGGTGCTTTAATTGGAGATGATCAAATTTATAATGTAATTGTTACAGCTCATGCTTTTGTAATAATTTTTTTTATAGTAATACCTATTATAATT** |
| **Platycheirus clypeatus DK*** | **AACTTGAGCTGGAATAGTAGGAACTTCTTTAAGAATTTTAATTCGTGCAGAACTTGGTCATCCAGGAGCTTTGATTGGAGATGACCAAATTTATAATGTAATTGTTACAGCTCACGCTTTTGTTATAATTTTTTTTATAGTAATACCAATTATAATT** |
| **Sphaerophoria sp.** | **AGCTTGAGCTGGAATAGTAGGAACTTCTTTAAGTATTTTAATTCGTATAGAACTTGGTCATCCAGGAGCATTAATTGGAGATGACCAAATTTATAATGTAATTGTTACTGCACATGCTTTTGTTATAATTTTTTTTATAGTAATACCTATTATAATT** |
| **Syrphus ribesii** | **AACTTGAGCTGGTATAGTAGGAACATCTTTAAGTGTATTAATTCGTGCAGAACTTGGACACCCAGGTGCTTTAATTGGGGATGACCAAATTTATAATGTTATTGTAACTGCACATGCTTTTGTTATAATTTTTTTTATAGTAATACCAATTATAATT** |
| **Syrphus vitripennis DK*** | **AACTTGAGCTGGTATAGTAGGAACATCATTAAGTGTATTAATTCGTGCAGAACTTGGTCATCCAGGAGCTTTAATTGGAGATGATCAAATTTATAATGTTATTGTAACTGCTCATGCTTTTGTTATAATTTTTTTTATAGTAATACCAATTATAATT** |
| **Haematopota sp.** | **GACATGAGCCGGAATAATTGGAACCTCGCTAAGTATTTTAATTCGAGCTGAATTAGGACACCCGGGATCTTTAATTGGTGATGACCAAATTTATAATGTAATTGTAACAGCACATGCTTTTGTAATAATTTTCTTTATAGTTATACCTATTATAATT** |
| **Blondelia migripes** | **AGCTTGATCAGGAATAATTGGTACTTCACTAAGAATTTTAATTCGTGCTGAACTTGGTCATCCTGGTTCATTAATTGGAAATGATCAAATTTATAATGTAATTGTTACAGCTCATGCTTTTATTATAATTTTTTTTATAGTAATACCAATTATAATT** |
| **Dinera ferina** | **TGCATGATCTGGAATAATCGGTACATCATTAAGTATTTTAATTCGAACTGAATTAGGGCATCCAGAAGCCTTAATTGGAGATGATCAAATTTATAATGTAATTGTAACAGCACATGCTTTTATTATAATTTTCTTTATAGTAATACCAATTATAATT** |
| **Lydina aenea** | **AGCTTGAGCAGGAATAATTGGAACTTCTTTAAGTATTCTAATTCGAACTGAATTAGGTCATCCAGGTGCATTAATTGGAGATGATCAAATTTATAATGTAATTGTAACAGCCCATGCTTTTATTATAATTTTCTTTATAGTAATACCAATTATAATT** |
| **Phasia hemiptera** | **AGCTTGATCTGGAATAGTAGGAACTTCATTAAGTATTTTAATTCGAGCTGAATTAGGTCATCCTGGCTCACTAATTGGAGATGATCAAACATATAATGTAATTGTAACAGCTCATGCTTTTATTATAATTTTCTTTATAGTAATACCCATTATAATT** |
| **Phryxe vulgaris DK*** | **TGGTTGATCAAGAATAATTGGAACATCTTTAAGTATATTAATTCGAATAGAATTAGGTCATACAGGTTCATTAATTGGAAATGACCAAATTTATAATGTAATTGTTACAGCTCATGCATTTGTTATAATTTTTTTTATAGTAATACCAATTATAATT** |
| **Prosena siberita** | **AGCATGATCAGGAATAATAGGAACTTCATTAAGTATATTAATTCGAGCAGAATTAGGACATCCTGGAGCATTAATTGGTGATGACCAAATTTATAATGTAATTGTTACAGCTCATGCTTTTATTATAATTTTCTTTATAGTAATACCTATTATAATT** |
| **Siphona geniculata** | **AGCTTGAGCTGGTATAGTAGGAACGTCTTTAAGAATATTAATTCGAACCGAATTAGGTCATCCTGGATCATTAATTGGAGATGACCAAATTTATAATGTAATTGTAACAGCACATGCTTTTATTATAATTTTTTTTATGGTAATACCTGTAATAATT** |
| **Voria ruralis** | **AGCTTGATCAGGAATAGTAGGAACATCCCTTAGAATATTAATTCGATTTGAATTAGGTCATCCAGGAGCCTTAATTGGTAATGATCAAACATACAATGTTATCGTTACTGCTCATGCTTTTATTATAATTTTTTTTATAGTAATACCAATTATAATT** |
| **Cloeon dipterum** | **TGCATGAGCTGGTATAGTGGGCACTTCATTGAGTTTATTAATTCGTGCGGAACTAGGTAATCCTGGGTCTTTAATTGGTGACGACCAAATTTATAATGTTATTGTTACTGCTCACGCTTTTATTATGATTTTCTTTATGGTTATGCCAATTATGATT** |
| **Pineus sp.** | **AATATGATCAGGAATAATTGGATCATCATTAAGAATAATAATTCGAATAGAATTAAGACAAATTAACTCAATCATTAATAATAATCAACTTTATAATGTAATTATTACAATTCATGCATTTATTATAATTTTTTTTATAACAATACCTATTGTTATT** |
| **Orius laticollis** | **AATATGAGCAGGTATATTAGGTACATCATTAAGATGAATTATTCGAATTGAATTAGGACAGCCAGGATCATTTATTGGGGATGATCAAATTTATAATGTAGTAGTTACAGCTCACGCATTTATTATAATTTTCTTTATAGTTATACCAATTATAATT** |
| **Orius niger** | **AATATGAGCAGGCATATTAGGAACTTCACTAAGATGAATTATTCGGATTGAACTAGGACAGCCAGGGTCATTTATTGGAGATGACCAAATTTACAATGTAGTAGTTACAGCACACGCATTCATTATAATTTTCTTCATAGTTATACCAATTATAATT** |
| **Orius sp.** | **GATATGAGCAGGAATATTAGGAACATCATTAAGATGAATTATTCGAATTGAATTAGGACAGCCAGGATCATTTATTGGAGATGATCAAATTTATAATGTTGTAGTTACAGCACATGCATTTATTATGATTTTTTTTATAGTTATACCAATTATAATT** |
| **Aphis craccae** | **AATTTGATCAGGAATAATTGGATCTTCTCTTAGAATTCTAATTCGATTAGAATTAAGACAAATTAATTCAATTATTAATAACAATCAATTATATAATGTAATTGTTACAATTCATGCTTTCATTATAATTTTTTTTATAACTATACCAATTGTAATT** |
| **Aphis sp.** | **TATTTGATCAGGTATAATTGGATCTTCACTTAGAATTTTAATTCGATTAGAATTAAGACAAATTAATTCAATTATTAATAATAATCAACTATATAATGTAATTGTTACAATTCATGCTTTTATTATAATTTTTTTTATAACTATACCAATTGTAATT** |
| **Drepanosiphum platanoidis DK*** | **TATTTGATCAGGAATAATTGGATCATCATTAAGAATTTTAATTCGACTAGAACTTAGACAAATTAATTCAATTATTAATAATAATCAATTATATAATGTAATTGTTACAATTCATGCCTTTATTATAATTTTTTTTATAACTATACCTATTGTTATT** |
| **Euceraphis betulae** | **TATCTGATCAGGAATAATTGGATCATCATTAAGAATTTTAATTCGATTAGAGTTAAGACAAATTAACTCAATTATTAATAATAATCAATTATATAATGTAATTGTTACTATTCATGCTTTTATTATAATTTTCTTTATAACTATACCAATCGTAATT** |
| **Hyadaphis foeniculi DK*** | **AATTTGATCAGGTATAATTGGTTCATCTCTTAGAATTTTAATTCGTTTAGAATTAAGACAAATTAACTCTATTATTAATAATAATCAATTATATAATGTAATTGTTACAATTCATGCCTTTATTATAATTTTTTTCATAACAATACCAATTGTTATT** |
| **Hyalopterus pruni** | **TATTTGATCAGGTATAATTGGATCTTCTCTTAGAATATTAATTCGATTAGAACTTAGACAAATTAATTCAATTATTAATAATAATCAACTTTACAATGTGATTGTTACAATCCATGCTTTCATTATAATTTTCTTTATAACTATACCAATTGTAATT** |
| **Phyllaphis fagi** | **AATCTGATCAGGAATAATTGGATCATCATTAAGAATTTTAATTCGATTAGAATTAAGTCAAATTAATTCAATTATTAACAATAACCAATTATATAATGTAATTGTAACAATCCATGCATTTATTATAATTTTTTTTATAACTATACCAATTGTAATT** |
| **Schizaphis sp.** | **TATTTGATCAGGTATAATTGGGTCATCTCTTAGAATTTTAATTCGACTTGAATTAAGTCAAATTAATTCAATTATTAATAATAATCAACTATATAATGTAATTGTCACAATTCATGCTTTTATTATAATTTTTTTTATAACAATACCAATTGTAATT** |
| **Semiaphis dauci** | **AATCTGATCAGGTATAATTGGATCATCTCTTAGAATTTTAATTCGTCTAGAATTAAGTCAAATTAATTCAATCATTAATAACAATCAATTATATAATGTTATTGTAACAATTCATGCTTTTATTATAATTTTTTTTATAACAATACCAATTGTTATT** |
| **Philaenus spumarius** | **GATTTGATCTGGAATAATTGGGACTACTCTAAGATTATTAATTCGGGTTGAATTGGGTCAACCTGGGTCATTTATTGGGGATGATCAAATTTATAATGTAATTGTAACTTCCCATGCTTTTATCATGATTTTTTTTATAGTTATGCCAATTATAATT** |
| **Cinara pruinosa** | **GATTTGATCTGGTATAATTGGATCTTCACTTAGAATATTAATTCGTCTTGAATTAAGACAAATTAATTCAATTATCAATAATAATCAATTATATAATGTAATTGTTACAATTCATGCATTTATTATAATTTTTTTTATAACAATACCTATTGTAATT** |
| **Adelphocoris lineolatus** | **AATATGAGCAGGAATATTAGGAACATCATTAAGATGAATTATTCGAATCGAATTAGGAATACCAGGATCATTCATTGGGGATGATCAAACATATAATGTAGTAGTCACCGCACATGCATTTATCATAATCTTTTTTATAGTAATACCAATCATAATT** |
| **Lygus rugulipennis** | **AATATGAGCAGGAATATTAGGTACATCACTGAGATGAATTATTCGAATTGAATTAGGAATACCTGGATCATTTATTGGAGATGATCAAACATATAATGTTGTAGTTACAGCCCATGCTTTCATCATAATTTTCTTCATAGTTATACCAATCATAATT** |
| **Neolygus sp.** | **AATATGGGCAGGAATATTAGGTTCATCTTTAAGATGAATCATTCGAATTGAATTAGGTATACCAGGATCATTCATTGGTGATGATCAAACATATAATGTAGTAGTAACAGCTCATGCATTTATTATAATTTTCTTTATAGTTATACCAATTATAATT** |
| **Orthops basalis** | **AATATGGGCAGGAATACTAGGTACATCACTAAGATGAATTATTCGAATCGAATTAGGTATACCAGGATCATTTATTGGAGATGATCAAACATATAATGTAGTAGTTACAGCTCACGCTTTCATCATAATTTTTTTTATAGTAATACCAATCATAATT** |
| **Orthops campestris** | **AATATGAGCAGGAATACTAGGTACATCACTAAGATGAATTATCCGAATCGAATTAGGTATACCAGGATCATTTATTGGAGATGATCAAACATATAATGTAGTAGTTACAGCCCATGCTTTCATCATGATTTTCTTTATAGTGATACCAATTATAATT** |
| **Phytocoris varipes** | **ATTATGAGCAGGAATATTAGGAACATCATTAAGATGAATTATCCGTATTGAACTAGGAATACCAGGATCCTTTATCGGAGATGATCAAACATATAATGTAATTGTCACAGCTCATGCATTCATCATAATTTTTTTTATAGTTATACCAATTATAATT** |
| **Palomena prasina** | **AATATGGGCAGGAATAGTTGGATCAGCCATAAGAATAATTATCCGTATTGAATTAGGACAACCAGGAAGATTTATTGGAGATGATCAAATTTATAATGTTGTAGTAACAGCCCATGCTTTTATTATAATTTTTTTTATAGTAATACCCATTATAATT** |
| **Aphidius avenae** | **TATATGATCAGGAATAGTTGGGTTATCAATAAGATTAATTATTCGAATAGAATTAAGAATTACTGGTACTTTTATTGGTAATGATCAAATTTATAATAGTATTGTTACTGCACATGCTTTTGTAATAATTTTTTTTATAGTTATACCTATTATAATT** |
| **Lysiphlebus hirticornis** | **TATATGATCTGGTATATTAGGTTTATCTATAAGGTTAATTATTCGTATAGAATTAAGTGTAGCAGGAAGATTTATTGGAAGTGATCAAATTTATAATAGTATTGTTACAGCACATGCTTTTGTAATAATTTTTTTTATAGTTATGCCTATTATAATT** |
| **Praon sp. (perhaps new to DK?)** | **AATATGAGCAGGAATAGTAGGATTATCAATAAGTTTAATTATTCGATTAGAATTAGGTATACCTGGAAGATTAATTGGAAGAGATCAAATTTATAATAGAATTGTAACTTCACATGCTTTTGTAATAATTTTTTTTATAGTTATACCAATTATAATT** |
| **Promethes sulcator** | **TATATGAGCAGGTATAATTGGATCATCATTAAGAATAATTATTCGTATAGAACTTGGTAATCCAGGATATTTAATTAAAAATGACCAAATTTATAATTCTATTGTAACTTCACATGCTTTTGTAATAATTTTTTTTATAGTTATACCTGTAATAATT** |
| **Athalia rosae** | **ATTTTGATCAGGAATAATTGGATTATCAATAAGAATAATCATTCGAAGAGAATTAAGTTCAAGAAATTCTTTTATTAAAAATGATCAAATTTATAATACTATTGTTACATCACATGCTTTTCTAATAATTTTTTTTATAGTAATACCCATTATAATT** |
| **Dolerus germanicus** | **ATTCTGATCTGGAATATTAGGATTATCATTTAGAATAATAATTCGAACAGAATTAAGAATTCCAGGTTCTATTATTAATGATAATCAATTATATAATGTTATTGTAACATCACATGCATTTTTAATAATTTTTTTTATAGTAATACCAATTATAATT** |
| **Tenthredinidae sp.** | **ATTTTGATCTGGAATATTAGGATTATCATTCAGTATATTAATTCGAACAGAACTAGGAATACCCGGATCTATAATTGGTGATGACCAAATTTATAATGTAATTGTAACCTCCCACGCGTTCTTAATAATTTTTTTTATAGTTATACCAATCATAATT** |
| **Tenthredo sp.** | **ATTTTGATCAGGTATATTAGGACTATCATTTAGAATAATAATTCGAACAGAATTAGGAATACCAGGTTCAATAATTGGAGATGATCAAATTTATAATGTTATTGTAACATCACACGCATTTCTTATAATTTTTTTCATAGTTATACCAATTATAATT** |
| **Agriphila sp.** | **AATTTGAGCAGGAATAGTGGGAACATCTTTAAGATTATTAATTCGTGCTGAATTAGGAAATCCCGGTTGTTTAATTGGAGATGATCAAATTTATAATACTATTGTCACAGCTCATGCATTTATTATAATTTTTTTTATGGTTATACCTATTATAATT** |
| **Pleuroptya ruralis DK*** | **AATTTGAGCAGGAATAGTAGGTACCTCTTTAAGACTTTTAATTCGAGCTGAATTAGGAAGTCCAGGATCGTTAATTGGGGATGATCAAATCTATAATACAATTGTAACAGCTCATGCATTTATTATAATTTTTTTTATAGTAATACCAATTATAATT** |
| **Elachista nobilella** | **AATTTGAGCAGGAATAGTAGGAACTTCTTTAAGTTTATTAATTCGAGCAGAATTAGGGAATCCTGGCTCATTAATTGGAAATGATCAAATTTATAACACTATTGTCACAGCCCATGCATTTATTATAATTTTTTTTATAGTAATACCAATCATAATT** |
| **Eilema griseola** | **AGTTTGAGCTGGTATAGTAGGAACTTCACTCAGACTTTTAATTCGAGCAGAATTAGGAAATCCAGGATCTTTAATTGGTGATGATCAAATTTATAATACTATTGTAACTGCTCATGCTTTTATCATAATTTTTTTTATGGTTATACCTATTATAATT** |
| **Aproaerema anthyllidella** | **TATTTGAGCAGGAATAGTCGGAACATCTCTTAGTTTATTAATTCGAGCAGAATTAGGAAACCCGGGTCAATTAATCGGAGATGATCAAATTTATAATACTATTGTAACAGCTCATGCCTTTATTATAATTTTTTTTATAGTTATGCCAATTATAATC** |
| **Isophrictis striatella** | **GGTCTGAGCAGGAATAGTTGGAACTTCTTTAAGTTTATTAATTCGAGCTGAATTAGGAACTCCAGGATCTTTAATTGGAGATGATCAAATTTATAATACTATTGTAACTGCCCATGCTTTTATTATAATTTTTTTTATAGTTATACCTATTATAATT** |
| **Acasis viretata** | **AATTTGGGCAGGAATAGTAGGGACTTCTCTTAGATTATTAATTCGAGCAGAATTAGGAAACCCTGGGTCTTTAATTGGGGATGATCAAATTTATAACACTATTGTCACAGCTCATGCTTTTATTATAATTTTTTTTATAGTAATGCCTATTATAATT** |
| **Chloroclystis v-ata** | **AATTTGAGCTGGTATAATTGGAACTTCATTAAGATTACTAATTCGAGCAGAATTAGGAACCCCCGGATCTTTAATTGGAGATGACCAAATTTATAATACTATTGTTACAGCTCATGCTTTTATTATAATTTTTTTCATAGTTATACCTATTATAATT** |
| **Eupithecia absinthiata DK*** | **AATTTGAGCAGGTATAATTGGAACTTCATTAAGATTGCTAATTCGAGCAGAATTAGGAACCCCCGGATCTTTAATTGAAGATGATCAAATTTATAATACTATTGTTACAGCTCATGCTTTTATTATAATTTTTTTTATAGTAATACCTATTATAATT** |
| **Eupithecia tripunctaria** | **AATTTGAGCTGGAATAATTGGAACTTCTTTAAGATTATTAATTCGAGCTGAATTAGGAACTCCAGGATCCTTAATTGGGGATGATCAAATTTATAATACTATCGTAACAGCTCATGCCTTCATTATAATTTTTTTTATGGTAATACCAATTATAATC** |
| **Eupithecia virgaureata DK*** | **AATTTGAGCTGGAATAATCGGAACTTCCCTAAGATTATTAATTCGAGCTGAATTAGGAACTCCAGGTTCTTTAATCGGAGATGATCAAATTTATAATACTATTGTTACAGCTCATGCTTTTATTATAATTTTTTTTATAGTAATACCGATTATAATC** |
| **Scotopteryx chenopodiata** | **TGTTTGAGCTGGGATAGTTGGAACTTCCTTAAGCCTACTAATCCGAGCTGAATTAGGAACCCCAGGATCTTTAATTGGAGATGATCAAATTTATAACACTATTGTAACAGCTCATGCTTTCATTATAATCTTTTTCATAGTAATACCAATTATAATT** |
| **Thymelicus lineola** | **AATTTGAGCAGGAATATTAGGTACTTCTTTAAGTTTATTAATTCGAACAGAATTAGGAAACCCAGGATCATTAATTGGAGATGATCAAATTTATAATACTATTGTTACAGCTCATGCTTTTATTATAATTTTTTTTATAGTAATACCTATTATAATT** |
| **Mompha epilobiella** | **TATTTGAGCAGGAATGGTTGGAACCTCTTTAAGATTACTAATTCGAGCAGAATTAGGAAACCCCGGATCTTTAATTGGCGATGATCAAATTTATAATACAATTGTAACAGCTCATGCATTTATTATAATTTTCTTTATAGTAATACCAATTATAATT** |
| **Lepidoptera sp.1** | **AATTTGATCAGGAATAGTAGGAACTTCTTTAAGACTACTAATTCGAGCAGAATTAGGAAACCCAGGATCTTTAATTGGAGATGATCAAATTTATAATACTATTGTAACAGCTCATGCTTTTATTATAATTTTTTTTATAGTTATACCAATTATAATT** |
| **Lepidoptera sp.2** | **AATTTGAGCAGGAATAGTTGGAACTTCTTTAAGTTTATTTATTCAAGCTGCATTAGGGACTCTAGGATCTTTAATTGGAGATGATCAAATTTATAATACTATTGTAACTGCCCATGCTTTTATTATAATTTTTTTTATAGTTATACCTATTATAATT** |
| **Lepidoptera sp.3** | **AATTTGAGCTGGTATAATTGGAACTTCATTAAGATTACTAATTCGAGCAGAATTAGGAACCCCCGGATCTTTAATTGGAGATGACCAAATTTATAATACTATTGTTACAGCTCATGCTTTTATTATAATTTTTTTTATAGTAATACCTATTATAATT** |
| **Lepidoptera sp.4** | **AATTTGAGCAGGTATAATTGGAACTTCATTAAGATTGCTAATTCGAGCAGAATTAGGAACCCCCGGATCTTTAATTGAAGATGATCAAATTTATAATACTATTGTTACAGCTCATGCTTTTATTATAATTTTTTTTATAGTAATACCTATTATAATTGGAGGATTTGGAAATTGATTAGTAAG** |
| **Lepidoptera sp.5** | **AATTTGAGCTGGAATAATCGGAACTTCCCTAAGATTATTAATTCGAGCTGAATTAGGAACTCCAGGTTCTTTAATCGGAGATGATCAAATTTATAATACTATTGTTACAGCTCATGCTTTTATTATAATTTTTTTTATAGTAATACCTATTATAATT** |
| **Lepidoptera sp.6** | **AATTTGAGCAGGTATAATTGGAACTTCATTAAGATTGCTAATTCGAGCAGAATTAGGAACCCCCGGATCTTTAATTGGAGATGATCAAATTTATAATACTATTGTTACAGCTCATGCTTTTATTATAATTTTTTTCATAGTTATACCTATTATAATT** |
| **Lepidoptera sp.7** | **GATTTGAGCAGGAATAGTTGGAACATCTTTAAGATTACTAATTCGAGCAGAATTAGGAACTCCAGGGTCATTAATTGGAGATGATCAAATTTATAATACTATTGTAACTGCCCATGCTTTTATTATAATTTTTTTTATAGTTATACCTATTATAATT** |
| **Apamea anceps** | **AATTTGAGCTGGTATAGTAGGAACTTCCCTCAGATTATTAATTCGAGCTGAATTAGGAAATCCAGGATCCTTAATTGGCGATGATCAAATTTATAATACTATTGTTACAGCTCATGCTTTTATTATAATTTTTTTTATGGTTATACCTATTATAATT** |
| **Apamea sordens** | **GATTTGAGCAGGTATAGTAGGAACTTCTTTAAGATTAATAATTCGAGCCGAATTAGGAAATCCCGGATCTTTAATTGGTGATGATCAAATTTATAATACTATTGTCACAGCTCATGCTTTCATTATAATTTTTTTTATAGTTATACCTATTATAATT** |
| **Autographa gamma** | **TATTTGAGCTGGAATAGTTGGTACATCTTTAAGATTACTAATTCGAGCAGAATTAGGAACCCCTGGATCTTTAATTGGTGATGATCAAATTTATAATACTATTGTTACAGCTCATGCATTTATTATAATTTTTTTTATAGTTATGCCTATTATAATT** |
| **Mythimna sp.** | **AATTTGAGCTGGAATAGTTGGAACTTCATTAAGATTACTAATTCGAGCTGAATTAGGAACTCCTGGATCTTTAATTGGAGATGATCAAATTTATAATACTATTGTAACAGCCCATGCTTTTATTATAATTTTTTTTATAGTTATACCCATTATAATT** |
| **Phlogophora meticulosa** | **AATTTGAGCCGGTATAGTAGGTACTTCATTAAGATTATTAATTCGAGCTGAATTAGGAAATCCTGGATCATTAATTGGAGATGATCAAATTTATAACACTATTGTTACAGCCCATGCTTTCATTATAATTTTTTTTATAGTTATACCTATTATAATT** |
| **Aphantopus hyperantus** | **AATTTGAGCAGGAATAGTAGGAACCTCCCTTAGACTTATTATTCGAACAGAATTAGGTAATCCTGGATTTTTAATTGGAGATGATCAAATTTATAATACTATTGTTACAGCTCATGCCTTTATTATAATTTTTTTTATAGTAATACCTATTATAATT** |
| **Hofmannophila pseudospretella** | **TATTTGAGCAGGCATAGTAGGAACGTCTTTAAGATTATTAATTCGAGCTGAATTAGGAAACCCAGGATCTTTAATCGGAGATGATCAAATTTATAATACTATTGTAACAGCTCATGCTTTTATTATAATTTTTTTTATAGTTATACCTATTATAATT** |
| **Dahlica sp.** | **AGTATGATCAGGAGTATTAGGAACATCTTTAAGAATACTAATTCGAACAGAATTAGGAATCCCTCAATCTTTAATCGGAGATGATCAAATTTATAATACTATTGTAACTGCTCATGCTTTTATTATAATTTTTTTTATAGTAATACCAATTATAATT** |
| **Gillmeria ochrodactyla** | **GATTTGAGCAGGAATAGTTGGAACATCTTTAAGATTACTAATTCGAGCAGAATTAGGAACTCCAGGGTCATTAATTGGAGATGATCAAATTTATAATTCAATTGTTACAGCCCATGCATTCATTATAATTTTTTTTATAGTTATACCTATTATAATT** |
| **Dichrorampha obscuratana** | **TATTTGATCTGGTATAATTGGAACTTCTTTAAGATTACTAATTCGTGCTGAATTAGGGAATCCTGGATCTTTAATTGGAGATGATCAAATTTATAACACTATTGTTACAGCACATGCTTTTATTATAATTTTTTTTATAGTTATACCTATTATAATT** |
| **Eucosma cana** | **TATTTGAGCTGGCATAATTGGAACTTCTTTAAGATTAATAATTCGAGCAGAATTAGGAAATCCAGGATCTTTAATTGGAGATGATCAAATTTATAATACAATTGTTACTGCTCATGCTTTTATTATAATTTTTTTTATAGTAATACCTATTATAATT** |
| **Eucosma hohenwartiana/fulvana/parvulana complex** | **TATTTGATCTGGAATAGTTGGAACATCATTAAGATTATTAATTCGAGCAGAACTAGGAAATCCTGGATACTTAATTGGTGATGATCAAATTTATAACACAATTGTTACTGCCCATGCCTTTATTATAATTTTTTTTATAGTTATACCTATTATAATT** |
| **Micromus variegatus** | **GATTTGATCAGGTTTAGTAGGAACAAGATTAAGTTTATTAATTCGAGCTGAATTAGGTCAACCTGGTTCTTTAATTGGAGACGACCAAATTTATAATGTAATTGTAACAGCTCATGCTTTTATTATAATTTTTTTTATAGTTATACCTATTGTAATT** |
| **Leptophyes punctatissima** | **CGCCTGAGCAGGAATAGTTGGTACATCTTTAAGTTTACTAATTCGTGCTGAACTAGGACAACCTGGCTACTTAATTGGTGATGATCAAATTTATAATGTTATTGTTACTGCCCATGCATTTGTAATAATTTTCTTCATAGTTATACCTATTATAATT** |
| **Valenzuela flavidus** | **TATTTGAGCTGGTATAGTAGGAACAAGATTAAGTATTTTAATTCGTATCGAACTAGGCCAACCTGGCCTTTTCTTAGAAGATGACCAAACTTATAATGTTATTGTAACAGCTCACGCTTTTATTATAATTTTCTTCATAATTATACCAATCATAATT** |
| **Ectopsocus briggsi** | **AATTTGATCTGGTATGGTAGGAACTAGCTTAAGTGTTCTAATCCGATTAGAATTAGGTCAACCTGGGTTATTTCTAGAAGATGACCAAACTTATAATGTTATTGTAACAGCACATGCTTTTATTATAATTTTTTTTATAATTATACCTATTATAATT** |
| **Lachesilla pedicularia** | **AATTTGGGCAGGAATAGTAGGTACTAGACTAAGAGTTTTAATTCGCTTAGAATTAAGACAACCAGGCTTATTCCTTGAAGATGACCAAACATACAATGTAATAGTAACAGCTCATGCTTTTATTATAATTTTCTTTATAGTAATACCTATTATAATT** |
| **Peripsocus subfasciatus** | **AATTTGAGCTGGTATACTTGGGACTAGTTTAAGAATCTTAATTCGACTTGAATTAGCCCAACCAGGCTTATTTTTAGAAGATGACCAAACATATAATGTTATCGTTACCGCTCACGCTTTTATTATAATTTTTTTTATAGTAATACCAATTATAATT** |
| **Aeolothrips fasciatus** | **GGCCTGATCTGGGATACTAGGTTTATCCTTAAGTATAATTATCCGAATTAACCTGCGAAATAATATAAAAGTATTAATTGATGATGACCAATTTTATAATTCAGTTGTTACAGCCCATGCTTTTGTTATAATTTTTTTTACAGTTATACCTATTCTTATC** |
| **Thrips major** | **ATTTTGATCAGGATTAATAGGACTTTCATTAAGAATAATTATTCGTTTAAACTTACGTACATCAATAAAATTATTTGTAAGAAATGATCAATTCTATAATTCAATTGTAACAGCACATGCATTTATTATAATTTTTTTCACAGTTATACCAATTATAATT** |
| **Thrips tabaci** | **ATTTTGGTCAGGAATGATAGGGCTTTCTTTAAGAATAATTATTCGATTAAATTTACGAACATCAATAAAACTATTCATTAGAAACGATCAATTTTACAATTCAATTGTTACGGCTCACGCTTTTGTAATAATTTTTTTTACAGTTATACCTATTATAATT** |
| **Asellus aquaticus** | **TGCATGATCAGGCAGGGTAGGAACTGCTCTTAGTATATTAATTCGAACAGAACTGGGACAACCTGGTAGATTTATTGGCAATGACCAGATTTACAATGTAATTGTAACTGCTCATGCTTTTGTTATAATTTTTTTCTTAGTTATACCAGTGATAATC** |
| **Philoscia muscorum** | **GGCGTGGTCGGGCGCGGTAGGTATAGCTTTAAGCATAATTATCCGAACAGAGCTAGGTCATGCCGGGAGATTAATTGGAGACGATCAAATTTATAATGTAATTGTTACTGCACATGCTTTTGTAATAATTTTCTTTATAGTGATACCTATTATAATT** |
| **Deroceras agreste** | **GGTTTGATGTGGAATAGTAGGGACTGGCCTTTCGCTTTTAATTCGACTGGAATTAGGAACAGCGGGAGTTTTACTAGATAATCATTTTTTTAATGTAGTAGTAACTGCTCATGCATTTGTTATGATTTTTTTTATGGTAATACCTATCATAATT** |
| **Deroceras invadens** | **GGTTTGATGTGGTATAGTTGGAACTGGGCTTTCATTACTAATTCGATTAGAATTAGGGACAGCTGGGGTATTATTAGATGACCATTTTTTTAATGTTATTGTGACAGCTCATGCATTTGTTATGATTTTTTTTATAGTAATACCAATTATAATT** |
| **Arion cf. vulgaris** | **GATTTGAAGAGGATTGGTTGGTACAGGTCTATCTCTTTTAATTCGCTTAGAGTTAGGAACAACTGGTATTTTAACTGATGATCAATTTTTTAATGTTATTATCACTGCACACGCTTTTGTAATAATTTTTTTCATAGTTATACCACTAATAATT** |
| **Fruticicola fruticum** | **CGTATGATGTGGTATAGTAGGTACGGGCCTCAGGTTGCTGATTCGATTAGAGCTCGGGAGTACGGGAGTATTGAGGGACGAGCATTTCTACAATGTTATTGTTACCGCCCATGCATTTGTTATAATTTTTTTCATAGTAATACCAATTATAATT** |
| **Steinernema feltiae** | **TTTATGATCTGGTATAGTAGGTACAAGACTTTCTTTAATTATTCGTCTTGAACTTGCTAAACCTGGTTTATTTTTAGGTAATGGTCAACTTTATAACTCTGTTATTACTGCTCATGCTATTCTTATAATTTTTTTTATAGTAATACCTAGTATAATT** |
| **Adineta vaga** | **CATTTGATCTGGTTTTTTAGGTGCTAGAATAAGTTTGATTATTCGTACTGAGTTAGGGATAGTTGGAAGAATTATTATAGATGAGCAAATTTATAATTCTATGGTTACAGCTCATGCTTTTTTAATGATTTTCTTTTTTGTTATACCCGTTGCTGTA** |
| **Habrotrocha elusa** | **TGTTTGATCTGGTTTTTTGGGTGCAAGAATTAGGTTAATTATTCGTACTGAATTAGGAATAGTAGGAAGTATTATTATAGATGAGCAAATTTATAATTCTATAGTTACGGCTCATGCTTTTTTAATAATTTTTTTTTTTGTGATGCCTGTGGCTGTT** |
| **Albugo candida** | **TGCTTTTTCAGGATTAGTAGGTACAACACTTTCTATTTTAATTCGTATGGAATTATCACAACCTGGTAATCAGATTTTTATGGGTAATCATCAATTATATAATGTAGTTGTAACAGCACATGCGTTCGTTATGATTTTTTTTATGGTTATGCCTGTTTTAATT** |
| **Peronospora ervi** | **CGCTTTTGCAGGTATAATTGGTACAACACTTTCAATGTTAATTCGAATTGAATTGTCACAACCTGGTAATCAAATTTTTATGGGAAATCATCAATTATATAATGTTGTTGTTACCTCACATGCTTTTATTATGGTTTTTTTTTTAGTTATGCCTGCTTTAATT** |
| **Peronospora obovata** | **TGCTTTTGCTGGTATTATCGGTACAACACTTTCTATGTTAATTAGAATTGAATTATCACAACCAGGTAATCAAATTTTTATGGGAAATCATCAATTATATAATGTTGTAGTTACTGCTCACGCTTTTATTATGGTTTTTTTTTTAGTTATGCCTGCTTTAATT** |
| **Peronospora radii** | **TGCTTTTTCTGGTATTATTGGTACAACACTTTCTATGTTAATTCGAATCGAATTATCACAACCAGGTAATCAAATTTTTATGGGAAATCATCAATTATATAATGTAGTTGTTACTGCTCATGCCTTTATTATGGTTTTTTTTTTAGTTATGCCTGCCTTAATT** |
| **Peronospora trifolii-minoris** | **TGCTTTTTCAGGTATTATCGGCACAACACTTTCTATGTTAATTAGAATCGAATTATCACAACCTGGAAATCAAATTTTTATGGGAAATCATCAATTATATAATGTTGTTGTAACTGCACATGCTTTTATTATGGTTTTTTTTTTAGTTATGCCTGCTTTAATT** |
| **Peronospora trifoliorum** | **TGCTTTTGCAGGTATTGTCGGTACAACACTTTCGATGTTAATTAGAATAGAATTATCACAACCCGGAAATCAAATTTTTATGGGAAATCATCAATTATATAATGTTGTTGTAACTGCACATGCTTTTATTATGGTTTTTTTTTTAGTTATGCCTGCTTTAATT** |
|  |  |
| **16S** | |
| **Leiobunum rotundum** | **GCTTTACTTAATTATAGTTTAAGATTTTTTCTAACTTAATTTATAATTTAAGTTTTACTGGGGCGGTAGCTATTAAACATAGTTGGTTTAATAAACAAGATCCAATATTATTGAAGTAAGATTTAGT** |
| **Willowsia nigromaculata** | **GCTTAATGCTTCTTTTAAACCATTCTTTGGGGGAAAGAATGGTTTAATAGGAATGTGTTTAGTTGGGGCAATTTTAGAATAATAAAAACTTCTAATGATTTTAATAAATCTTAAATTTTAAAAACTAAAAAGT** |
| **Isotoma viridis** | **TTTTTATTTTAGGCTGGTTTGATTTGTTAGAGTTTTATTCAAAATAAGTTTAAAAATTTTGTTGGGGCAACAAAAGAATATAATTAACTTCTTTCGTTTAATTTTTATTAAATTGTTAATCTTTTAGTAAAAAAGTTAAAAAAAGT** |
| **Apion fulvipes** | **GTTTAATAGTTTAATTAATTTAAATTAATTAGAATTAATTTAATGAAAATTAATTAATTATTTGATTGGGGTGATTAAAAAATTTAATTAACTTTTTTTGTATTTTTACATTAATTAATGAAAATATGATCCTTTTTATAAGATTAAAAGAATAAAT** |
| **Carabus nemoralis DK*** | **TCTTTATAATTTGTTAATTAAAGTTAATTTAGAAATATTAATGTTTTATTTAATTAAATTATTTTATTGGGGTAATAGAAAGATTGAATTAATTCTTTTTTTTAATAAACATTAATTTATGGATGTTAGATGATCCAGTTTTACTGATTATAAGATTAAGT** |
| **Coccinella septempunctata** | **GTTTTATTATATTAATTTTTTAAATTTTTAGAATTTAAAAATTTTAATTTTTAATATAATTTGGTTGGGGTGACTGAAAAATTAAATAAACTTTTTTTATATTATTACATAGATTTATGAATATTTGATCCATAAAAATGATTAAAAGAATAAAT** |
| **Harmonia axyridis** | **GTTTAATTTATAAAAGATTTTTAATTTAAAGAATTATAAAATTTAAAATTTTTATTAAATTTGATTGGGGTGATAAAAAAATTAGATTAACTTTTTTAACTTTAACCATTAATTTATGAAAAATTGATCCATTTTTATGATTAAAAGAATAAAT** |
| **Tytthaspis sedecimpunctata** | **GTTTAATTTTTAAAAGTTAATAAATTTTTAGAATTTTTTGATTTCTTGATTTTTAAGAATTTGATTGGGGTGATTGAAAAATTAATATAACTTTTTTTTTATTAATACATTAATTTATGAATATTTGATCCAATAAATTGATTATAAGAATAAAT** |
| **Tychius picirostris** | **GTTTTATATTTGATTATTATAGGTTTTTTAGAATTTTAATCTTTATGATATTTAAATATTTAGTTGGGGTGATTGAAAAATTAAATTAACTTTTTTTTTATTATTACATTTATTTATGAATAATTGATCCTTAATTAAAGATTATAAGATAAAAT** |
| **Trogoderma sp.** | **GTTTTATTGTTGTATTTGTAAGGTTTTGGTATTTAGACTTTCAATGTTAGAAACAATTTAGTTGGGGTGACTGGAAAATTGATTGAACTTTTCCTTTATTTTTACATTTATTTATGATTATATGATCCACGGATTGTGATTATAAGATATAAT** |
| **Meligethes planiusculus** | **GTTTTATAAAAATTATTTTTTTCTTATTTTTAGTATTAATAAAATGAAAAATTTATTTTTATTTGGTTGGGGTGATTAAAAAATTTAAAAAACTTTTTTTTATTTAGTACATTTATTTATGAATATTTGATCCATATATTATGATTAAAAGAAAAAAT** |
| **Meligethes sp. (aeneus)** | **GTTTTATAAAGAATTTGTTTTTGATTTTTTAGAATTAATTATAATGAGGCAATGTTTTATTTGGTTGGGGCGATTAAAAAATTTAATAAACTTTTTTTCTTATTTTACATTAATTTATGAATACTTGATCCTTAATTTAAGATTATAAGATTAAAT** |
| **Stilbus testaceus** | **GTTTAATAGAAAAGGAATAATTTATTTTTTAGTATTAATAAATTAATTATTTTTTTATATTTAATTGGGGTGATTAAAAAATTTAATAAACTTTTTTTATTTTAAAACAATAATATTTGAATTATTGATCCAAAATTTTTGATTATAAGAATAAAT** |
| **Forficula auricularia** | **TTTTCACTACAAACCCAATTTCTAGGGAGGGAAGGTTAATCTTTAAGAGATTGAAAGGTATTTTGGTTGGGGCGACAGGAAAAGAGATTAAGCTTTTCTTTTAAGGCAATACAAATATGTAAACGAAGGATCCAAATTGATTGGCAAGAAGAAGAAAA** |
| **Lucilia caesar** | **TCTTTATATTTATATTATTATAATTTTGTAGATTTTTTTTGTTATGATAATAGATGATATTTTATTGGGGTGATATTAAAATTTAATAAACTTTTAATTGTTTTAAATCATTAATTTATGAATAATTGATCCGTTATTAGCGATTAAAAAAACAAGT** |
| **Pollenia rudis** | **TCTTTATATTTAGGTTATTATAATTTTATAGAAGATTTATATTATAATAATTAATAATATTTTATTGGGGTGATATTAAAATTTAATAAACTTTTAATTTTTTTTACCATTAATTTATGAATATTTGATCCGTTATTAACGATTAAAAAAATAAGT** |
| **Culex sp.** | **TCTTTATTTTTGTTATTTATAAATTAAAAAGAATTTTAAAATTTATAATTTAATAAAAAATTTTATTGGGGTGATATTAAAATTTAAAAAACTTTTAAAATTTATTAACATAAATATATGAATAAATGATCCAGTTTTATTGATTAAAAATTTAAGT** |
| **Hercostomus sp.** | **TCTTTATATTTTATTTATTAATTTTTTTAGGTTATTTTTTAATTTAATAAATAGAATTATTTTGTTGGGGTGACAATGAAATTTAATAAACTTTCATTATTTTATAACATTAATTTATGAATAATTGATCCATTATTATTGATTAAAAATTTAAGT** |
| **Medetera truncorum** | **TCTTTATAATAAAAAATTTATTAGTTTATAGGATTTATTTTACAATAATTTTTTTATTATTTTATTGGGGTGATATTGAAATTTGATAAACTTTTGATTAATATTTATTCCGTTGATTTATGAATAAATGATCCATTTTTAGTGATTAAAAATTTAAGT** |
| **Musca autumnalis** | **TCTTTATATTTAGACAATTATAATTTTTCGGATTTTTTTTGTTATGATTGTTAATAATATTTTATTGGGGTGATATTAAAATTTAAAAAACTTTTAATTAATTAAATCATTAATTAATGATTAATTGATCCGTTATTAACGATTAAAAATTTAAGT** |
| **Opomyza florum** | **TCTTTATTCTTACGGTAGTTTATTTATTTAGAAGAATTTTGGTATAATATTGTTAAGTATTTTATTGGGGTGATATTAAAATTTAATAAACTTTTAATTTTGTTATTACATTGATTTATGAATAAATGATCCATTTTTATTGATTATAAATTTAAGT** |
| **Schwenckfeldina carbonaria** | **GTTTTATACAAAAATTTAAATTTAATTATAAGATTAATTAATTTTTTTATCTTCTTTGTATTTTGTTGGGGTGATAGTAAAATTTATAAAACTTTTATTTTTAAAAAACATTAATTTATGAATAATTGATCCATTTTTAATGATTAAAAAATTAAAT** |
| **Cloeon dipterum** | **TCTTAATAATAAACCCGCTATCTAATTTGGAATTTTAATTACTTATTAAGGTTAAGTTATTTGGTTGGGGCGACGAGAAGTTAATTAACGCTTCAAATTTAAACAACAGTATAAGGTTAAATGACCCGGAGTCCGATTATAAATACAAGT** |
| **Aphididae sp.** | **ACTTTATAAATATAAATTTTAATTTTGTTATTTATTTTATTGGGGTGATAAAAAAAATTATTAAACTTTTTTTAAAATTTTACATTATTTAGTGAGTATTTGAATTAAAATTTTTAATTATAGGAAAAAGT** |
| **Adelphocoris lineolatus** | **ACTTTACTTTAATATAAGTATACTATTTATATATTTTTAAAATTTTATATTTTTATTAAAGTTTTGTTGGGGTGACAAAAAAAATTATATAACTTTTTTTTATTAAACCATTAATTTATGTTTTTTTGATCCTTTATTATGGAAAATAAGATTAAGT** |
| **Miridae sp.1** | **ACTTTATTTATGTTAATTATGTTGGGGTGACAGAAAAAATTATATAACTTTTTCTATTTTAGGCATTTATTTATGTTTTGATCTTTTATTAAAAAAATAGGATTAAGT** |
| **Miridae sp.3** | **ACTTTATTAATATTATTTATTGTTTTTATGGATTATTATAAAATGATATATTTATGTTAATTTTGTTGGGGTGACAAAAAAAATATTATAACTTTTTTTATTTTTTACATTTATTAATGTTTTTTTGATCCTATATTATGGATTATAAGATTAAGT** |
| **Miridae sp.2** | **ACTTTACTTTTCTTATTTTAGTTTTTTATTTGCAAATAATTTTTTATTATTTTAATATAAGTTTTATTGGGGTGATAAAAAAAATTTTATAACTTTTTTTATTTTTTTCATTTATTTATGCTTTTTTGATCCTTTATTATGGATAATAAGATTAAGT** |
| **Microvelia reticulata** | **AGTTTACTTTTTTTAAAAAATTATTTTTTTGGTTTTTAAATATATTTTTTTTGAAGAAAAAGTTTTGTTGGGGCGACAATAAAATTTATTTAACTTTTATTATTTTTATTCATTTATTTATGTTTTTTTGATCCAAAAATTTTGATTATAAGATTAACT** |
| **Bombus lapidarius** | **ATTTTATATTTATATTTGGAATTAAAATTTATTTTAAGTATAAGTATTTGATTGGGAGGATTAATAAATTTAATTAACTTTATTTAAAATATTAACTTTAATTAAAGAATTATTTTTGATCTTTTATTAAAAATTAAAAGAAAAAAT** |
| **Bombus soroeensis** | **ATTTTATATTATTAATTAAATTAAATATATTTTAATTATTAATATTTAATTGGGAGGATTGGTAAATTTGAATAACTTTATTTTAAAAGTAAACTTTAATTTGAGAATTTTAGTGATCTTGTAATATAAATTGAAAGATAAAAT** |
| **Acrididae sp.** | **GCTTAACATCTAAATTAGATATTTTTTTAAGATGGTTTATTTATGTTTAATGGTAATGTTTTGTTGGGGTGACATGAAGAATTAATAAACTCTTTATTATAAAATCATTAATTTATGTTTATTTTGATCCATAATTTATGATCATAAGATTAAGT** |
| **Anaphothrips obscurus** | **ACTTAATTATTTTTTAATTTTATTAAAAAAGGAATAAATTTTGCTGGGGAAGCAAAGAAAGAAAAATTTTCTTTTTTTTAAACAAAATTTTTGATTAATTGATCCTTTTTAAGAAAAAAGAATAAGT** |
| **Thripidae sp.** | **GCACTGGACGAGAAGACCCTATAGAACTTAAATTTTTAAAAAAATAAAAAAAAGAAAAATTTTACTGGGGAAGTAAAAAAACAAAACTTTTTTTTAAAAAAAACTTTTAAAAGATTATAAGATCCTTAAAAAAGAAAAAAGAATAAGT** |
| **Pacifastacus leniusculus** | **ACTTTATATTTTAAAATAATAGTTAGTTTTATTTAAGAGTTTTATTTTGAAATATTTTATTGGGGTGATAAAGATATAAATTAAAATAACTGTCTTTTTTTTTTACAATAATCTTTGAATTAATGATCCTAAGAAAGGAGTAAAAGATCAAGT** |
| **Palaemon adspersus** | **ACTTTATAAATGAAGTATGTAATCTCTAAATTATATTAAAATGTTGATAAGTATTTTATTTATTTCGTTGGGGCGACGTTGATATAATTTGTAACTGTCTAAATGAATAATATAATCGTCATTATACTTTGATCCTTCTTTGTGGATTATAAGACTAAGT** |
| **Asellus aquaticus** | **ACTTTACTAACAACTTATATTGTTATTGAACTAGTAGCAGGTTGTTGGTTAAGCTGGGGCGGCAAATATATAAACAATATTAATATTAAATATTTAAAATAGAAAATTAGAACTTGAGTTAAAAATTTAAACAAGT** |
| **Isopoda sp.** | **ACTTTACCCTTAGATCTAGATTTGCTTAGATATAATCTACTATAAGGGTTGAACTGGGGCGGTATTTATATAAACAATCTTATAATAAATATTTTACATAAATTTAAAGAACTCTACTAGGAAATTTAGAATAAGT** |
| **Polyphemus pediculus** | **GCTTTAATTTCTTTCTTTTTTAAGTTTTTTGAAGTAAGAGCAAAAAATGATAGTAAATTTTGGTTGGGGCGACAAGGAGTAAAAGTAACACTCTTTTAAATAAACATATATAAATGAAGTAATGATCCCCTTTGTGGGATTAAAAGACTCAGT** |
| **Arianta arbustorum** | **CTTTATTAATTTTGTTGGGGCGACAGAATTACAATATAAACTAATTTTATATTACAAGAAACGTAGGTGATAGAACAAGT** |
| **Cornu aspersum** | **CTTGCAAGTGTTTTTGTTGGGGCGACAGGATTACAATTAACTAATCTTATTTTACGAGACGATAAACAGAAAGAATAAGT** |
| **Enchytraeidae sp.** | **GCTTTATTTAACTTCTATAAATAATAGAATAAAATTCGGTTGGGGCGACCAAGGAAAAATCATCCTTATGTTAAATAGATAAATTTATCTAACCATAGATCCTAGTAAGATCATCAGAATTAGC** |
| **Aporrectodea tuberculata** | **GCTTTATTTTAATAAAAATTATAAATTTTTAATAAATTCGGTTGGGGCGACCAGGGAAATTATCAATCATCCCCTATAAAAAGATAAATTAATCTAAACTCTGACCCTTAATCAAGATCAATAAATCAAGC** |
| **Eiseniella tetraedra** | **GCTTAATTCTAATAAAAATATATATTTTAATAAAATTCGGTTGGGGCGACCAGGGAATACAAATCATCCCTATTCAAAAGATACATAAATCTTGAAAATGACCCTTAAACAAGATCACAAAAACAAGC** |
| **Lumbricus rubellus** | **GCTTAATTTAAACAAATACACAAAATTTACTAAAATTCGGTTGGGGCGACCAGGGAAATTATAAATCATCCCTAAATAAAAGACAAATTAGTCTATCTGCTGACCCTTAATTAAGATCACTAAAACAAGC** |
| **Chaetogaster diaphanus** | **GCTTTATTTTTAATATATAATAATAATATACAAAAATTCAGTTGGGGCGACTAAGGAAAAATCATCCTAAATTTTAAAGATAAACAAATCAATCAAAAGATCCTAAATTATAGATCAAAGAAATTAGC** |
| **Stylaria lacustris** | **GCTTTATTTTTAATACATAAACTATATGTAGAAAAATTCAGTTGGGGCGACTAAGGAAAAAATCATCCTAAAATTTATAGACGAACATGTCAATTCAAAGATCCTAAAAATAGATCAAAGAAATTAGC** |
| **Hydra circumcincta** | **GCTTTACTATAAATTTTCTTTCTTTTAAAATATAAAAATTATTTAATTTAGAAAATTTGGTAGTTTAGTTGGGGCGACTGTTTTTTAAAAATAACAAAAATAAGCAATATAATAATATTATTTATTGTATAATCAAACAATTTAACAATTACTATAGTAGGCTATAATGACCCGTTATTATATTAAAAAATAAATAACGAATAATTAATAAAAGC** |
| **Esox lucius** | **GCTTTAGACACCCGGCAGACCCTGTTAAGTAGCTGAACTATCAGATTAAAACAAAGCGGCCCCTGGCCTACATGTCTTCGGTTGGGGCGACCACGGGGGAAAACAAAGCCCCCACGAGGATTAAGGAAAACCTCCTTATAACCACGAGCGACAGCTCTAAGTCTCAGAACTTCTGACCAAAAAGATCCGACACCAGTCGATCAACGGACCAAGT** |
| **Pterois miles** | **GCTTTAGACACCAAGGAAGATCCTGTCAAAATCCACCAAAAATGGAAAGAACTAATGGATATCTCCTTCCCTAATGTCTTTGGTTGGGGCGACCGCGGGGGAACAAGAAGCCCCCACGTGGAATGGGAAAACACCTTTCCTACAGCCCAGAGCCACAGCTCGAGGCAACAGAACTTCTGACCATCAAGATCCGGCAAAGCCGATCAACGGACCGAGT** |
| **Canis lupus familiaris** | **GCTTTAATTAACTAACCCAAACTTATGGATACTAGATACCTACAAGGCATAACATAACACCATTATTATGAGTTAGCAATTTAGGTTGGGGTGACCTCGGAATATAAAAAAACTCCCGAGTGATTAAAATTTAGACCCACAAGTCAAAATACAACATCACTTATTGATCCAATAATTTTTGATCAACGGAACAAGT** |
| **Bos taurus** | **GCTTTAACTAACCAACCCAAAGAGAATAAATTTAACCATTAAGGAATAACAACAATCTCCATGAGTTGGTAGTTTCGGTTGGGGTGACCTCGGAGAATAAAAAATCCTCCGAGCGATTTTAAAGACTAGACCCACAAGTCAAATCACTCTATCGCTCATTGATCCAAAAACTTGATCAACGGAACAAGT** |
| **Ovis aries** | **GCTTTAACTAAGTAACTCAAGGAAAATAAATTCAACCACCAAGGGATAACAACACTCCTTATGAGTTAACAGTTTCGGTTGGGGTGACCTCGGAGAACAGAAAATCCTCCGAGCGATTTTAAAGACTAGACTAACAAGTCAAACCAAACCATCGCTTATTGATCCAAAAACTTGATCAACGGAACAAGT** |
| **Dama dama** | **GCTTTAACTACTTAGCCCAAAGAATCAAATTTTATCACCAAGGAAACAACAACACTCTTTATGGGTTAACAGCTTTGATTGGGGTGATCTCGGAGAATAAGAAATCCTCCGAGCGATTTTAAAGACTAGACCTACAAGTCGAATCACACAATCGCTTATTGATCCAAAAAATTGATCAACGGAACAAGT** |
| **Sus scrofa** | **GCTTTAATTAACTATTCCAAAAGTTAAACAACTCAACCACAAAGGGATAAAACATAACTTAACATGGACTAGCAATTTCGGTTGGGGTGACCTCGGAGTACAAAAAACCCTCCGAGTGATTTTAATCTAGACAAACCAGTCAAAATAACCATAACATCACTTATTGATCCAAAATTTTGATCAACGGAACAAGT** |
| **Equus caballus** | **GCTTTAATTAACTGATTCACAAAAAACAACACACAAACCTAACCTTCAGGGACAACAAAACTTTTGATTGAATCAGCAATTTCGGTTGGGGTGACCTCGGAGAACAAAACAACCTCCGAGTGATTTAAATCCAGACTAACCAGTCAAAATATATAATCACTTATTGATCCAAACCATTGATCAACGGAACAAGT** |
| **Homo sapiens** | **GCTTTAATTTATTAATGCAAACAGTACCTAACAAACCCACAGGTCCTAAACTACCAAACCTGCATTAAAAATTTCGGTTGGGGCGACCTCGGAGCAGAACCCAACCTCCGAGCAGTACATGCTAAGACTTCACCAGTCAAAGCGAACTACTATACTCAATTGATCCAATAACTTGACCAACGGAACAAGT** |

**Table S5**. Mock sample results, showing species ID and number of reads obtained. Data is given for COI and 16S separately. *) Only 99% match to species.

| COI | |
| --- | --- |
| Species | Count |
| *Cybister lateralimarginalis* | 1214401 |
| *Ilyocoris cimicoides* | 347 |
|  |  |
| % of total reads in mock | 99,98 |
|  |  |
| 16S | |
| *Dorcus* sp. | 717656 |
| *Ilyocoris cimicoides* | 136485 |
| *Lestes virens* | 18779 |
| *Argyroneta aquatica** | 26 |
|  |  |
| % of total reads in mock | 99,56 |

**Figure S1**. Workflow of sample processing, sequencing and bioinformatics analyses performed in the study.

**Figure S2**. Venn diagram showing overlap of arthropod families detected from metabarcoding with 16S and COI. 67 families were obtained in total, 47 unique families were obtained with COI while 7 unique families were obtained with 16S, and 11 families were obtained with both genes.

**Figure S3.** Boxplots showing richness of arthropod taxa found with eDNA in this project. A) COI, B) 16S. Plant names: Angeli (*Angelica archangelica*), Centau (*Centaurea jacea*), Daucus (*Daucus carota*), Echium (*Echium vulgare*), Eupato (*Eupatorium cannabinum*), Solida (*Solidago canadensis*), Tanace (*Tanacetum vulgare*). *) Transect samples collected with 10 m distance between each.

**Figure S4**. Species accumulation curves for PCR replicates. Data for COI species level. Expected mean species richness (blue line) and its standard deviation (turquoise area). Empty boxes are samples with the same taxa in all PCR replicates.

**Figure S5**. Species accumulation curves for PCR replicates. Data for 16S species level. Expected mean species richness (blue line) and its standard deviation (turquoise area). Empty boxes are samples with the same taxa in all PCR replicates.


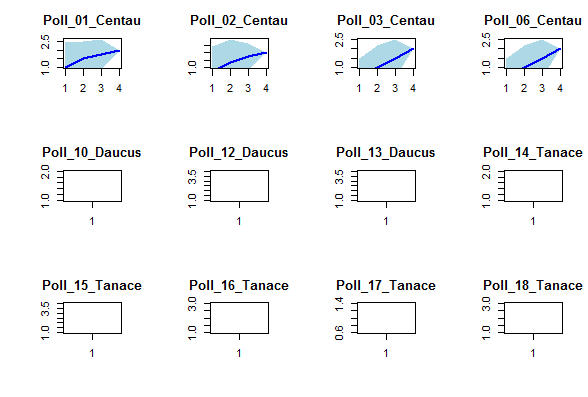


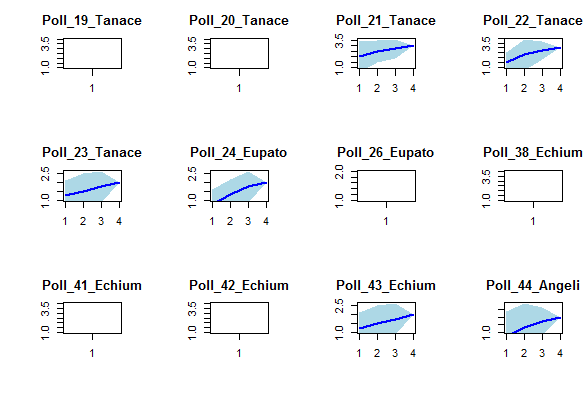


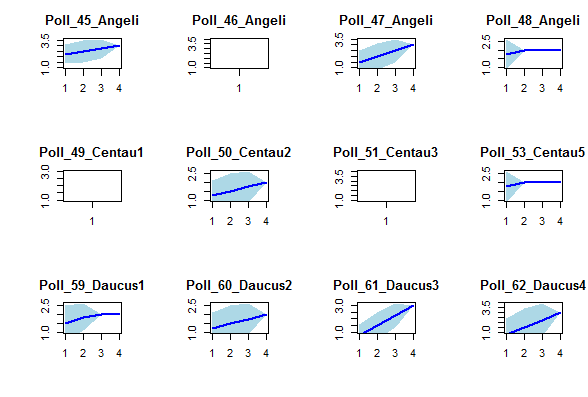


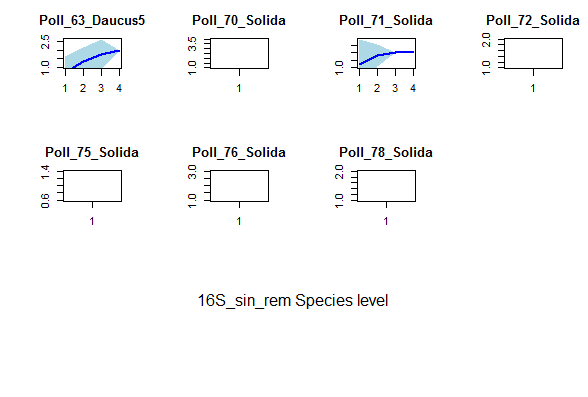


**Figure S6.** Rarefaction curves on the four individual PCR replicates. Data for COI. Figure shows the accumulated number of taxa recovered as a function of sequencing depth for each plant sample. Consecutive numbers inside the squares indicate the individual PCR replicates (four on each sample).

**Figure S7.** Rarefaction curves on the four individual PCR replicates. Data for 16S. Figure shows the accumulated number of taxa recovered as a function of sequencing depth for each plant sample. Consecutive numbers inside the squares indicate the individual PCR replicates (four on each sample).

**Figure S8**. Heatmap cluster analyses of the metabarcoding results for COI species level. Plant names: Angeli (*Angelica archangelica*), Centau (*Centaurea jacea*), Daucus (*Daucus carota*), Echium (*Echium vulgare*), Eupato (*Eupatorium cannabinum*), Solida (*Solidago canadensis*), Tanace (*Tanacetum vulgare*). *) Transect samples collected with 10 m distance between each.

**SUPPORTING REFERENCES**

Elbrecht, V., Taberlet, P., Dejean, T., Valentini, A., Usseglio-Polatera, P., Beisel, J.-N., … Leese, F.

(2016). Testing the potential of a ribosomal 16S marker for DNA metabarcoding of insects. *PeerJ*, *4*.

Gagné, R., & Jaschhof, M. (2017). A Catalog of the Cecidomyiidae (Diptera) of the World. Fourth

Edition. Digital. 762 pp.

Zeale, M. R. K., Butlin, R. K., Barker, G. L. A., Lees, D. C., & Jones, G. (2011). Taxon-specific

PCR for DNA barcoding arthropod prey in bat faeces. *Molecular Ecology Resources*, 11, 236–244.
